# Adalimumab versus placebo as add-on to standard therapy for non-infectious uveitis in the United Kingdom: the ASTUTE randomised controlled trial with treatment run-in

**DOI:** 10.1016/j.eclinm.2026.104240

**Published:** 2026-10-08

**Authors:** Andrew D. Dick, Barnaby C. Reeves, Nicholas A.V. Beare, Mandy Lui, Katie Pike, Sarah Baos, Lucy Culliford, Xuemin Zhu, Sarah Wordsworth, Annie Folkard, Kirsty Lanyon, Rositsa Koleva-Kolarova, Rachel C.M. Brierley, Tunde Peto, Savita Madhusudhan, Srilakshmi M. Sharma, ASTUTE study investigators, Simon Epps, Krishna Naruganahalli, Natasha Clark, Laura Bendrey, Helen Devonport, Sridevi Rajasekaran, Hassan Ahmed, Edward Hughes, Gabriela Ortiz, Dimitra Gkika, Erika Damato, Jonathan Virgo, Tasanee Braithwaite, Paraskevi Riga, Kanchan Bhan, Clare Howcroft, Mike Stockton, Michael Hisaund, Periyasamy Kumar, Lauren Rybicki, Nima Ghadiri, Goncalo Almeida, Hanna Bimah, Idris Haider, Carlos Pavesio, Sumita Phatak, Gerry Clare, Colin Jones, Chin Pey Yap, Archana Pradeep, Elizabeth Mathew, Theodora Gkika, Shabnam Raji, Janette Savage, Katharine S. Sears, Katherine Wheeler, Farid Afshar, Catherine Murphy, Ahmed Saad, Kerry Hebbron, Balini Balasubramaniam, Sawsan Elsheikh, Helen Hawkins, Yvonne Anyango, Richard Gale, Christine O'Dwyer, Sally Gilroy, Elizabeth McCullagh, Shakeel Herwitker, Suhail Kidi, Abby O'Connell, Mae Hazell, Sophie Coke-Norris, Laura Waugh, Robin Wickens, Beth Howden, Isabel Druiff, Sian Wells, Kelsey Harrendence, Rachel Todd, David Hutton, Jana Kirwan, Jo Berkeley, Vaishali Thaker, Kate Attfield, Subita Balaram Kuttikkatte, Karleigh Kelso, Clare Newell, Barbra Hamill

**Affiliations:** a School of Cellular and Molecular Medicine, University of Bristol, Bristol, BS8 1TD, UK; b Bristol Trials Centre, Bristol Medical School, University of Bristol, 1-5 Whiteladies Road, Bristol, BS8 1NU, UK; c Department of Eye and Vision Science, Institute of Life Course & Medical Sciences, University of Liverpool, Liverpool, L7 8TX, UK; d Health Economics Research Centre, Nuffield Department of Population Health, University of Oxford, Old Road Campus, Headington, Oxford, OX3 7LF, UK; e Birdshot Uveitis Society, PO Box 64996, London, SW20 2BL, UK; f School of Medicine, Dentistry and Biomedical Sciences, Centre for Public Health, Queen's University Belfast, Belfast, BT12 6BA, UK; g Liverpool Ophthalmic Reading Centre, St Paul's Eye Unit, Liverpool University Hospitals NHS Trust, Prescot Street, Liverpool, L7 8XP, UK; h Oxford Eye Hospital, John Radcliffe Hospital, Oxford University Hospitals NHS Foundation Trust, Headley Way, Oxford, OX3 9DU, UK; i NIHR Oxford Biomedical Research Centre, University of Oxford, Oxford, UK; j UCL Institute of Ophthalmology, University College London, London, EC1V 9EL, UK; k NIHR Biomedical Research Centre, Moorfields Eye Hospital, London, EC1V 2PD, UK; l Kennedy Institute of Rheumatology, University of Oxford, Old Road Campus, Headington, Oxford, OX3 7LF, UK

**Keywords:** Non-infectious uveitis, Adalimumab, TNF inhibitor, Randomised controlled trial, Treatment run-in, Cost-effectiveness

## Abstract

**Background:**

Immune-mediated non-infectious uveitis (NIU) is a leading cause of blindness in high-income countries. Adalimumab is the only licensed non-corticosteroid drug for non-infectious intermediate, posterior or panuveitis. We evaluated the efficacy, safety and cost-effectiveness of an adalimumab biosimilar as add-on therapy for a broad NIU population.

**Methods:**

This multicentre, placebo-controlled randomised trial recruited adults with sight-threatening or controlled NIU requiring oral prednisolone >5 mg/day from 18 UK NHS ophthalmology departments between 29 July 2021 and 7 June 2024. After a 16-week treatment run-in (TRI) with adalimumab, responders (prednisolone ≤5 mg/day, no active disease) were randomised 1:1 (parallel groups; concealed web-based allocation) to fortnightly subcutaneous 40 mg adalimumab (biosimilar) or placebo as add-on therapy; all but the trial statistician were masked. The primary outcome, time to treatment failure (TF; disease activity or prednisolone >5 mg/day), was analysed at 12 months by intention to treat. The health economic evaluation was pre-specified in the trial protocol; we estimated incremental cost per TF avoided, NHS perspective. Registered: ISRCTN31474800.

**Findings:**

182 participants completed the TRI, 119 (65.4%) responded, and 115 were randomised (60 adalimumab, 55 placebo). At 12 months, 31.4% (adalimumab) and 59.0% (placebo) had experienced TF (hazard ratio 0.42, 95% CI 0.23–0.75, p = 0.003). Among patients ineligible for NHS treatment (85%), the effect was similar (hazard ratio 0.37, 95% CI 0.20–0.71, p = 0.003). Adverse events were similarly frequent; two were serious, one per group. Incremental cost was £2320 per TF avoided.

**Interpretation:**

Adalimumab added to standard care reduces treatment failure in NIU, including in patients with macular oedema, unilateral disease, and steroid-dependent inactive uveitis, and is affordable. Future work should identify complementary treatments for disease refractory to adalimumab.

**Funding:**

Health Technology Assessment Programme, National Institute for Health and Care Research (NIHR); Biogen International GmbH (Baar, Switzerland) provided the placebo for the trial.

**Research in context**

**Evidence before this study**

Adalimumab is the only non-corticosteroid drug licensed for the treatment of immune mediated non-infectious uveitis (NIU). The VISUAL I and II trials established efficacy but excluded some patient groups. In the UK NHS, eligibility for treatment with adalimumab was limited to a selected population with NIU. We searched PubMed, the Cochrane Library, the WHO International Clinical Trials Registry Platform (ICTRP) and ClinicalTrials.gov, together with the proceedings of major international ophthalmology meetings, from April 2016 to April 2026 with no language restriction, combining population terms (“non-infectious uveitis”, “ocular inflammation”) with intervention and study-design terms (“adalimumab”, “investigational medicinal product”, “randomised/randomized controlled trial”, “clinical trial”) to identify randomised trials of adalimumab in non-infectious uveitis. Beyond the VISUAL trials, the only further adult randomised trial of adalimumab in non-infectious uveitis we identified was ADVISE (intermediate, posterior and panuveitis), which compared adalimumab directly with conventional immunosuppression.

**Added value of this study**

This randomised, placebo-controlled trial included a wider group of patients with NIU than previously studied. A 16-week treatment run-in with corticosteroids tapered to 5 mg/day or less of oral prednisolone identified patients who responded to adalimumab and provided an objectively defined criterion for stopping treatment, absent in clinical practice. At the end of the treatment run-in, responders were randomised to adalimumab or placebo. Critically, 85% of participants would have been ineligible for treatment with adalimumab in the NHS but benefited at least as much as those who were eligible, and to a similar degree as in the VISUAL trials. Patients who were ineligible for treatment in the NHS included those with steroid-dependent inactive disease, unilateral isolated uveitis, mild to moderate disease activity in the vitreous, macular oedema alone or naive to standard immunosuppression. The trial also provides information about the safety of adalimumab over two years of treatment and information on the cost-effectiveness of adalimumab compared to placebo.

**Implications of all the available evidence**

This trial demonstrates that adalimumab can be effectively and safely used as an add-on therapy for NIU in a substantially broader population of patients than currently recognised or for whom it is recommended in the NHS. Compared to placebo, adalimumab reduced treatment failure and enabled the reduction of corticosteroids to doses which minimise toxicity, aligned with patient preference. These results suggest that the recommendation of the UK National Institute for Health and Care Excellence on the use of adalimumab to treat NIU should be reviewed. The ongoing open-label extension should establish the durability of response and longer-term safety. Future work should identify additional and complementary treatments for the subset of patients whose disease remains refractory despite adalimumab.

## Introduction

Immune-mediated non-infectious uveitis (NIU) is a group of conditions defined anatomically and with overlapping immune mediated pathways. Altogether they represent the third leading cause of preventable blindness in working-age populations in high-income countries, affecting approximately 115 per 100,000 individuals with an annual incidence of 52 per 100,000.^1^^,^^2^ Although regarded as a rare disease, its socioeconomic burden is disproportionately high; 70–90% of affected individuals are of working age and 43% are at risk of job loss or have already lost their jobs due to visual impairment.^3^

Conventional immunomodulatory therapies (IMTs) are used ‘off label’ as part of usual care. However, 40% of patients with sight-threatening disease do not achieve disease control with these steroid sparing agents or are intolerant, necessitating parallel treatment with high doses of corticosteroids.^4^, ^5^, ^6^, ^7^ Studies indicate that over 60% of patients with sight-threatening NIU require corticosteroid doses associated with significant morbidity.^8^, ^9^, ^10^

Substantial evidence from experimental animal models where immune responses to retinal self-antigens replicate the clinical phenotype supports an autoimmune or immune-mediated pathogenesis. Hence, we use the term immune-mediated non-infectious uveitis (NIU) throughout this manuscript.^11^ Tumour necrosis factor alpha (TNFα) is a critical cytokine in the inflammatory cascade which mediates retinal tissue damage in uveitis by mononuclear cell trafficking and initiating numerous inflammatory pathways.^12^

The VISUAL trials reported an important therapeutic advance in the treatment of ocular inflammation.^13^^,^^14^ These trials demonstrated that adalimumab, a fully human monoclonal antibody targeting TNFα, was effective in delaying treatment failure (TF) compared to placebo in active (VISUAL I) and inactive steroid-dependent NIU (VISUAL II).^13^^,^^14^

Adalimumab is the only licensed biologic therapy for non-infectious uveitis and the only alternative to corticosteroids which is licensed for this indication, emphasising the importance of patient access to this effective treatment. Despite proven efficacy, critical gaps remain in the evidence base. The VISUAL trials did not evaluate adalimumab as an add-on therapy, the most common clinical scenario when first-line agents fail. Cystoid macular oedema (CMO), the leading cause of uveitis-related visual loss, was neither an inclusion nor a treatment-failure criterion. A high level of vitreous inflammation was required for a patient to be eligible for inclusion. These design limitations have translated into restrictive access criteria globally. In the United Kingdom (UK), the National Institute for Health and Care Excellence (NICE) technology appraisal of adalimumab to treat NIU excluded patients with unilateral disease without systemic inflammation or steroid-dependent inactive uveitis or who are naive to standard IMT from the treatment recommendation.^15^

The Adalimumab versus placebo as add-on to Standard Therapy for autoimmune Uveitis, Tolerability, Effectiveness and cost-effectiveness (ASTUTE) trial was designed to address these evidence gaps: (1) evaluation of adalimumab as an add-on to standard care, reflecting real-world practice; (2) eligibility criteria to include patients excluded from the current NICE treatment recommendation^15^; (3) a 16-week treatment run-in to provide a criterion for stopping treatment, maximising the likelihood of subsequent benefit and minimising the risk of drug-related harm; and (4) use of a recently licensed adalimumab biosimilar (Imraldi, Biogen GmbH) for the first time in an ocular inflammation trial.

## Methods

### Study design

The ASTUTE study is a multi-centre, parallel group, placebo-controlled, randomised trial (RCT) with a 16-week treatment run-in (TRI) to evaluate the efficacy and cost-effectiveness of adalimumab compared to placebo. An open-label extension (OLE) was added when impending expiry of the placebo pens on 31 January 2025 meant placebo control was no longer possible. The manufacturer could not extend the expiry date or provide a new supply. The OLE continues and details are not reported here. At the end of the TRI, participants were assessed for their response to adalimumab and only those participants classified as having responded to treatment were eligible to be randomised. AF led a patient and public involvement group; members (see Acknowledgements) advised about the acceptability of key features of the trial design.

The trial protocol is included in the Supplementary Appendix and version 8 was published.^16^ All protocol versions including the current one are available at: https://www.fundingawards.nihr.ac.uk/award/16/24/09. Since publication of the protocol, substantive amendments have changed the maximum and minimum durations of participation and added the OLE.

### Protocol and statistical analysis plan

The protocol is available to download from https://www.fundingawards.nihr.ac.uk/award/16/24/09. The statistical analysis plan is available from: https://research-information.bris.ac.uk/en/publications/the-astute-trial-adalimumab-vs-placebo-as-add-on-to-standard-ther/.

### Ethics

The ASTUTE trial was conducted in accordance with the principles of the Declaration of Helsinki and Good Clinical Practice. Approvals were received from the Medicines and Healthcare Products Regulatory Agency (EudraCT: 2020-000754-97), South Central Oxford B Research Ethics Committee (20/SC/0153) and the Health Research Authority (IRAS: 271051). The trial was registered as ISRCTN31474800 (https://doi.org/10.1186/ISRCTN31474800) on 14/04/2020, sponsored by Bristol NHS Foundation Trust and managed by the Bristol Trials Centre, University of Bristol (Bristol, UK). Written informed consent was obtained from all participants before any trial procedures.

### Study participants and setting

Recruitment of participants was conducted at 18 NHS tertiary UK ophthalmology departments, which have specialist uveitis services. The target population was adults with active or controlled NIU. We described NIU by standard anatomic descriptors of intermediate, posterior and pan uveitis, because these features cause most sight loss.^10^^,^^17^ We intended that these descriptors would allow treatment results to be applied across the range of uveitic diseases, increasing applicability in patients with NIU who do not respond to usual care.^13^^,^^14^^,^^18^^,^^19^ We recognised that broadening the pool of eligible participants might mean the intervention was ineffective for some, but expected the TRI design to offset this by allowing only ‘responders’ to be randomised. Thus, we aimed to recruit to the TRI any patient in the following groups for whom add-on adalimumab was clinically indicated: (1) patients presenting with active sight-threatening NIU in either or both eyes and taking (or about to be started on) oral prednisolone >5 mg/day; (2) patients already being treated for NIU on >5 mg/day of oral prednisolone alongside other conventional IMTs as required. Oral prednisolone was tapered toward ≤5 mg/day over the 16-week TRI; in keeping with the pragmatic design the rate of reduction was guided by disease activity and clinician judgement rather than a fixed schedule. Participant sex (female/male) was recorded at enrolment from clinical records; gender identity was not separately collected. Ethnicity was self-reported by participants and recorded on the case report form using standard categories. At the end of the TRI, participants were classified as “responders” or not; responders had to have had oral prednisolone tapered to 5 mg/day or less with no sign of active disease.

#### Eligibility criteria for starting the TRI

Patients aged ≥18 years were eligible to take part if they had any of the following: (a) active sight-threatening NIU (active inflammatory chorioretinal lesions, central macular thickness (CMT) > 320 microns,^17^ evidence of retinal vasculitis,^17^ vitreous haze >0.5) and prescribed (already taking or being started on) oral prednisolone >5 mg/day, OR (b) controlled NIU and prescribed oral prednisolone >5 mg/day.

Reason for ineligibility included: controlled NIU and oral prednisolone ≤5 mg/day; medication with another biologic drug; medication with an anti-TNF drug within the previous 90 days; a dexamethasone implant, fluocinolone acetonide implant or intravitreal steroid injection, or periocular steroid within the previous 12 weeks (or within 18 months of a fluocinolone acetonide implant if NIU in remission); at risk of pregnancy; other diseases or circumstances in which adalimumab was clinically inappropriate. For full details, see Supplementary File, Section 1.1.

### Intervention and comparator

The intervention under evaluation was fortnightly subcutaneous 40 mg injections of an adalimumab biosimilar (Imraldi™, a licensed adalimumab biosimilar; Biogen International GmbH, Baar, Switzerland; www.biogengmbh.com) using pen devices. These injections were prescribed open-label during the TRI, with no comparator.

After the TRI and randomisation, participants administered a 40 mg injection of adalimumab or placebo once every fortnight. The placebo was packaged in identical pen devices, provided by Biogen International. The placebo contained all excipients but no active drug. Prescription of concomitant IMTs was permitted and recorded at all study visits. Prescription of topical corticosteroid therapy was permitted to manage anterior chamber inflammation and did not contribute to the primary outcome definition. This was a deliberate, pragmatic feature: anterior chamber activity was managed with topical corticosteroid (an isolated flare would not prompt withdrawal of a biologic in practice), whereas escalation to oral prednisolone >5 mg/day to control disease from any cause, including anterior chamber inflammation, was a treatment failure criterion. Clinically significant anterior involvement was therefore captured, while transient activity manageable with drops did not artificially inflate the failure rate. For details of over-labelling of the pen devices and their distribution, see Supplementary File, Section 1.2.

### Study outcomes

The primary outcome was time to the first TF. Any of the following signs of active disease in either eye, as judged by a participant's ophthalmologist, constituted TF, unless they occurred within 14 days of having intraocular surgery:

i. ≥15 letters decrease in best corrected visual acuity (BCVA; measured using Early Treatment of Diabetic Retinopathy Study charts), compared to BCVA measured at the 16-week TRI visit;
ii. New active inflammatory chorioretinal lesions, based on slit-lamp biomicroscopy examination and reviewing fundus colour and autofluorescence images;
iii. >20% increase in central macular thickness (CMT), compared to CMT at the 16-week TRI visit (CMT is measured on a foveal-centred optical coherence tomography (OCT) scan by automated algorithm);
iv. Onset or worsening of retinal vasculitis, based on fundus fluorescein angiogram;
v. Two step worsening of vitreous haze score on the Standardization of Uveitis Nomenclature (SUN) scale compared to the score at the 16-week TRI visit;^17^
vi. Prescription of >5 mg/day oral prednisolone to maintain uveitis disease control (i.e. to avert relapse before any of the above criteria for disease activity were satisfied (i-v)). This did not apply if more than 5 mg/day of oral prednisolone was prescribed for a systemic manifestation or co-morbidity with none of i-v being present.

Secondary outcomes included: health related quality of life (HRQoL) (EQ-5D-5L questionnaire,^20^ patient-reported symptoms of side-effects and patient-reported visual function^21^); BCVA; CMT; adverse events (AEs); individual TF components; employment status and resource use. AEs including serious adverse events (SAEs) were recorded, coded (version 28.1, Medical Dictionary for Regulatory Activities (MedDRA)) and reported in accordance with Good Clinical Practice guidelines and the Sponsor's Research Safety Reporting Standard Operating Procedure.

### Study procedures

Participants gave written informed consent to participate. The schedule of visits was designed to reflect clinical practice: in the TRI, review at 4–5 weeks, 8–9 weeks (by telephone only) and 16–17 weeks, when response to treatment was assessed; in the RCT at 12-weekly intervals up to 48 weeks and 16-weekly intervals thereafter. Additional visits could be scheduled for clinical care if deemed necessary by the investigator. Study treatment was initiated with a loading phase, starting with two injections at week 0, one injection at week 1 and subsequently one injection fortnightly. Fortnightly injections of drug or placebo continued for between 12 weeks and 170 weeks (about 3–40 months). TF and all secondary outcomes were assessed at each visit. Central subfield thickness used for the treatment-failure criterion was derived by the automated algorithm on a foveal-centred spectral-domain OCT scan and reviewed by the treating ophthalmologist. In the event of TF in either eye during the trial, the participant was withdrawn from the trial and invited to repeat the TRI; see Supplementary File, Section 1.3 for details.

If an injection was missed, participants were instructed to administer the dose as soon as possible and then resume the original fortnightly dosing regimen, that is the subsequent injection was to be administered two weeks after the date on which the missed dose had been due. Delayed injections could be administered up until two days before the next was due, but not on consecutive days. Serum samples and retinal images were collected at some visits during the trial for future investigation of markers of treatment response and failure.^16^

### Sample size

The sample size was set at 174 randomised participants (87 per treatment group), which provided 90% power to detect a hazard ratio (HR) of 0.5, assuming 27% of the placebo group were free from TF 12 months after randomisation, 5% two-sided significance levels and 10% loss to follow-up in the first 12 months. The target HR was agreed by clinical members of the study team, and both the target HR and placebo group rates are consistent with two previous trials of adalimumab.^13^^,^^14^ We also expected the effect size of adalimumab to be increased due to randomising only those participants who responded to the drug in the TRI.

### Randomisation and masking

Participants were randomly assigned (1:1) to receive either adalimumab or placebo using a secure custom-designed internet-based randomisation system built into the study database. The random allocations were computer-generated by the trial statistician in blocks of varying size (unknown to trial personnel) and stratified by centre, before the recruitment started. The allocations were embedded in the trial database and concealed from all clinical and research personnel until a participant had been recruited, at which point a unique code was revealed to indicate the pens to be dispensed.

All participants, their clinical care team (ophthalmologist and other members of eye care team) and research nurse(s) responsible for participant follow-up, were masked to participants’ allocations. Except for the trial statistician, clinical trials staff managing the trial were also masked to allocation. Allocation was unmasked at the final RCT visit, as participants transitioned to the OLE or withdrew.

### Statistical analysis

Formal analyses are reported here for the trial phase 1, which was until a participant experienced a TF, completed follow-up in this trial phase or withdrew from the study. Analyses were performed on an intention-to-treat (ITT) basis according to a detailed pre-specified statistical analysis plan (SAP).^22^

The effect estimate for adalimumab versus placebo for TF was estimated as a HR and 95% Confidence Interval (CI) using a Cox proportional hazards model, adjusted for centre as a shared frailty term. The proportional hazards assumption was confirmed graphically and statistically.

Outcomes measured longitudinally (HRQoL, BCVA and retinal morphology) were analysed using mixed effects methods. Outcomes were modelled jointly with time to treatment failure (Merlin models in Stata^23^); this analysis technique takes into account different follow-up times across participants. Time was fitted as a continuous measure, exploring different parameterisations and including treatment by time interactions if significant at the 10% level, to allow changes in treatment effect with time to be described, where appropriate. Analyses were adjusted for baseline measures (measured at the end of TRI) and centre size (three strata, as centre was not estimable as a random effect) as fixed effects, and participant as a random effect. Summary statistics for BCVA and retinal morphology outcomes are reported for left and right eyes separately. Models included data from both eyes with an “eye” fixed effect allowing for correlation within patients; the treatment effect is estimated averaging across both eyes. The symptoms of side effects were categorised as any or none; the effect of adalimumab on this outcome was estimated as an odds ratio and 95% CI, using a logistic regression mixed model. All other longitudinal outcomes were estimated as mean differences or geometric mean ratios (depending on distribution) with 95% CIs, using linear regression mixed models.

AEs were coded using MedDRA into System Organ Classes (SOCs) and Preferred Terms. Events were summarised by severity and the treatment received. The effect of adalimumab on incidence rates of two prespecified groups of events of interest, namely (i) infections and infestations, including COVID-19 and (ii) skin and subcutaneous tissue disorders, was estimated using Poisson models accounting for differing exposure times, adjusting for centre size (three strata), with treatment effects estimated as incidence rate ratios and 95% CIs.

A subgroup analysis (pre-specified in the SAP but not the study protocol) was undertaken comparing outcomes for participants who were eligible to receive adalimumab under the current NICE recommendation^15^ with those who were ineligible; subgroup effects were investigated by adding subgroup by treatment interaction terms to the primary outcome model. A per-protocol analysis was performed as a pre-specified sensitivity analysis.

All other secondary outcomes (TF components, change in employment status) were described, with no formal treatment comparisons performed.

Statistical analysis was performed using Stata release 18 (StataCorp, College Station, TX, USA); joint longitudinal and time-to-event outcomes were modelled using the merlin package. An independent Data Monitoring and Safety Committee reviewed trial safety data periodically throughout the trial, and an independent Trial Steering Committee provided overall supervision of the trial on behalf of the sponsor and funder.

The health economic evaluation was pre-specified in the trial protocol.^16^ In the cost-effectiveness analysis, we estimated the incremental cost per treatment failure avoided with adalimumab versus placebo in the RCT, from the NHS perspective. Costs included study intervention (adalimumab), monitoring visits and clinical examinations, concomitant medications and the management of AEs and SAEs. All costs were reported in GBP (£) for the financial year 2023–2024.^24^, ^25^, ^26^, ^27^ Costs beyond year one were discounted at 3.5% per year.^28^ Unit costs are listed in Table ST1; full details are in the Supplementary File, Section 1.4. A cost-utility analysis (which will include HRQoL) will be reported separately. Results are presented as total healthcare costs from randomisation until TF or end of RCT phase 1 and an incremental cost-effectiveness ratio per TF avoided.

### Role of the funding source

The funder, sponsor and Biogen International GmbH (Baar, Switzerland) had no role in study design, data collection, data analysis, data interpretation or writing of the report; Biogen GmbH commented on the draft manuscript with respect to factual accuracy.

## Results

### Participant flow and recruitment

Participants were recruited to the TRI between 29 July 2021 and 7 June 2024, and randomised between 21 December 2021 and 16 October 2024. Study recruitment to the TRI was stopped early due to expiry of the placebo pens on 31 January 2025; Biogen was unable to extend the expiry date or provide a new stock of placebo pens. In total, 323 patients were screened, of whom 202 enrolled in the TRI, 182 completed the TRI and 119 (65.4%) responded to treatment (Fig. 1). Four patients withdrew from the study before randomisation and 115 participants were randomised, 60 to adalimumab and 55 to placebo; 50 participants experienced TF in RCT phase 1 (19 in the adalimumab group and 31 in the placebo group). Details of participants who continued to participate after the first TF are shown in Figure SF1 (Supplementary File). All 115 randomised participants were included in the analysis population. Ten participants withdrew their consent after randomisation (five in adalimumab group and five in placebo), see Table ST2 (Supplementary File). The median follow-up time was 252 days (IQR 91–448), 331 days in the adalimumab group (IQR 148–541) and 175 days in the placebo group (IQR 89–447).

**Fig. 1:**
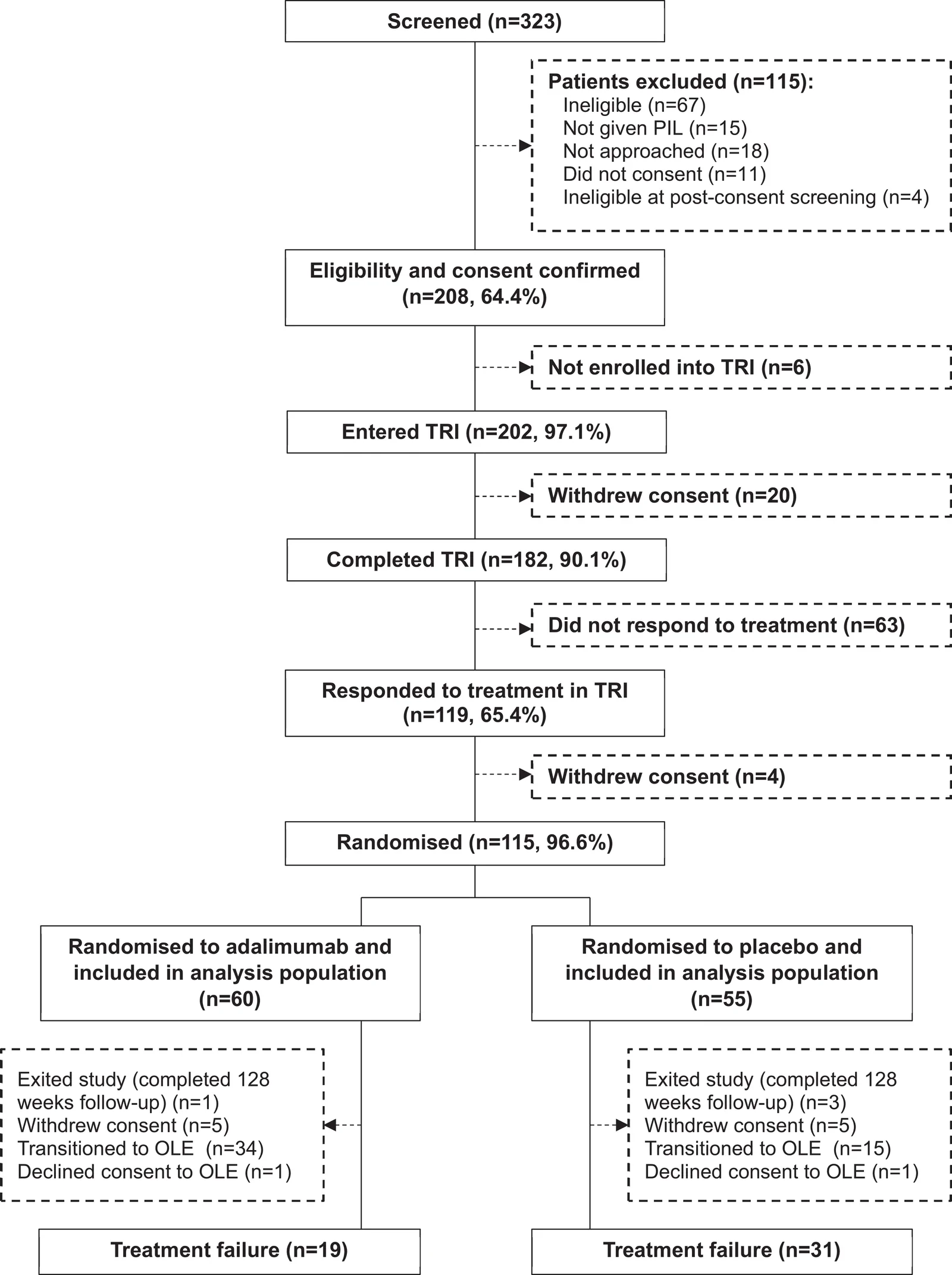
Participant flow. Numbers of participants screened, entering the 16-week treatment run-in (TRI), responding to treatment, randomised to adalimumab or placebo and included in the analysis population, with reasons for exclusion or withdrawal at each stage. OLE, open-label extension; TRI, treatment run-in.

### Delivery of intervention and comparator

Adherence with the allocated intervention was good; 96.7% (58/60) of participants in the adalimumab group and 90.9% (50/55) in the placebo group were classified as having no major protocol deviation (Table ST3, Supplementary File). In the RCT, 91.3% (211/231) of visits in the adalimumab group and 85.9% (140/163) in the placebo group fell within the pre-specified visit windows (Table ST4, Supplementary File) and all scheduled injections were received at 84.4% (195/231) and 81.0% (132/163) of due visits in the adalimumab and placebo groups, respectively (Table ST5, Supplementary File).

### Baseline characteristics

Key baseline characteristics of the randomised trial participants, assessed at the time of entry to the TRI, are described in Table 1; additional baseline information is shown in Table ST6, demonstrating the diversity of the study population with respect to specific uveitides. The median age of participants was 50.4 years (IQR 37.3–58.0), 43/115 (37.4%) were female and 102/115 (88.7%) were white/Caucasian. Table ST7 shows baseline information for the 119 responders and 63 non-responders completing the TRI. Participants who responded to treatment in the TRI were more likely to be younger, have higher BCVA and lower central subfield thickness at baseline, have no macular oedema, epiretinal membrane, vitreous haze or cystoid spaces present, be on lower prednisolone doses and be on immunosuppressants.

**Table 1:** Baseline characteristics of randomised participants (assessed at TRI entry) by randomised group.

	Adalimumab (n = 60)	Placebo (n = 55)
Demographic characteristics		
Sex (female); n (%)	21 (35.0%)	22 (40.0%)
Sex (male); n (%)	39 (65.0%)	33 (60.0%)
Age (years); median (IQR)	50.5 (39.3, 58.0)	50.4 (37.0, 57.2)
Ethnicity (White or Caucasian); n (%)^a^	54 (90.0%)	48 (87.3%)
Minoritised ethnic groups; n (%)	6 (10.0%)	7 (12.7%)
Weight (kg); median (IQR)	81.0 (67.5, 93.0)	87.0 (70.0, 95.0)
Disease activity status—n (%)		
Bilateral active	39 (65.0%)	36 (65.5%)
Unilateral active	8 (13.3%)	5 (9.1%)
Bilateral inactive (steroid-dependent)	12 (20.0%)	13 (23.6%)
Unilateral inactive (steroid-dependent)	1 (1.7%)	1 (1.8%)
Anatomical classification—n (%)		
Anterior uveitis^b^	9 (15.0%)	1 (1.8%)
Intermediate uveitis	11 (18.3%)	8 (14.5%)
Posterior uveitis	33 (55.0%)	30 (54.5%)
Panuveitis	5 (8.3%)	15 (27.3%)
Other	2 (3.3%)	1 (1.8%)
Most common diagnoses—n (%)		
Birdshot retinochoroiditis	18 (30.0%)	17 (30.9%)
Undifferentiated panuveitis	5 (8.3%)	10 (18.1%)
Undifferentiated intermediate uveitis	5 (8.3%)	5 (9.1%)
Retinal vasculitis	4 (6.7%)	3 (5.5%)
Baseline ocular status		
BCVA, Right eye ETDRS letters,^c^ median (IQR)	85.0 (77.5, 89.5)	84.0 (76.0, 89.0)
BCVA, Left eye ETDRS letters,^d^ median (IQR)	84.0 (79.0, 87.0)	84.0 (77.0, 87.0)
Anterior chamber cells ≥1+ in at least one eye (%)	5 (8.3%)	11 (20.0%)
Both eyes	2 (3.3%)	5 (9.1%)
One eye	3 (5.0%)	6 (10.9%)
Vitreous haze grade ≥1+ in at least one eye (%)	7 (11.7%)	3 (5.5%)
Both eyes	5 (8.3%)	1 (1.8%)
One eye	2 (3.3%)	2 (3.6%)
Cystoid macular oedema in at least one eye (%)	15 (25.0%)	15 (27.3%)
Both eyes	7 (11.7%)	7 (12.7%)
One eye	8 (13.3%)	8 (14.5%)
Central macular thickness: right eye, μm	274 (249, 308)	291 (261, 310)
Central macular thickness: left eye, μm	287 (252, 319)	286 (254, 302)
Active chorioretinal lesions in at least one eye n (%)	19 (31.7%)	16 (29.1%)
Both eyes	16 (26.7%)	14 (25.5%)
One eye	3 (5.0%)	2 (3.6%)
Retinal vasculitis—n (%)	27 (45.0%)	19 (34.5%)
Both eyes	24 (40.0%)	16 (29.1%)
One eye	3 (5.0%)	3 (5.5%)
Did not meet current NICE TA460 criteria—n (%)	51 (85.0%)	47 (85.5%)
Medications at baseline		
Oral prednisolone at TRI entry, mg/day, median (IQR)	10.0 (7.5, 20.0)	12.5 (10.0, 20.0)
Concomitant immunosuppression, n (%)	33 (55.0%)	26 (47.3%)
Mycophenolate mofetil	24 (40.0%)	15 (27.3%)
Methotrexate	7 (11.7%)	7 (12.7%)
Azathioprine	1 (1.7%)	3 (5.5%)
Ciclosporin	1 (1.7%)	1 (1.8%)
Tacrolimus	4 (6.7%)	1 (1.8%)
Other	0 (0.0%)	0 (0.0%)

Abbreviations: BCVA, best-corrected visual acuity; ETDRS, Early Treatment of Diabetic Retinopathy Study; IQR, interquartile range; NICE, National Institute for Health and Care Excellence; TA460, technology appraisal 460; TRI, treatment run-in; μm, micrometres.

a Minoritised (non-White) ethnic groups comprised Black/Black British (n = 6), Asian/Asian British (n = 5) and multiple ethnic group (n = 2).

b Participants with a primary anterior-uveitis classification were eligible only by virtue of co-existing cystoid macular oedema (n = 9) or a previous history of intermediate uveitis (n = 1); none had isolated anterior uveitis.

c 2 patients had BCVA of 0 (one adalimumab, one placebo), with vision at the level of counting fingers (CF) and hand movement (HM) respectively.

d 1 patient had BCVA of 0 (placebo group), with vision at the level of light perception (LP).

### Numbers analysed, outcomes and estimation

#### Primary outcome

At 12 months, an estimated 31.4% of 60 participants randomised to adalimumab had experienced TF, compared to 59.0% of 55 participants randomised to placebo (Fig. 2). More frequent and earlier TFs in the placebo group explain the difference between groups in the median duration of follow-up reported above. The HR for the effect of adalimumab versus placebo is 0.42 (95% CI 0.23–0.75, p = 0.003). For the subgroup analysis, 98 participants were identified as ineligible for adalimumab treatment under the current NICE technology appraisal.^15^ A treatment estimate for these patients is: 30.0% of adalimumab participants and 60.0% of placebo participants experienced TF at 12 months, HR 0.37 (95% CI 0.20–0.71, p = 0.003). A sensitivity analysis based on the per protocol population (see Supplementary File Table ST8) gave very similar results (HR = 0.39, 95% CI 0.21–0.72, p = 0.002).

**Fig. 2:**
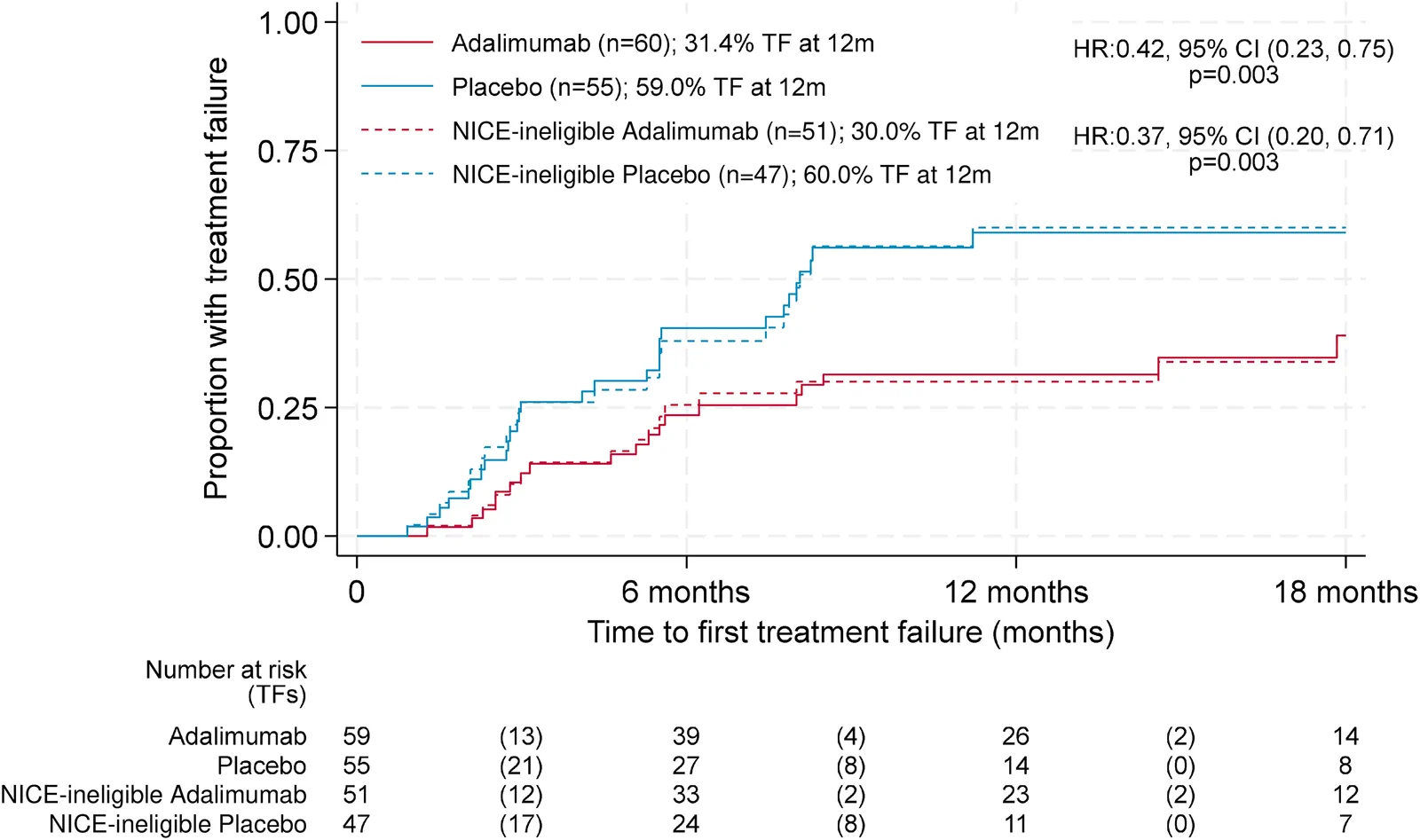
Primary outcome: time to treatment failure. Kaplan–Meier estimates of the proportion of participants with treatment failure over time, for the adalimumab and placebo groups and for the NICE-ineligible subgroups, with the numbers at risk (and cumulative treatment failures) shown beneath the x-axis. Treatment failure was defined as signs of disease activity or a requirement for oral prednisolone >5 mg/day. Hazard ratios were estimated from Cox proportional-hazards models: whole population 0.42 (95% CI 0.23–0.75, p = 0.003); NICE-ineligible subgroup 0.37 (95% CI 0.20–0.71, p = 0.003). CI, confidence interval; NICE, National Institute for Health and Care Excellence.

#### Secondary outcomes

Individual components of the TF outcome are described in the Supplementary File (Table ST9). The most frequently observed component was prescription of prednisolone >5 mg/day to maintain remission from uveitis.

Longitudinal outcomes are summarised in Figs. 3 and 4 and Table ST10 (Supplementary File). Comparisons of longitudinal outcomes mainly used data preceding TF, which were from participants still in remission except for final RCT visits for participants who experienced TF. For patient-reported visual function, better outcomes were seen with adalimumab than placebo, with an increasing effect over time (statistically significant after week 48). For other outcomes, most point estimates favour adalimumab but differences are not statistically significant: EQ-5D utility score mean difference (MD) 0.027, 95% CI −0.020 to 0.074, where a higher score indicates better HRQoL; EQ-5D visual analogue score MD −1.10, 95% CI −4.92 to 2.72, where a higher score indicates better HRQoL; patient reported visual function effect increases over time, the geometric mean ratio (GMR) at week 80 is 0.56, 95% CI 0.36–0.86, where a lower score indicates better HRQoL; symptoms of side effects score, odds ratio (OR) for any side effects 0.47, 95% CI 0.17–1.27; BCVA MD 0.88, 95% CI −0.82 to 2.57, where a higher score indicates better vision; CMT MD −4.03, 95% CI −8.25 to 0.19, where a higher score indicates poorer vision. There were few changes in employment status (Table ST11); the net change was that four participants lost employment, three in the adalimumab group and one in the placebo group.

**Fig. 3:**
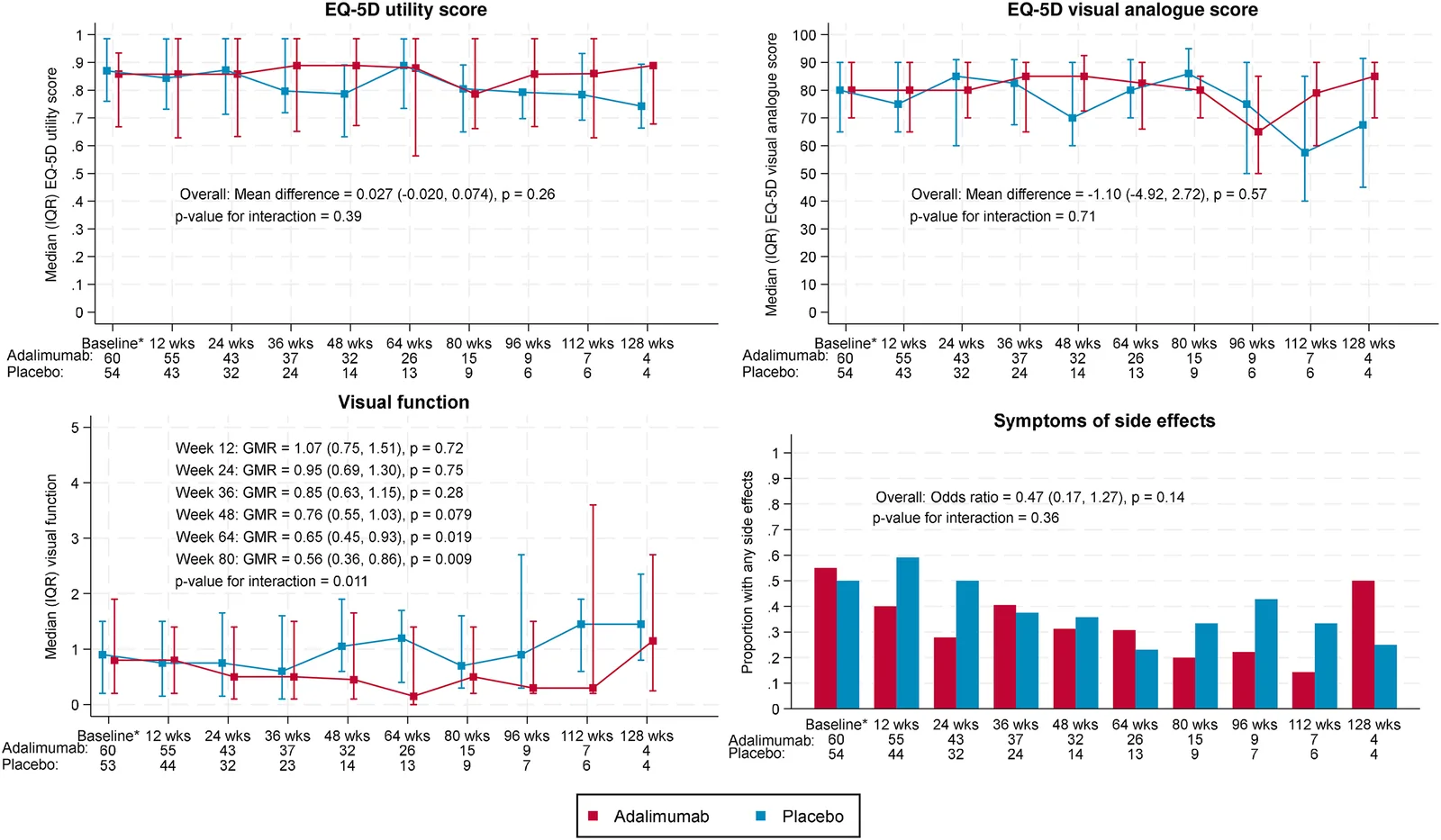
Quality of life longitudinal outcomes. Median (IQR) EQ-5D utility score, EQ-5D visual analogue score, patient-reported visual function, and the proportion reporting any side-effect symptoms, by treatment group over time. In the EQ-5D and visual-function panels, points show the median and error bars the interquartile range (IQR); the side-effects panel shows the proportion of participants reporting any symptoms. ∗Baseline is the end of the treatment run-in (TRI). For the EQ-5D utility and visual analogue scores a higher score indicates better quality of life; for visual function a higher score indicates more vision-related quality-of-life problems. Treatment effects shown within the panels are the overall mean difference (EQ-5D utility and visual analogue scores), geometric mean ratio (visual function) or odds ratio (any side-effect symptoms), each with its 95% confidence interval and p-value; the p-value for interaction tests whether the treatment effect changed over time. CI, confidence interval; GMR, geometric mean ratio; IQR, interquartile range.

**Fig. 4:**
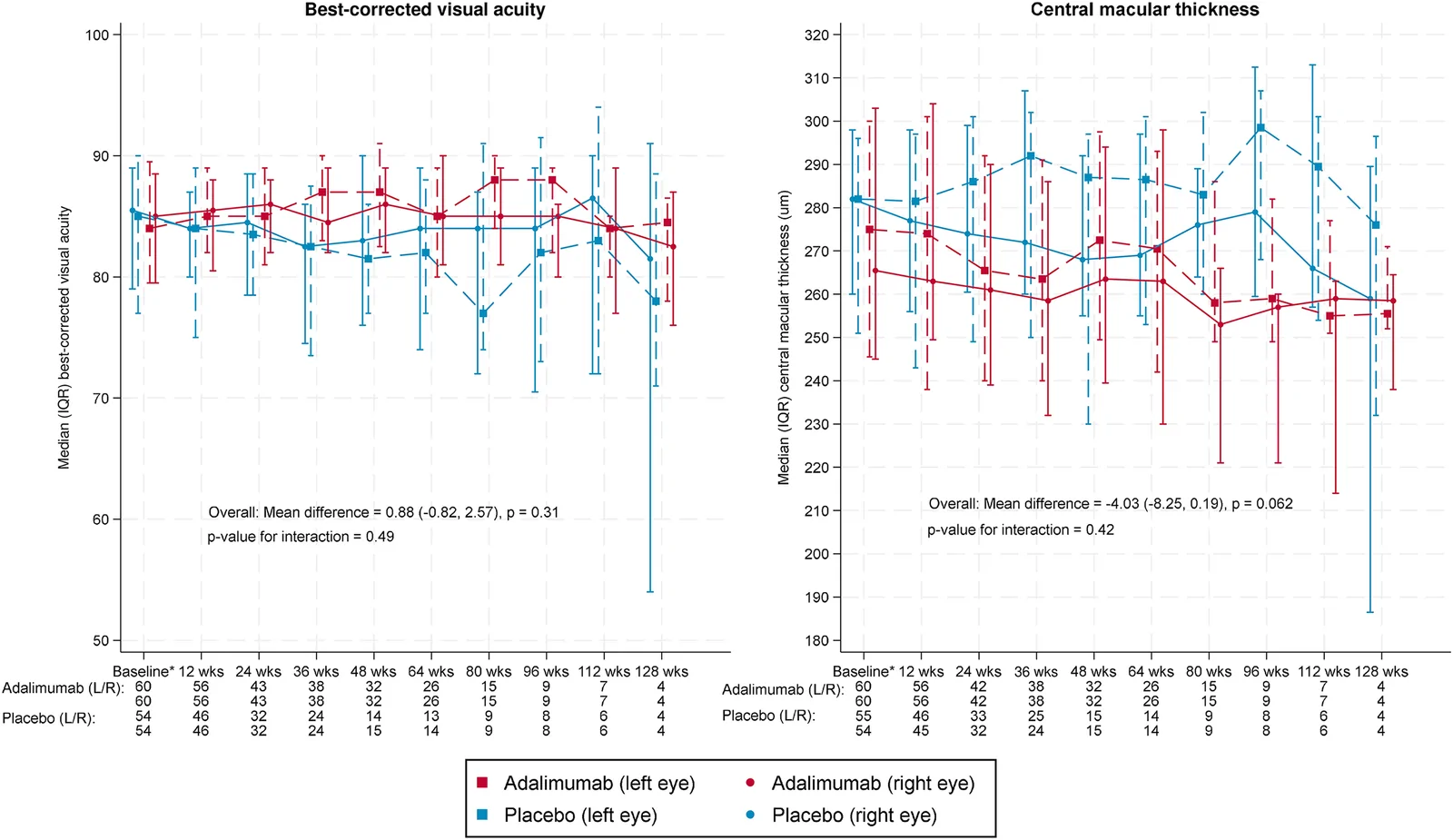
Visual longitudinal outcomes. Median (IQR) best-corrected visual acuity (ETDRS letters) and central macular thickness (μm), by eye and treatment group over time. Points show the median and error bars the interquartile range (IQR). ∗Baseline is the end of the treatment run-in (TRI). For best-corrected visual acuity a higher score indicates better vision; for central macular thickness a higher score indicates a poorer outcome. Treatment effects shown within the panels are the overall mean difference with its 95% confidence interval and p-value; the p-value for interaction tests whether the treatment effect changed over time. CI, confidence interval; ETDRS, Early Treatment of Diabetic Retinopathy Study; IQR, interquartile range.

### Harms

AEs and SAEs are described in Table ST12 (Supplementary File), both for the TRI period (when all participants received adalimumab) and RCT. Just over half of participants experienced at least one AE in the TRI (276 events in 57.4% [116/202] of participants, five of which were serious); there were 356 AEs and 6.5 SAEs per 100 person-years. In the RCT there were 195 AEs, 95 in the adalimumab group (162 per 100 person-years) occurring in 55.9% (33/59) of participants and 100 in the placebo group (234 per 100 person-years) occurring in 66.1% (37/56) of participants. Adverse events in the RCT are reported for the as-treated population; the denominators (adalimumab, n = 59; placebo, n = 56) therefore differ from the numbers randomised (n = 60 and n = 55). Two of these events in two participants (one in each group) were classified as serious. AE rates of the two prespecified categories of events of interest were: 47.9 infections and infestations per 100 patient-years in the adalimumab group versus 73.2 per 100 person-years in the placebo group; 8.5 skin and subcutaneous tissue disorders per 100 patient-years in the adalimumab group versus 11.7 per 100 patient-years in the placebo group. Neither difference was statistically significant, although both effect estimates favour adalimumab (Table ST13). Of the seven SAEs occurring throughout the TRI and RCT, four were deemed possibly, probably or definitely related to the intervention (Table ST14), three in the TRI and one in the RCT (adalimumab group); all of these were expected events listed in the Reference Safety Information. There was no Suspected Unexpected Serious Adverse Reaction (SUSAR). There were no deaths in either group during the trial.

### Cost-effectiveness analysis

From randomisation to the first TF or transfer to OLE, participants randomised to adalimumab incurred £1117 additional costs as compared to participants randomised to placebo (p < 0.001), primarily due to the cost of adalimumab (£899.95). At 12 months, the incremental cost-effectiveness ratio was £2320 per TF avoided, representing the additional cost to prevent one extra TF with adalimumab compared to placebo (Table 2). Costs beyond 12 months (up to 128 weeks where data are available) are presented in the Supplementary File (Table ST15).

**Table 2:** Costs of adalimumab, concomitant medication and adverse events in the adalimumab and placebo groups.

	Randomised to adalimumab (n = 60)	Randomised to placebo (n = 55)	Difference (adalimumab minus placebo)	
	Mean (SE)	Mean (SE)	Mean (SE)	p-value
Intervention (adalimumab/placebo)	£899.95^a^	£0	£899.95^a^	
Monitoring visits and clinical examinations	£687.06 (£75.32)	£505.20 (£80.20)	£181.87 (£95.90)	0.06
Concomitant medication^b^	£61.23 (£22.23)	£18.55 (£23.67)	£42.69 (£28.30)	0.13
Adverse event and serious adverse event management^c^	£83.41 (£44.22)	£92.11 (£47.08)	−£8.70 (£56.30)	0.88
Total costs	£1710.41 (£154.22)	£593.53 (£164.20)	£1116.88 (£196.33)	<0.001

Abbreviations: CI, confidence interval; SE, standard error; TF, treatment failure.

Incremental costs at 12 months (CI)^d^: £662.37 (£226.53–£1141.07).

Cost per TF avoided at 12 months (CI): £2320.39 (−£118.36 to £4759.13).

Notes: All randomised participants were included under the intention-to-treat principle and followed until treatment failure or entry to the open-label extension. p-values for costing analyses were adjusted for sex, baseline age, baseline EQ-5D health utility, and baseline better-seeing eye best-corrected visual acuity. Mean values in each treatment group are presented for the average participant (a woman aged 48 years, with better-seeing eye best-corrected visual acuity of 84 letters and EQ-5D health utility of 0.82).

a SEs are not presented as variations that arose solely from randomisation.

b Concomitant medications included medications prescribed after the randomisation and excluded the over-the-counter medications and medications unrelated to the eye or the trial intervention, as determined by clinician review.

c We cost adverse events related to the eye disorders if they required management beyond regular monitoring visits, and cost adverse events related to other organ system classes, if they were deemed by clinical experts as related to the treatment with adalimumab, or the condition, e.g., uveitis, and the treatment of the condition with other medications.

d To estimate the incremental costs at each time point, costs and time to treatment failure were modelled jointly using gamma-distributed costs and Cox proportional hazards for failure time. We present the incremental costs at 12 months with longitudinal results in Table ST15. Costs per treatment failure avoided were also estimated for 12 months.

## Discussion

In this trial involving patients with NIU, either with active or inactive disease but unable to reduce prednisolone to 5 mg/day or less, treatment with an adalimumab biosimilar significantly reduced the hazard of treatment failure, to a similar extent as previous trials.^13^^,^^14^ Patients currently ineligible for treatment with adalimumab in the UK, encompassing diverse clinical phenotypes, experienced the same benefit as those who were eligible.^15^ This finding supports extending NICE's treatment recommendation beyond patients with active disease and to biosimilars already used in the NHS. The results are consistent with the broader licensed indication for adalimumab to treat “non-infectious intermediate, posterior, and panuveitis in adults and paediatric patients 2 years of age and older” without further qualification. The biosimilar used (SB5) is marketed as Imraldi in the European Union/European Economic Area and the United Kingdom and as Hadlima in the USA; the findings reflect a class effect of adalimumab.^29^^,^^30^

A TRI is unusual outside a trial sponsored by the pharmaceutical industry. The treatment run-in used here—treating all participants before randomising only responders to continue or withdraw—is a form of enriched enrolment, randomised withdrawal (EERW) design. Although EERW is often associated with industry-sponsored analgesic trials, the design has been independently characterised and systematically reviewed by academic, non-commercial investigators, most extensively in chronic pain, where frameworks for its rigorous conduct and reporting have been proposed.^31^^,^^32^ This feature was welcomed by patients during development of the trial design. It ensured that adalimumab was only continued in patients for whom it was demonstrably effective after 16 weeks of treatment. Furthermore, with no existing validated biomarkers to predict treatment response, the TRI provided stopping criteria when continuing adalimumab was not in a participant's best interest. Of those who started the TRI, 59% (119/202) achieved prednisolone ≤5 mg/day with inactive disease, including patients who had an inadequate response to or were intolerant of corticosteroids.

Secondary, longitudinal outcomes are difficult to interpret because participants were not followed in the trial after treatment failure and therefore between-group comparisons used data predominantly from patients in remission. Because treatment failure occurred more frequently and earlier in the placebo group, follow-up was shorter in the placebo arm (median 175 versus 331 days), whereas adherence to the allocated injections exceeded 90% in both groups. Nevertheless, estimates for most outcomes favoured adalimumab and borderline differences were observed for patient-reported visual function and CMT. The adverse event data collected in the trial reassures ophthalmologists that adalimumab is safe for the broader population of patients with NIU who were eligible to participate.

The incremental cost-effectiveness ratio was £2320 per TF avoided, which is the additional cost to prevent one extra TF with adalimumab compared to placebo. When compared against the overall economic burden of TF,^33^ this cost represents good value for money for the NHS since TF usually requires intensive treatment (e.g., additional imaging, high-dose steroids, immunomodulatory therapy, intravitreal injections, frequent monitoring and indirect costs such as productivity loss and informal care). The incremental cost-effectiveness ratio of £2320 per treatment failure avoided is a marginal cost metric and is not directly comparable with cost-of-illness estimates. A recent systematic review of NIU economics reported annual direct medical costs of $16,428–$134,135 (2023 USD) per patient, rising 4.3-fold in those with blindness^34^; these disease-specific figures provide context for the economic relevance of preventing treatment failure but are not a formal benchmark, as they describe heterogeneous populations and healthcare systems rather than UK NHS marginal costs. The forthcoming cost-utility analysis from ASTUTE will provide trial-based estimates of costs, QALYs and cost-effectiveness in this population.

The VISUAL I and II randomised controlled trials demonstrated the efficacy of adalimumab over placebo in intermediate, posterior and pan NIU.^13^^,^^14^ The ASTUTE trial extends their findings by comparison with a pragmatic standard-of-care regimen, allowing clinicians to optimise immunosuppression according to clinical need while tapering corticosteroid to ≤5 mg/day. This trial also included patients with visually consequential clinical manifestations of CMO and a vitritis score of 1+, who were excluded from the VISUAL trials. More recently, the ADVISE trial evaluated adalimumab as a first line treatment for patients with active and recently active disease; adalimumab achieved superior corticosteroid sparing compared to conventional immunosuppression (antimetabolites and calcineurin inhibitors) at 6 months and greater corticosteroid discontinuation at 12 months.^35^ Collectively, these trials support adalimumab as both a first-line alternative to conventional immunosuppression and an effective add-on therapy for patients not adequately controlled on standard treatment. However, the one year treatment failure rate of 30.0% in the adalimumab group emphasises the unmet need for additional systemic treatments for NIU.

The strengths of the study are: randomised allocation, placebo control, masking of research personnel, good adherence, the diversity of specific uveitides among the study population, and integral patient involvement. Recruitment from most English hospitals providing a tertiary uveitis service, together with broad eligibility criteria spanning the range of non-anterior uveitides, supports the representativeness of the cohort for routine UK practice. An important limitation was our failure to achieve the target sample size. This was largely due to the COVID-19 pandemic which delayed starting recruitment; when normal activities were resumed, hospitals had to prioritise back-logs over research activities. The decisions to stop recruitment early, and to transition participants to an OLE phase, were made without reference to the data. We had to stop recruiting to the TRI approximately six months ahead of the placebo expiry date due to the duration of the TRI (16 weeks) and the longest interval between follow-up visits (16 weeks) in the RCT. We were careful to ensure that the date of expiry of the placebo did not unmask participants until the start of the OLE. Adalimumab may have induced side-effects in some participants that inadvertently unmasked them. However, the AE profile was similar across the two groups.

In conclusion, this study simulating real world practice provides new evidence of efficacy and benefit of adalimumab in a wide patient population with uveitis. Adalimumab was successful in reducing treatment failure in patients enrolled with active disease or inactive disease and achieving a corticosteroid dose of <5 mg/day of prednisolone. Adverse effects were similar in both adalimumab and placebo groups and the cost-effectiveness results indicate value for money for the NHS.

## Contributors

SMS, ADD and BCR conceptualised the study. ADD, SMS, LC, SW, AF, TP and BCR acquired funding. SMS, ADD, KP, LC, SW, SM and BCR developed the methodology. ADD, SMS, NAVB, LC, SW, AF, KL, TP, SM and BCR contributed to the investigation. AF led patient and public involvement. SB, LC, KL, RCMB and BCR undertook project administration, and SB provided resources. KP, SB and RCMB supervised the study. ML, KP and TP curated the data. ML, KP, XZ and RK performed the formal analysis; ML, KP and XZ developed the software; and ML, KP and XZ prepared the visualisations. KP, XZ, RK, KL and SM validated the results. KP, XZ and BCR wrote the original draft, and ADD, SMS, NAVB, ML, SB, LC, RK, SW, AF, KL, RCMB, TP and SM reviewed and edited the manuscript. ML and KP directly accessed and verified the underlying trial data, and the writing committee had full access to all the data and took final responsibility for the decision to submit. KP is the guarantor for the dataset. All authors had access to the data reported in the study and read and approved the final version of the manuscript. The ASTUTE study investigators, listed in full in the Supplementary Appendix, identified, recruited and consented participants at the 18 participating sites, delivered the trial intervention, performed the study assessments and collected the clinical and outcome data reported here; they are named as collaborators rather than authors, having contributed to the acquisition of data but not to the drafting or critical revision of the manuscript.

## Data sharing statement

Data will be made available for sharing when the main results of the study have been published. Deidentified anonymised individual patient data will be made available for secondary research, conditional on assurance from the secondary researcher that the proposed use of the data is compliant with the MRC Policy on Data Sharing regarding scientific quality, ethical requirements and value for money. A minimum requirement with respect to scientific quality will be a publicly available pre-specified protocol describing the purpose, methods and analysis of the secondary research, e.g., a protocol for a Cochrane systematic review. Data will be made available through the University of Bristol Research Data Storage Facility. The statistical analysis code used to generate the results will be made available on reasonable request to the corresponding author, under the same conditions as the individual patient data.

## Declaration of interests

ADD declares consulting fees from Roche and AbbVie; payment from Alimera for a lecture; payment from Roche for expert testimony to the European Medicines Agency; and is a co-founder of, and holds stock in, Cirrus Therapeutics.

BCR declares that a portion of his salary up to 31 March 2024, and subsequently consultancy fees, were paid by the NIHR HTA grant to the University of Bristol.

LC declares that a portion of her salary was paid by the NIHR HTA grant that funded this work, and that the Bristol Trials Centre received some NIHR Clinical Trials Unit (CTU) support funding, which ended on 30 September 2023.

NAVB declares a speaker fee from Alimera Sciences (June 2023) and committee membership of the Uveitis National Clinical Study Group.

TP declares a grant from the NIHR HTA, paid to her institution.

SMS declares committee membership of the Uveitis National Clinical Study Group, consulting fees from AbbVie and Johnson & Johnson, and a speaker fee from Alimera.

ML, KP, RCMB, SB, KL, AF, RK, XZ, SW and SM declare no conflicts of interest.

## Appendix A Supplementary data

ASTUTE Protocol

Supplementary Materials

Consortium Investigators

SAP v2.0 signed

ASTUTE Protocol V14.0
