# Supplementary material for: Adalimumab versus placebo as add-on to standard therapy for non-infectious uveitis in the United Kingdom: the ASTUTE randomised controlled trial with treatment run-in
Source: eClinicalMedicine. 2026 Oct 8;101:104240. doi: 10.1016/j.eclinm.2026.104240 (PMC13677017; doi:10.1016/j.eclinm.2026.104240)
Supplement: Supplementary file 1 [file mmc1.pdf]

Bristol Trials Centre
Bristol Medical School
University of Bristol
1-5 Whiteladies Road
Bristol. BS8 1NU

btc-mailbox@bristol.ac.uk

Adalimumab vs placebo as add-on to Standard Therapy for autoimmune Uveitis: Tolerability, Effectiveness and cost-effectiveness: a randomized controlled trial.

The ASTUTE trial

IRAS ID	271051
ISRCTN No	ISRCTN31474800
EUDRACT no.	2020-000754-97
Sponsor no.	OP/2017/6195
Funder no.	16/24/09

This protocol has regard for the Health Research Authority guidance

Details of Sponsor

University Hospitals Bristol and Weston NHS Foundation Trust
R&D Office
Level 3, Education Centre
Upper Maudlin Street
Bristol BS2 8AE

Tel: 0117 342 0233
Fax: 0117 342 0239

Chief Investigator& Research Team Contact Details

Chief Investigator
Professor Andrew Dick
University of Bristol
Tel: 0117 342 4854
Email: a.dick@bristol.ac.uk

Senior Methodologist and co-applicant
Professor Barnaby Reeves
University of Bristol
Tel: 0117 456 0234
Email: barney.reeves@bristol.ac.uk

Co-Lead clinical investigator
Dr Srilakshmi Sharma

Consultant ophthalmologist and co-applicant
Dr Alastair Denniston
University Hospitals Birmingham NHS

Oxford University Hospitals NHS Foundation Trust
Tel: 01865 594 299
Email: srilakshmi.sharma@ouh.nhs.uk

Foundation Trust
Tel: 0121 371 6905
Email: a.denniston@bham.ac.uk

Research fellow and co-applicant
Dr Lucy Culliford
University of Bristol
Tel: 0117 455 3487
Email: lucy.culliford@bristol.ac.uk

Statistician
Dr Katie Pike
University of Bristol
Email: katie.pike@bristol.ac.uk

Consultant ophthalmologist and co-applicant
Mr Carlos Pavesio
Moorfields Eye Hospital NHS Foundation Trust
Tel: 07739 706 258
Email: c.pavesio@nhs.net

Consultant ophthalmologist and co-applicant
Dr Nicholas Beare
Royal Liverpool & Broadgreen University Hospitals NHS Trust
Email: N.Beare@liverpool.ac.uk

Senior Health Economist
Professor Sarah Wordsworth
University of Oxford
Tel: 01865 289 268
Email: sarah.wordsworth@dph.ox.ac.uk

Senior grader, Moorfields reading centre
Dr Pearse Keane
University College London
Tel: 020 7566 2255
Email: pearse.keane1@nhs.net

Public health consultant
Dr Robert Wilson
NHS England
Email: robert.wilson15@nhs.net

Patient and public representative
Ms Annie Folkard
The Birdshot Uveitis Society
Email: annie@birdshot.org.uk

Director of the Central Angiographic Resource Facility, NetwORC UK
Professor Tunde Peto
Queen's University Belfast
Tel: +44 (0)28 9097 6400
Email: t.peto@qub.ac.uk

Table of contents

Glossary / abbreviations **6**
1. Trial summary **8**
1.1 Scientific summary 8
1.2 Plain English summary 9
2. Background **11**
2.1 The problem addressed by the trial 11
2.2 Reasons why this research is important 12
2.3 Evidence that there is a current need for the trial 13
3. Rationale **14**
4. Aims and objectives **15**
5. Plan of Investigation **15**
5.1 Conceptual framework 15
5.2 Trial schema 18
5.3 Trial design 19
5.4 Setting 20
5.5 Key design features to minimise bias 20
5.6 Trial population 20
5.7 Trial interventions 22
5.8 Primary and secondary outcomes 25
5.9 Sample size justification 27
6. Trial methods **27**
6.1 Description of randomisation 27
6.2 Masking and unmasking 27
6.3 Research procedures 28
6.4 Duration of treatment period 29
6.5 Definition of end of trial 29
6.6 Data collection 33
6.7 Source data 36
6.8 Planned recruitment rate 36
6.9 Participant recruitment 36
6.10 Discontinuation/withdrawal of participants 37
6.11 Frequency and duration of follow up 38
6.12 Likely rate of loss to follow-up 38
6.13 Expenses 38
7. Statistical analyses **38**
7.1 Plan of analysis 38
7.2 Subgroup analyses 39
7.3 Frequency of analyses 39
7.4 Criteria for the termination of the trial 39
7.5 Economic issues 39
8. Trial management **40**
8.1 Day-to-day management 41
8.2 Monitoring of sites 41
8.3 Trial Steering Committee and Data Monitoring and Safety Committee 41
9. Safety reporting **41**
9.1 Expected adverse events associated with the study medication 42
9.2 Period for recording serious adverse events 42
10. Ethical considerations **44**

10.1 Review by an NHS Research Ethics Committee 44
10.2 Risks and anticipated benefits 44
10.3 Informing potential study participants of possible benefits and known risks 45
10.4 Obtaining informed consent from participants 45
10.5 Co-enrolment 46
11. Research governance 46
11.1 Sponsor approval 46
11.2 NHS confirmation of capacity and capability 46
11.3 Investigators' responsibilities 46
11.4 Monitoring by sponsor 46
11.5 Indemnity 47
11.6 Clinical Trial Authorisation 47
12. Data protection and participant confidentiality 47
12.1 Data protection 47
12.2 Data handling, storage and sharing 47
13. Dissemination of findings 48
14. References 48
15. Amendments to protocol 52

Glossary / abbreviations

ANIU	Autoimmune non-infectious uveitis
AE	Adverse event - any undesirable event in a subject receiving treatment according to the protocol, including occurrences which are not necessarily caused by or related to administration of the research procedures.
ASTUTE	Adalimumab vs placebo as add-on to Standard Therapy for autoimmune Uveitis: Tolerability, Effectiveness and cost-effectiveness. The ASTUTE pragmatic randomized controlled trial.
BCVA	Best corrected visual acuity
BTC	Bristol Trials Centre
BUS	Birdshot Uveitis Society
CMO	Cystoid macular oedema
CMT	Central macular thickness
CRF	Case report form
CS	Corticosteroid
DMSC	Data monitoring and safety committee
ECG	Graphical representation of electrical activity of the heart over time, as recorded by an electrocardiograph
EQ-5D-5L	EuroQol-5 Dimensions, 5-level version
ETDRS	Early Treatment of Diabetic Retinopathy Study
HIV	Human immunodeficiency virus
HLA	Human leukocyte antigen
HR	Hazard ration
HRQoL	Health-related quality of life
ICF	Informed consent form
ICH-GCP	International conference for harmonisation of good clinical practice
IFR	Individual funding request
IL	Interleukin
IMP	Investigative medicinal product
IMT	Immunomodulatory treatment
MHC	Minor histocompatibility complex
MLHF	Minnesota living with heart failure
MRI	Magnetic resonance imaging
NHS	National Health Service
NIHR	National Institute for Health Research
OCT	Optical coherence tomography
PIL	Patient information leaflet
OLE	Open-label extension
PPI	Patient and public involvement
RCT	Placebo-controlled randomised controlled trial (masked)
REC	Research ethics committee
RSI	Reference safety information
SAE	Serious adverse event - events which result in death, are life threatening, require hospitalisation or prolongation of hospitalisation, result in persistent or significant disability or incapacity.
SAR	Serious adverse reaction
Sciensus	(Formerly called Healthcare at Home Ltd). Third party organisation responsible for distributing trial medication to each participant's home and showing them how to use the pre-filled pens.

SOP	Standard operating procedure
SmPC	Summary of product characteristics
SSAR	Suspected serious adverse reaction
SUSAR	Suspected unexpected serious adverse reaction - an untoward medical occurrence suspected to be related to a medicinal product that is not consistent with the applicable product information and is serious.
TF	Treatment failure
TMG	Trial management group
TNF	Tissue necrosis factor
TRI	Treatment run-in
TSC	Trial steering committee
VCM1	Vision-related quality of life core questionnaire
VFQ	25-item National Eye Institute Visual Functioning Questionnaire
UK	United Kingdom
UHBW	University Hospitals Bristol NHS Foundation Trust
WHO	World Health Organisation
WPAI-SHP	Work Productivity and Activity Impairment Questionnaire: Specific Health Problem V2.0

1. Trial summary

1.1 Scientific summary

Design:	Phase IV pragmatic placebo-controlled, randomised controlled trial (RCT) with a 'treatment run-in' (TRI) and open-label extension (OLE). After the TRI, responders only (inactive disease with <=5mg/day oral prednisolone, estimated 50%) will be randomised (1:1) to adalimumab or placebo. An internal pilot (phase 1; 18 months (m) including 6 months (6m) set up) will determine recruitment and responder rates among eligible patients; progression to phase 2 will depend on a) randomising >=80% (n=41) of the target number at this time (n=51), b) having at least 10 sites recruiting to the trial by month 18 and c) <15% of responders at the end of the TRI unwilling to be randomised.<br><br>The RCT will evaluate the effectiveness and relative cost-effectiveness of adalimumab vs placebo as add-on therapy to standard care.<br><br>When existing placebo stock becomes unavailable due to expiration, participants will be unmasked to their current allocation at their last RCT visit before placebo expiry (31 Jan 2026). If they choose to transition to the OLE, they can continue follow-up until their last visit before end of data collection (31 March 2026).<br><br>Estimates of effectiveness and relative cost-effectiveness (adalimumab vs placebo or no injection) will be updated at the end of the OLE.
Setting:	UK tertiary centres treating autoimmune non-infectious uveitis (ANIU).
Target population:	Adults with active (incident) or inactive (prevalent) ANIU, requiring/starting >5mg/day oral prednisolone to treat disease activity.
Inclusion criteria:	Patients with sight threatening or controlled ANIU in either or both eyes and requiring >5.0 mg oral prednisolone.
Exclusion criteria:	Steroid-dependent systemic disease requiring >5mg/day oral prednisolone, untreated or active tuberculosis, uncontrolled glaucoma, multiple sclerosis, HIV positive, hepatitis B or C, syphilis, Lyme disease, toxoplasmosis chorioretinitis, Behcet's disease, heart failure (NYHA III/IV), cancer diagnosed <5 years ago or monitoring of cancer where oncologist has concern about anti-TNF, anti-TNF drug within 90 days, intra or peri-ocular steroids within a specified timeframe, pregnant, allergy or hypersensitivity to adalimumab or any of its excipients, presence of an epiretinal membrane likely to prevent an eye meeting response criterion at 16 weeks of central macular thickness <320um.
Health technologies	80mg subcutaneous injection of Imraldi^{TM} (licensed biosimilar for adalimumab) at the start of the TRI, then fortnightly 40mg injections starting one week after the initial dose, up to 16 weeks (end of TRI); after randomisation, fortnightly injections of 40mg drug or placebo to the end of the

being assessed:	RCT (for between 12 and 170 weeks, about 3 to 40 months), including repeat TRIs;<br><br>On transition to the OLE, participants on drug in the RCT will continue to receive fortnightly injections of 40mg drug, participants on placebo in the RCT will receive no ongoing treatment until the end of the OLE (for up to another 76 weeks, about 18 months). In the event of TF in the OLE, participants will exit the study.
Measurement of outcomes. Primary outcome:	TF, defined as need for >5mg/day oral prednisolone to maintain inactive uveitis (decisions to increase oral prednisolone made by masked clinicians) or treat active disease (see 5.8.1), [1] excluding isolated anterior uveitis and including an increase in cystoid macular oedema (see flow diagram). Clinical TF events will be validated by retinal imaging. Follow-up will be censored when all participants have at least 12 weeks follow-up in the RCT and up to 76 weeks follow-up in the OLE.
Secondary outcomes include:	Patient reported outcomes (visual function [2], generic [3], and symptoms of side effects), BCVA, individual TF components, retinal morphology, adverse events (AEs), changes in employment, NHS resource use and costs.
Cost-effectiveness analysis:	The main outcome measure for the economic evaluation will be quality adjusted life years (QALYs), estimated using the EuroQol EQ-5D 5L, administered at every visit. Valuations derived from published UK population tariffs will be assigned. The mean number of QALYs per group and incremental QALYs will be calculated.
Sample size:	Hazard ratios (HRs) in 2 trials of adalimumab to treat active and inactive ANIU were 0.50 and 0.57 respectively [1, 4]. The TRI design should improve effectiveness and we have set a target HR of 0.5. Assuming that 27% in the placebo group survive free from TF at 12m (estimated from placebo groups of the 2 trials with 40:60 active: inactive disease), 174 participants will allow a HR=0.5 (27% vs 52% survival free from TF at 12 months) to be detected with 90% power and 5% 2-tailed significance, with <=10% loss to follow-up.
Project timetable:	58 months comprising 7 months set-up; 34 months recruitment (which includes 4 months TRI and 4 months randomising last recruited participants at end of TRI); 14 months follow-up in OLE 3 months data for final analyses and reporting. (Ongoing data checks will be completed.)
Expertise in team:	The multidisciplinary team includes ophthalmologists who treat ANIU, a patient with ANIU, an imaging expert, clinical trials researchers with expertise conducting ophthalmic trials and a health economist.

1.2 Plain English summary

Autoimmune uveitis is a term for several rare eye diseases in which the body's own immune system causes sight-threatening damage to the light sensitive retina at the back of the eye. Uveitis causes sight loss from inflammation inside the eye, damage to blood vessels in the

retina or leakage of fluid into the central, most sensitive area of the retina. Two in 10,000 people are at risk of serious sight loss from uveitis. Usual treatment for autoimmune uveitis involves low dose steroids and one or two other drugs to reduce inflammation. Unfortunately, many patients do not respond to or tolerate usual treatment, or they need high dose steroids to control the uveitis. Long term high dose steroids increase the risk of heart attack, stroke, and infection and affect physical and mental health. Adalimumab is a drug that targets chemicals released by inflamed tissue, neutralising their damage to the body. It was first approved in Europe to treat uveitis in 2016.

Two recent studies suggest fortnightly adalimumab is, on average, an effective way to treat uveitis in some patients. However, drugs like adalimumab can have serious side effects and more evidence is required to identify which patients with uveitis benefit the most from adalimumab, both with respect to their vision and quality of life, including treatment side effects. In 2017, NICE recommended that it should be used in the National Health Service (NHS) but only for a minority of patients with uveitis. Several very similar (biosimilar) drugs are now available, reducing the cost of treatment, and ophthalmologists believe that many more patients could benefit but have no way to identify those most likely to benefit. This study is designed to evaluate one of these biosimilar drugs, now being purchased by the UK NHS for usual care.

This study aims, first, to identify patients who are most likely to benefit from adalimumab. All eligible patients who consent will be given adalimumab for a 16-week trial period, if necessary in combination with low dose of steroids; these patients will include those with impaired vision due to uveitis, requiring high dose steroids to bring the disease under control, and those with better vision but who require high dose steroids to keep the uveitis under control. Over the 16 weeks, doctors will aim to reduce the steroid dose to a low level that should not cause side effects.

Then, patients who are successfully treated with adalimumab and low dose steroids will enter the main study. They will be given adalimumab or an identical-looking dummy treatment (so no one knows which treatment a patient is receiving), in combination with their other medications (including low dose steroids). Chance will determine who receives which treatment and neither patients nor their eye doctors will know. Regular eye examinations, tests and questionnaires will be used to assess how well patients are doing. This part of the study, which will treat and follow up patients until the end of the trial (for between 12 and 170 weeks, about 3 and 40 months), will find out whether adalimumab is better at preventing recurrence of uveitis than the dummy treatment and whether adalimumab is cost-effective compared to the dummy treatment. At the end of the main study, patients will be invited to be followed up for up to another 76 weeks, receiving adalimumab if they have been having adalimumab, or receiving nothing but continue to attend study visits if they have been having the dummy treatment.

Patients with uveitis have contributed to the study from the start, helping to: design the protocol to ensure it applies to uveitis patients who may benefit; co-authoring the lay summary; helping to draft the application, providing feedback on the trial design and participating in a national survey to assess support for the study. They will continue to contribute in these ways and provide support to patients, if funding is awarded. The research team includes eye doctors and researchers with expertise in doing eye studies. A registered clinical trials unit, ophthalmology research networks and patient groups will collaborate to conduct the study. The results will be disseminated through NHS England, patient societies, newsletters to participants and through medical journals.

2. Background

2.1 The problem addressed by the trial

Uveitis is defined as inflammation of the vascular uveal tract of the eye, including the iris, ciliary body, and choroid; however, adjacent structures such as the retina, optic nerve, and sclera may also be affected. Therefore, in practice, any intraocular inflammation involving compromise of the blood ocular barrier is considered to be in the same group of disorders. In 2010, the World Health Organisation (WHO) estimated that 285 million people were visually impaired; of these, 39 million were blind, and approximately 10% was due to uveitis [5]. In the United States and Europe, uveitis accounts for 10-20% of severe visual handicaps, and 10% of blindness, in working age adults [6-10].

Clinically, uveitis is classified anatomically as anterior, intermediate, posterior or panuveitis, depending on which anatomical structures of the eye are involved [11]. All these forms are characterised by a cellular inflammatory infiltrate, which ophthalmologists visualise directly in a clinic setting using a biomicroscope. The anterior chamber of the eye is filled with optically clear aqueous fluid, allowing the practitioner to clearly see infiltrating leukocytes that are counted and scored in accordance with standardized grading systems [11]. This process also applies to vitreous gel, which fills the posterior segment of the eye.

The clinical phenotype of non-infectious intraocular inflammation is replicated in experimental animal models that are driven by immune responses to self antigen [12]. The animal models support a role for autoimmunity, albeit experimentally inflammation is often shaped by the presence of mycobacterial protein. However, unlike other classical systemic autoimmune disorders, there are no clearly defined serological markers to assist diagnosis (e.g. autoantibodies) within the majority of uveitis entities, except high human leukocyte antigen (HLA) association (HLA-A29 and Birdshot chorioretinopathy). Markers are also not predictive of either severity or prognosis.

Idiopathic autoimmunity arises following the activation and expansion of retinal antigen-specific T lymphocytes. Experimentally, the triggering event can happen at sites distant to the affected organ, although whether this occurs in human disease is rarely known. The dominant paradigm is that of a CD4+ T helper cell-driven process. The relevance of this to human disease is supported by the association of sympathetic ophthalmia and Vogt–Koyanagi–Harada disease with specific HLA class II alleles [13, 14], as well as the identification of ocular antigen-responsive T cells in both the peripheral blood and eyes of patients [15, 16]. The strong minor histocompatibility complex (MHC) association with autoimmunity arises both through the need for specific autoantigen presentation [17] and through the selection of a potentially pathogenic T cell repertoire [18].

In animal models, the CD4 T cell paradigm is further refined, with both Th1 and Th17 T helper cells implicated as important inducers of autoimmune disease [19]. When CD4+ T cells were purified from the retinas of animals with uveitis induced by peripheral immunisation, and then studied ex vivo, both Th1 and Th17 cells were found. Cytokines produced by these cells condition the local microenvironment, and activate macrophages (especially IFN-γ produced by Th1 cells) to secrete TNF alpha, recruit neutrophils, and potentially restructure the local environment (e.g., through IL-17 produced from Th17 cells; [20]). Differentiated T cell subpopulations also have a role in controlling local inflammation when they acquire a T regulatory phenotype. The normal ocular microenvironment favours differentiation to Foxp3+ regulatory T cells, but when the eye is already inflamed this is not the case [21]. One of the

most marked consequences of autoimmune T cell ocular responses is the resultant increased TNFα production [22-24]. TNFα production within the eye (infiltrating monocytes, macrophages and retinal pigment epithelium) critically mediates retinal tissue damage in uveitis by influencing mononuclear cell trafficking into the retina and initiation of a cascade of inflammatory pathways resulting in direct retinal tissue injury [25]. The central role of TNFα in relation to severity is evidenced by the degree of improvement of uveitis and limitation of tissue destruction following TNFα inhibition [25]. In humans, soluble TNFα is increased in paired serum and aqueous humour with uveitis and is reduced upon remission [26-29].

In summary, the pathophysiology of uveitis is underpinned by autoimmune pathophysiology: (a) central tolerance; (b) recognition of cognate retinal antigens by T helper (Th1 and Th17) cells; and (c) increased ocular production of the cytokine, TNF alpha which perpetuates chronic inflammation, causing end-organ ocular tissue damage which results in blindness.
Unlike other rare diseases treated with biologic drugs, such as associated vasculitides or pulmonary immune-mediated diseases, there is less clear evidence for a dominant, single therapeutic target causing tissue damage. For uveitis, much knowledge has accrued through interrogating immunopathological processes in animal models of uveitis (see above), with results that eloquently illuminate specific targets. Overall, the models have demonstrated a pivotal role for TNF-alpha, as well as activated CD4 T cells, their signature cytokines, and their ability to influence trafficking of cells. This mechanism has been substantiated by the recent findings from the VISUAL trials (NCT01138657; NCT01124838) [1, 4].

2.2 Reasons why this research is important

ANIU affects 115.3/100,000 patients; the incidence is 52.4/100,000 [30]. Despite its rarity, ANIU is the third leading cause of blindness in the western world [5]. For about 20% of patients with sight-threatening ANIU, usual care is immunomodulatory treatment (IMT) to maintain remission with low dose or no corticosteroids (CS) [31, 32]. However, usual care fails for 40% of patients with sight-threatening ANIU [33-35]. Due to lack of alternative therapies, they are treated with long term high dose CS to maintain disease remission and prevent sight loss but at the cost of significant morbidity [36, 37]. Patients may also be intolerant of high levels of one or more conventional IMTs.

Adalimumab is licensed to treat ANIU refractory to CS or if CS is contraindicated. We estimate that 3800 patients in England do not respond to usual care, which includes IMT. IMT may be effective, well tolerated and used in conjunction with adalimumab to achieve low dose CS when IMT alone is not successful. There are no randomised controlled trials specifically evaluating adalimumab as an add-on therapy. This research will collect patient reported outcome measures in association with clinical measures and conduct a cost-effectiveness analysis alongside the trial, which is essential for commissioning.

Sight loss in ANIU is due to cystoid macular oedema (CMO), vitritis, retinal hypoperfusion, retinal scarring or ocular complications [5, 38]. Patients are affected in several ways: sight loss impairs activities that depend on vision (e.g. driving) and, combined with drug side effects, reduces health-related quality of life (HRQoL), increasing the risk of depression [39-41]. Also, 70-90% of ANIU patients are of working age (20-60 years), 43% of them at risk of losing, or having lost, their jobs [42]. CS have serious morbidity and mortality risks; >7.5mg/day prednisolone increases the risk of heart attack, stroke, other systemic and ocular comorbidities [37, 43, 44]. In one UK cross–sectional study, 61% of ANIU patients were taking 40mg/day CS [45]. Published patient reported outcome measures developed for patients with visual loss and validated for uveitis show that visual loss in one or both eyes in uveitis are associated with

significant impairment in psychological well-being, social activities, visual function such as walking down stairs or driving, dependence including being forced to stay at home most of the time while improvement in vision is correlated with an improvement in visual quality of life scores [40, 41, 46]. Poorer visual function, and current oral corticosteroid use, are associated with depression in people with ANIU [39].

Patient surveys conducted by the patient groups developing this trial with the trial organisers (Birdshot Uveitis Society (BUS)) found that, after visual loss, co-morbidities due to treatments for uveitis, in particular steroids, are the greatest cause of poor health, well-being and quality of life. We conducted a patient panel and a nationwide survey of patients with uveitis to establish views about the possible role of biologic therapies. There was unanimous agreement from patients surveyed and panel members for a trial evaluating adalimumab as add-on therapy and agreement that the proposed trial as designed is both equitable and acceptable.

2.3 Evidence that there is a current need for the trial

The importance of ANIU in relation to the commissioning brief is evidenced by a response to a Freedom of Information request by the research team before applying for funding (10/08/2016). The response stated that 50% of all individual funding requests (IFRs) to NHS England for biologic therapy for autoimmune disease from 01/04/2013 to 31/03/2016 were for uveitis. Most will have been for anti-TNFalpha drugs such as adalimumab, which is effective for ANIU [1, 4].

Although there were frequent IFRs at the time of designing the study, there was no commissioning policy for adalimumab in England for adults. Adalimumab cost £11,000/year when first licensed [47] but the availability of biosimilars have recently reduced this to about £6,600/year (60%), a significant cost-saving to the public if this therapy is commissioned. The cost of sight loss from any cause in 2008 was £17,549 and the cost of ANIU complications is high [37, 48]. This research is needed to provide evidence that adalimumab is effective as an add-on therapy to existing safe doses of immunosuppression, to identify disease phenotypes which may respond, and to produce commissioning guidelines for this high cost therapy.

Systematic reviews of anti-TNFa drugs conclude that adalimumab is beneficial for ANIU refractory to IMT [49, 50]. There is evidence from studies other than randomised controlled trials of the benefit of anti-TNFa drugs in small numbers of patients with specific ANIU syndromes, e.g. multifocal choroiditis, punctate inner choroidopathy and Birdshot Uveitis [51]. Unlike other rare diseases treated with biologics, there is strong evidence that TNFa is a major therapeutic target, increasing the likelihood of anti TNFa drugs being effective for ANIU [30]. Two recent multicentre randomised controlled trials reported in 2016 that, compared to placebo, adalimumab delays treatment failure (TF) for active and inactive ANIU (respectively, VISUAL 1, hazard ratio (HR)=0.5; 95% CI, 0.36-0.70; $p<0.001$ and VISUAL II, HR=0·57, 95% CI 0·39-0·84; p=0·004) [1, 4].

The applicability of these results to patients in the United Kingdom (UK) with refractory ANIU is uncertain. The trials did not study adalimumab as add-on therapy and the most important sight-threatening complication of uveitis, CMO, was not an eligibility criterion or a TF event. Furthermore, the presence of inflammation in the front part of the eye (anterior chamber) was considered a failure, whereas in routine clinical practice, it is acceptable to attempt to maintain remission by using adjunctive topical corticosteroid use which is effective and safe for most patients. The trial design for ASTUTE allows topical corticosteroid therapy.

3. Rationale

The trial arises because of the views of ophthalmologists that adalimumab is effective in a wider range of patients with ANIU than covered by the technology appraisal published by NICE [52]. Adalimumab is also ineffective in a proportion of patients covered by the technology appraisal [1, 4]. The design of the trial sought to include features to promote its acceptability to patients affected by uveitis and representatives of organisations representing them. These features are:

A. The offer of a period of treatment with adalimumab to any patient meeting the eligibility criteria for the trial (wider ranging than the criteria published by NICE [52]), i.e. to satisfy the need for the trial to be equitable, with subsequent participation in the randomized phase of the trial being contingent on demonstration of a therapeutic response to the drug.
B. The use of a biosimilar approved by the NHS.
C. In the event of treatment failing in the randomized phase of the trial, the offer to switch treatment to the alternative group without unmasking, so that a participant originally allocated to placebo who relapses could then receive treatment (albeit still masked to allocation).
D. The application of objective clinical criteria to assess whether a therapeutic response has been achieved at the end of the treatment run-in phase of the trial.

To the extent that the trial has been designed to model a future commissioning policy, it also addresses a NHS priority, namely obtaining better evidence to guide effective provision of expensive biologic treatments.

The acceptability of the design features of the trial to patients affected by uveitis was discussed at a patient and public involvement (PPI) meeting with patients and representatives of the Birdshot Uveitis Society and Fight for Sight. The meeting was chaired by Annie Folkard, a co-author on this protocol. The meeting was supported by a subsequent poll of members of the Birdshot Uveitis Society. Conclusions from these consultations were that:

- The trial was considered to be equitable and the broad eligibility criteria were welcomed.
- A minority (<20%) expressed some or strong concern about using a biosimilar approved by the NHS for treating patients with ANIU, because they were of the view that the biosimilar would not be identical to the original drug.
- The offer of switching to the alternative treatment in the event of treatment failure was appreciated; up to two switches was considered optimal.
- Patients would be likely to respect the decisions about patients' therapeutic responses made on the basis of objective clinical criteria at the end of the treatment run-in period.
- The duration of participation in the study was not considered a negative feature.

4. Aims and objectives

The ASTUTE pragmatic trial will compare the effectiveness and cost-effectiveness of adalimumab when used to treat ANIU in either or both eyes in patients taking less than or equal to 5mg of steroids per day and other IMT drugs, as required. Specifically, we will test the hypothesis that adalimumab reduces the hazard of TF in patients with ANIU, after weaning of oral prednisolone to <=5mg/day in a treatment run-in period (TRI).

Specific objectives are:

A. To estimate the hazard of TF in the group allocated to adalimumab compared with the group allocated to placebo.

B. To estimate differences between groups with respect to a range of secondary outcomes including: separate components of the composite TF outcome, visual function, BCVA, ocular and retinal signs of disease activity, health-related quality of life (HRQoL), resource use and costs, adverse events (AEs).

C. To estimate the cost-effectiveness of adalimumab compared to usual care.

D. To investigate associations between patient factors and ocular and retinal signs at the start of the TRI with responder status at 16 weeks, and to explore emerging participant phenotypes associated with TF during follow-up in the main trial.

5. Plan of Investigation

5.1 Conceptual framework

The trial needs to be placebo-controlled because participants will be administering injections themselves and reporting important secondary outcomes, e.g. AEs and the impact of ANIU (which we hypothesise will be effectively treated by adalimumab) on health and employment (including the EQ-5D 5L questionnaire, which will provide the primary outcome for the economic evaluation); these outcomes are likely to be biased if participants know their allocations. The most secure way to mask allocation is to dispense placebo in pre-filled pens that are indistinguishable to the pre-filled pens containing active drug. The manufacturer of the biosimilar used in the trial (Biogen Inc) is supplying placebo pre-filled pens for the trial which are identical to the pre-filled pens containing the licensed adalimumab biosimilar (Imraldi™) which Biogen also manufactures. The only difference is that the Imraldi™ pre-filled pens will have a commercial label and the placebo pre-filled pens will be supplied without the commercial label. The Imraldi™ and placebo pre-filled pens will therefore be over-labelled, with both described only as investigative medicinal product (IMP) to mask the allocation.

An important requirement of the funder was that the trial design should be “both efficient and most likely to provide an answer for a broad range of patients.” To satisfy this requirement, we have specified (a) a more inclusive study population than recruited to previous trials of adalimumab for treating ANIU [1, 4] and (b) included a TRI, with subsequent randomization being contingent on a participant having a therapeutic response. The more inclusive population reflects the population which ophthalmologists want to treat and which they believe is likely to benefit (on average). The TRI is intended as a model for a potential commissioning strategy. It provides an opportunity for all patients in a more inclusive patient population to access treatment, avoiding difficult decisions about eligibility against stricter eligibility criteria and reassuring patients about equitable access, while limiting longer term treatment to only those patients who show a favourable response in the short term (TRI phase). In effect, we are using

short term responsiveness to “personalise” treatment, given that there are no proven biomarkers for responsiveness to adalimumab in patients with ANIU. If objective D (see paragraph 4 above) identifies clear markers for responder status or TF, this would allow eligibility for an ‘initial treatment trial’ in a commissioning policy to be modified.

Ophthalmological trials usually follow participants for a set duration after randomisation. However, this approach poses a dilemma about how to treat the first randomised participants when they reach the end of their follow-up, long before the end of the trial when the results will be known. This challenge is especially acute if follow-up stops when a participant experiences the primary outcome. When evaluating an intervention that is not available outside a trial, this means either stopping an intervention that may be effective or providing open label treatment outside the trial. Both approaches risk anecdotal information about the effects of treatment (from the usual care follow-up of participants who have completed follow-up) becoming available before the trial reports, potentially biasing trial personnel. Withdrawing treatment that may be effective from participants at the end of follow-up may also be considered unethical and inappropriate when participants have volunteered to contribute to the evidence base for the intervention. To avoid this dilemma, we intend to try to keep participants in the placebo-controlled masked randomised controlled trial (RCT), including repeat TRIs, until the trial will be closed to reporting, a maximum of 170 weeks (40 months). After this, participants can choose to participate in an open-label extension (OLE) for up to a maximum of 76 weeks (18 months) [53]. This approach is consistent with how adalimumab is used in practice in patients who are maintained on it successfully, so will contribute valuable longer-term evidence about the intervention, but it requires more IMP and open-label adalimumab. It is likely to foster better commitment among participants and was supported by the PPI consultation.

Keeping participants in the RCT and OLE until the study will be closed to reporting requires a strategy for managing repeated TFs. In the event of a TF in either eye during the RCT, a participant will stop taking the IMP and re-start open label treatment with adalimumab. In effect, this is the same as re-entering the protocol for the TRI (with other medications, including oral prednisolone being prescribed at the discretion of the treating ophthalmologist). If the participant again meets the criteria for being a “responder” at 16 weeks, he/she will re-start trial follow-up (RCT2) but switch to the alternative IMP to the one provided at randomisation. If the participant does not meet the criteria for “responder” at 16 weeks, he/she will be withdrawn from the trial, without unmasking allocation. Consistent with the PPI consultation, a maximum of two switches will be allowed, i.e. adalimumab-placebo-adalimumab or placebo-adalimumab-placebo. In the event of a TF in either eye during the OLE, a participant will exit the trial (adalimumab is available in usual care for patients with active disease).

Although the switch feature may appear complicated, it has important advantages. It was considered equitable and welcomed during the PPI consultation because it ensures that a participant (classified as a “responder” at the end of the TRI) who is allocated to placebo in the RCT and suffers TF has a chance to receive active drug. We anticipate that the opportunity to receive the opposite IMP in the event of TF will encourage a participant to accept: (a) initial randomisation, (b) that adalimumab is “truly not for them” in the event of multiple TFs and (c) not disclosing allocation until the trial reports, even after withdrawal. It has elements of a cross-over design (considered impracticable because of the long time-course of remission and TF, and controlling disease activity in the event of TF) so provides extra information in a (non-random) subset of participants about outcome with both adalimumab and placebo.

An OLE has been added to the trial design because the placebo pens provided at the start of the trial have an expiry date before the original proposed end of the study, due to the slower than expected recruitment. The primary analysis of effectiveness will be restricted to the masked RCT. However, the OLE provides the opportunity to observe additional TFs over the extended follow-up period and increase the precision of updated effectiveness estimates (see section 7), albeit with a risk of bias in the measurement of outcomes.

Participants who wish to take part in the OLE will give written informed consent. They will be unmasked at their final RCT visit before 31st January 2025. If TF is observed at this visit, irrespective of which TRI this occurs in, the participant will exit the trial. For the OLE, irrespective of the number of switches, those on adalimumab at the time of unmasking will receive open-label drug and those on placebo will receive no treatment but still attend study visits. During the OLE, the repeat TRI and switch feature will not apply; any participant experiencing TF during the OLE will exit the trial. Follow-up for treatment failure (and other outcomes) in the OLE will continue.

5.2 Trial schema

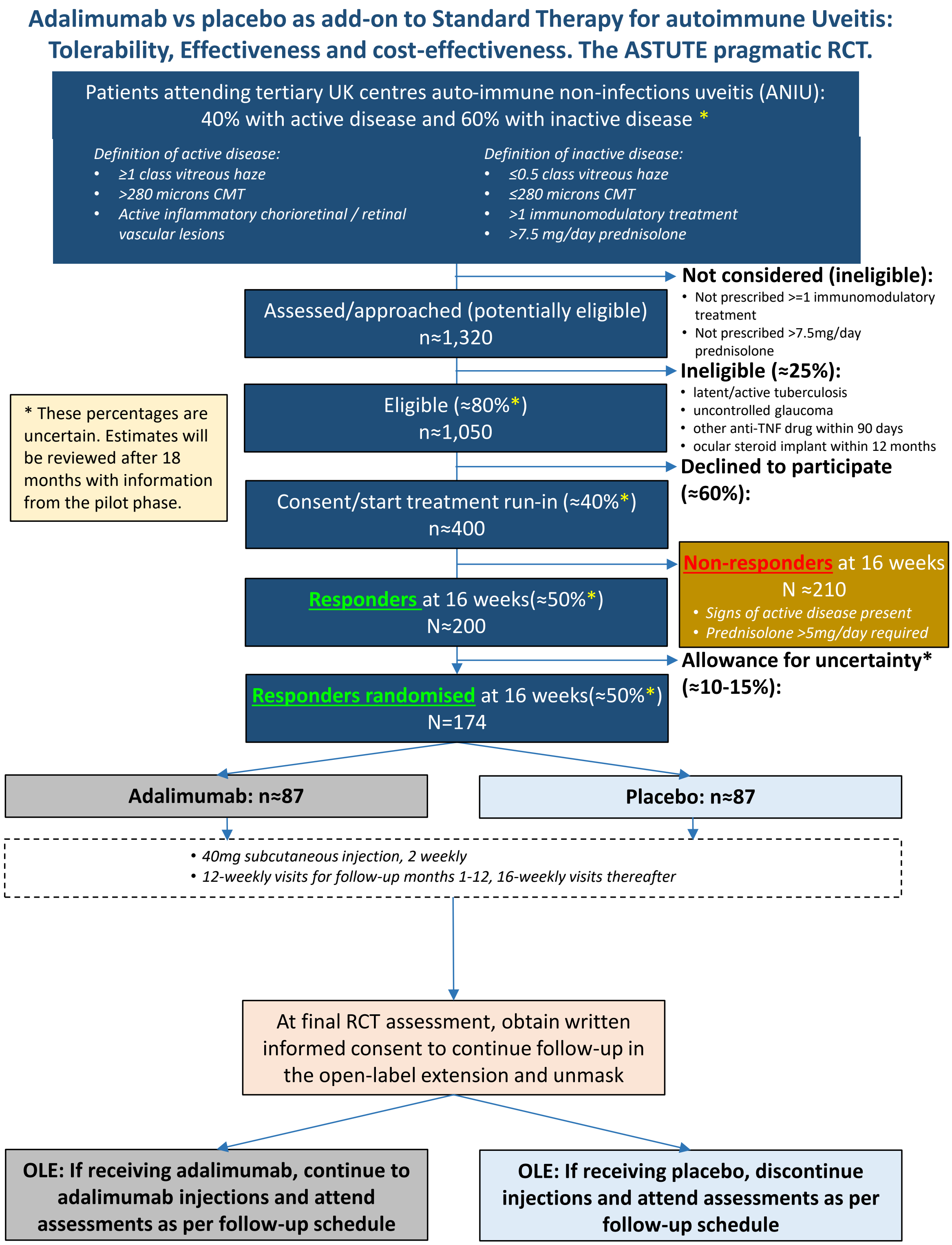

Figure 1: Trial Schema

OUTCOMES

Assess for treatment failure (primary outcome) at each visit (any of the following [objective source]):

A. >=15 letter decrease in best corrected visual acuity (BCVA), cf. average BCVA at 8 and 16 weeks during treatment run-in (TRI) [masked optometric assessment].
B. new active inflammatory chorioretinal lesions [masked clinician grading of fundus colour and autofluorescence images during RCT; unmasked in OLE]
C. >20% increase in central macular thickness (CMT), cf. average CMT at 8 and 16 weeks during TRI [CMT is an automated OCT parameter].
D. onset or worsening of retinal vasculitis [masked clinician assessment of fundus fluoroscein angiogram during RCT; unmasked in OLE]
E. 2 step worsening of vitreous haze cf. best vitreous haze category at 8 or 16 weeks during TRI [masked clinician assessment]
F. prescription of >5mg/day prednisolone to maintain disease remission (i.e. to avert relapse before any criterion for active disease (A-E) is met) [masked clinician decision]

Assess secondary outcome at start of treatment run-in, randomisation, every 6 months after randomisation *and* if participant experiences treatment failure.

If participant experiences treatment failure, follow-up continues with adalimumab/placebo allocation swapped without unmasking allocation.

Serum at start of treatment run-in, randomisation, every 6 months after randomisation *and* if participant experiences treatment failure, and retinal images taken as part of usual care throughout the study, will be collected and stored as a resource for future studies of markers for (a) responsiveness to adalimumab and (b) treatment failure.

Attrition of ≤10% has been taken into account when estimating the target sample size

Figure 2 (continued): Trial Schema

5.3 Trial design

The study design is a pragmatic, multi-centre, parallel group, placebo-controlled, randomised controlled trial (RCT) with a TRI and OLE; only those participants who are classified as “responders” (see 5.1) after 16 weeks of active open label treatment during the TRI will be randomised. During the RCT, participants, doctors and all members of the research team except for the study statistician will be masked to allocation; as participants transition to the OLE, they will be unmasked at their final RCT visit. The study will include three phases: an internal pilot, primarily to establish that recruitment to time and target is possible, the RCT and the OLE. Progression to phase 2 will depend on: a) having randomised >=80% (n=41) of the target number at the end of the internal pilot (n=51); b) having at least 10 sites recruiting to the trial by month 18; and c) <15% of responders at the end of the TRI unwilling to be randomised. The RCT will evaluate the effectiveness and relative cost-effectiveness of adalimumab vs placebo as add-on therapy to standard care. Participants who consent will transition into the OLE at their last assessment in the RCT. The primary effect estimates from the RCT will subsequently be updated with data from the OLE.

Participants starting the TRI will be reviewed at 4-5 weeks, 8-9 weeks (by telephone only) and 16-17 weeks after starting adalimumab treatment. Participants classified as responders (see 6.3.1) who agree to continue in the RCT, will be randomized at the 16-17 week visit (see 6.1). Follow-up visits will be scheduled at 12 weekly intervals (11 to 13 weeks) up to 48 weeks and 16 weekly intervals (15 to 17 weeks) thereafter, consistent with usual care for patients with

ANIU. As participants transition to the OLE, they will remain on the same visit schedule as during the RCT until the end of follow-up (31 March 2026).

<u>Visit/review windows</u>

Treatment run-in weeks 4, 8 and 16: +7 days

RCT all visits: -14 days, +7 days

OLE all visits: -14 days, +7 days

5.4 Setting

This study will take place across NHS tertiary UK ophthalmology departments, which have specialist uveitis services.

5.5 Key design features to minimise bias

The placebo-controlled trial design (including concealed randomisation) will protect against bias arising from the randomisation process. In the RCT, it will also protect against bias due to deviations from intended interventions and bias in measurement of outcomes, since participants, clinicians and other staff caring for participants, members of the research team (except the study statistician) and participants will be masked to participants' allocations [54]. The success of masking will be assessed (participants and their care team may become unmasked due to side effects). These protections will not apply during the OLE.

The allocation will be stratified by centre to minimise confounding due to centre-specific factors. Applying standard protocols, pre-defining procedures for participant follow-up/data collection and applying the procedures to all participants in the same way will help to minimise bias due to deviations from intended interventions and bias in measurement of outcomes in the event of instances of unmasking. Adherence to all aspects of the protocol will be monitored.

Bias due to missing outcome data, i.e. systematic differences in withdrawals between the groups, will be minimised by: i) the offer of a switch in allocation in the event of TF; ii) maintaining regular contact with participants throughout the duration of the trial to maximise the proportion of participants for whom all outcome data are available. The Data Monitoring and Safety Committee (DMSC) will monitor attrition by group.

We will minimise bias in selection of the reported result by pre-specifying outcomes (see 5.8) and by writing a detailed statistical analysis plan in advance of locking the database at the end of data collection (see 7.1).

5.6 Trial population

The target population is adults with active or controlled ANIU. We will define ANIU in relation to anatomic descriptors of intermediate, posterior and panuveitis, because these features cause most sight loss [11, 45]. This will allow treatment results to be applied across specific diseases, increasing applicability to UK patients with ANIU who do not respond to usual care [1, 4, 55, 56]. There is a potential trade-off with efficacy, but it is not feasible to recruit to separate trials for different autoimmune diseases and the TRI design should offset any average loss of efficacy.

The frequency of different specific diseases in the trial population is expected to be similar to other trials: ocular autoimmune (idiopathic) 60%; Birdshot Uveitis 30%; multifocal choroiditis 4%;

PIC 5%; Vogt Kayanagi Harada 1% [4]; and sarcoidosis where the mechanism is autoimmune and TNFa is a critical cytokine.

To inform a commissioning policy for all patients in whom add-on adalimumab is indicated, we need to recruit patients to the TRI who:

(a) present with active sight-threatening ANIU in either or both eyes and who are taking oral prednisolone >5mg/day or being started on >5mg/day, or
(b) patients already being treated for ANIU and taking >5mg/day of oral prednisolone and other IMT drugs as required.

In the subgroup presenting with new active disease, remission will be induced with high dose oral prednisolone; oral prednisolone will be tapered to <=5mg/day over 16 weeks in both groups. The treatment goal in the TRI is the same for both groups, i.e. disease remission with <=5mg/day oral prednisolone (see 6.3.1). If achieved by the end of the TRI, participants will be eligible for randomisation.

5.6.1 Inclusion criteria

A participant may take part in the study if **ALL** of the following apply:

1. Participant is aged 18 years or over;
2. Participant has: (a) active sight threatening ANIU (active inflammatory chorioretinal lesions OR abnormal central macular thickness (CMT) [11] OR evidence of retinal vasculitis [11] OR vitreous haze >0.5) and is being prescribed (already taking or being started on, if newly presenting with ANIU) oral prednisolone >5.0mg/day; OR (b) has controlled ANIU and is being prescribed oral prednisolone >5.0mg/day.
3. Women must have a negative pregnancy test and be willing to use effective contraception* for the duration of the participation in the trial and for 5 months after, or be surgically sterile or post-menopausal for >12 months.
4. Participant is able to provide informed consent.

* This includes: progestogen-only oral hormonal contraception, where inhibition of ovulation is not the primary mode of action, male or female condom with or without spermicide cap, diaphragm or sponge with spermicide, combined (oestrogen and progestogen containing) hormonal contraception associated with inhibition of ovulation (oral, intravaginal or transdermal), progestogen-only hormonal contraception associated with inhibition of ovulation: (oral, injectable, implantable), intrauterine device, intrauterine hormone-releasing system, bilateral tubal occlusion, vasectomised partner, sexual abstinence.
NB. A pregnancy test only needs to be repeated if there is reason to suspect the participant has become pregnant. If a participant becomes pregnant during the trial the treating clinician must discuss the options with the patient on whether or not to continue in the trial, referring to the current SmPC for guidance on pregnancy and Imraldi™.
There are no special precautions/contraceptive requirements for male participants with female partners of child-bearing potential.

5.6.2 Exclusion criteria

A participant may not enter the study if any of the following apply:

1. Participant has controlled ANIU and is maintained on oral prednisolone ≤5.0mg/day at the time of screening;
2. Participant has systemic disease (whether associated with ANIU or not) that is being treated with steroids and requires >5mg/day oral prednisolone.

3. Participant has untreated or active tuberculosis;
4. Participant has severe infection, sepsis or opportunistic infection;
5. Participant has uncontrolled glaucoma;
6. Participant has multiple sclerosis;
7. Participant is HIV positive;
8. Participant has hepatitis B or hepatitis C;
9. Participant has syphilis;
10. Participant has Lyme disease;
11. Participant has Behcet's disease;
12. Participant has toxoplasmosis chorioretinitis
13. Participant has heart failure (NYHA III/IV);
14. Participant has been diagnosed with cancer <5 years ago;
15. Participant is undergoing monitoring for recurrence of cancer / tumour growth where their oncologist has concern that a TNFalpha inhibitor would be contraindicated;
16. Participant is taking another biologic drug;
17. Participant has taken an anti-TNF drug within the previous 90 days (anakinra and abatacept are contraindicated);
18. Participant has had an Iluvien® implant within the previous 18 months and has controlled ANIU, or has had an Iluvien® implant within the previous 12 weeks regardless of whether ANIU is active or controlled;
19. Participant has had an Ozurdex® implant, or an intravitreal steroid injection, or periocular steroid within the previous 12 weeks regardless of whether ANIU is active or controlled;
20. Participant is pregnant;
21. Participant has a known allergy or hypersensitivity to adalimumab or any of its excipients (refer to summary of product characteristics (SmPC) for list of excipients; Appendix 1 section 6.1);
22. Participant is taking part in another interventional study;
23. Participant has an epiretinal membrane likely to prevent an eye meeting response criterion at 16 weeks of central macular thickness <320um.

5.7 Trial interventions

The technology being evaluated is fortnightly 40mg subcutaneous injection of Imraldi™, a licensed biosimilar for adalimumab.

In the RCT, the comparator intervention will be subcutaneous injections of placebo. The placebo for the trial will be provided by Biogen GmbH. It will be identical in all respects to the commercially-available Imraldi™ product, with the exception of adalimumab content and the commercial Imraldi™ label will not be applied. It will be over-labelled to identify the placebo as IMP (see section 5.1). In the OLE, the comparator will be no treatment.

Treatment run-in

The TRI feature of the study will evaluate a policy of treating all eligible patients with adalimumab for 16 weeks but only continuing treatment beyond 16 weeks in the subset that are successfully maintained with inactive disease in both eyes on <=5mg oral prednisolone at 16 weeks.

In accordance with the marketing authorisation for adalimumab, the TRI will commence with an 80mg dose of adalimumab at week 0 using two pre-filled pens under the supervision of a Sciensus Nurse.

Dosing will continue with fortnightly 40mg injections starting one week after the initial dose at weeks 1, 3, 5, 7, 9, 11, 13 and 15 (eight doses in total).

The TRI will end at 16 weeks when response will be assessed. Responders (see 6.3.1) who remain eligible and confirm consent at 16 weeks will be randomly allocated to adalimumab or placebo.

The visit window for the TRI response assessment visit is +7 days only.

Randomised trial (RCT)

RCT dosing will commence 1 week after randomisation, with fortnightly 40mg injections of drug or placebo until the end of the trial (follow-up for between 12 weeks and 170 weeks, about 3 and 40 months).

In the RCT, in the event of TF in either or both eyes, the participant will be offered the opportunity to repeat the TRI. If TF is observed at the final assessment in the RCT, the participant will exit the trial.

The visit window for RCT follow up visits is -14 days to +7 days.

Repeat TRI

TRI dosing will commence 1 week after the date of the response assessment visit , with fortnightly 40mg injections of open label adalimumab for 16-weeks (8 injections in total).

At the end of the 16-weeks the participant's response will be assessed again. If the participant shows a response to treatment as per section 6.3.1, they can re-enter the RCT. If they do not respond, then they will exit the trial.

The visit window for the TRI response assessment visit is +7 days only.

Repeat RCT

Responders will re-enter the RCT and their allocation will be switched in relation to their last allocation (i.e. in RCT1 if entering RCT2, or RCT2 if entering RCT3), maintaining masking (see section 5.1).

Dosing will commence 1 week after the date of the response assessment visit and continue with fortnightly 40mg injections of drug or placebo to the end of the trial.

The visit window for RCT follow up visits is -14 days to +7 days.

In the event of TF in either or both eyes, participants will be offered the opportunity to repeat the TRI for a third time, repeating the above process (if classified as a responder at the end of the TRI, then entering RCT3). Participants can restart the TRI and subsequently switch RCT allocation twice.

OLE

Participants who consent to OLE at their last RCT before 31st January 2025.

For the OLE, irrespective of the number of switches, those on adalimumab at the time of unmasking will receive open-label drug. Dosing will commence 1 week after the date of the response assessment at the final RCT visit and continue with fortnightly 40mg injections of open label drug until they either experience TF or they reach the end of the trial (last patient visit that occurs before 31st March 2026).

Those on placebo will receive no treatment but still attend study visits.

The visit window for OLE follow up visits is -14 days to +7 days. Participants will continue to follow the visit schedule started in the RCT.

Details of the drug are described in the summary of product characteristics (SmPC; Appendix 1) for Imraldi™. The manufacture, quality assurance, labelling and packaging of the placebo are described in the IMP dossier.

Missed injection date

If an injection date is missed, the advice to patients is to administer the missed injection immediately upon remembering. The patient should then adhere to their original injection schedule, i.e. administer their next injection 2 weeks after the date their previous injection was due had they not taken a dose late.

Delayed doses can be taken at any point up until 2 days before the next one is due (i.e., doses cannot be on consecutive days).

Intraocular surgery

Participants who experience TF within 14 days of having intraocular surgery (day of surgery is day 0) during the RCT should continue in the RCT and OLE as if the TF had not occurred (because the TF is assumed to be related to the surgery). These participants should continue to administer the IMP and be followed up at their next scheduled visit. Clinicians can prescribe a single course of oral steroids or intravenous methylprednisolone (maximum of 8-week course) peri or post operatively as would be prescribed in usual care to prevent relapse. Ozurdex can also be prescribed as would be used in usual care. Any additional medication required as treatment for the surgery will be documented on the medication CRF.

If TF criterion or criteria continue(s) beyond 14 days (except >5mg/day oral prednisolone to prevent relapse due to surgery) then true TF has occurred and the participant should restart the TRI.

If participants undergo intraocular surgery during the initial or repeat TRI they will exit the TRI and re-enter when their condition has stabilised after surgery. Intraocular surgery should be reported as an adverse event.

In accordance with standard care for adalimumab, if a patient acquires an infection or serious illness during the trial, the treating clinician may advise the patient to temporarily cease the study medication until it is considered safe to resume administration.

Storage & dispensing

Study medication will be stored by a central NHS pharmacy department in accordance with Good Clinical Practice and pharmacy department SOPs. Study medication will be transferred from the central NHS pharmacy to a third party organisation (Sciensus, formerly called

Healthcare at Home Ltd) to dispense the medication and distribute it directly to participants' homes.

Accountability
Drug accountability including stock control, ordering, and prescribing will be managed by a software application securely accessed by authorised study personnel [57]. Study medication will not be stored at participating NHS sites prior to dispensing. Participants will return the packaging of used study medication and any unused pens to the local hospital site for accountability and destruction by the research team. Used study medication pens will be disposed of in sharps bins by the participants at home. Sciensus are responsible for providing and disposing of sharps bins.

Participant training & delivery
Sciensus will use their network of trained nurses to deliver training to participants in their homes about self-administration of the study medication at the start of the TRI period. In exceptional circumstances participants can receive training from appropriately qualified staff at their referring hospital. This must be discussed with the trial team prior to participant enrolment and have approval from both the PI and the CI.

Sciensus will use their fully traceable, cold-chain supply network to deliver study medication to participants' homes for the duration of the trial. Sciensus deliver commercial Imraldi™ and train patients who receive the drug as part of standard NHS care and as such are experienced in providing these services and have processes in place for reporting AEs or issues with specific pre-filled pens to patients' care teams (i.e. hospital sites).

Both the IMP and placebo will be labelled in accordance with Annex 13. The contents of the label will be submitted to the MHRA for approval. Storage of the IMP, both drug and placebo, will be in accordance with the SmPC/dossier (Appendix 1). The IMP will be accounted for according to NHS pharmacy standards.

Reference safety information
The SmPC Imraldi 40mg solution for injection in a pre-filled pen, Section 4.8 dated 16 Aug 2021, will be used as the reference Safety Information (RSI).

In accordance with the SmPC, participants cannot be administered live vaccines for the duration of their time on the trial intervention.

5.8 Primary and secondary outcomes

5.8.1 Primary outcome
The primary outcome is time to the first treatment failure (TF), for a participant, i.e. TF may occur in either eye and may be triggered by incident ANIU in an eye that did not previously have ANIU. TF is defined as a composite of standard criteria reflecting clinical decision-making, including visual acuity and clinical signs of active inflammation which have been used successfully in other ANIU trials [1, 4, 58].

Our definition of TF will modify the composite endpoint used in the VISUAL trials [1,2] by: (i) excluding isolated anterior uveitis as a TF event (it can be treated effectively and safely with low dose, low frequency topical CS); and (ii) including a clinically important deterioration in CMO as a TF event [11], because it is an ANIU complication causing visual loss in a third of patients [59] which has often studied as a TF event in other trials of ANIU.

Participants will be assessed for TF at each visit after randomisation.

Any of the following criteria in one or both eyes, where applicable, will constitute TF:

i. >=15 letter decrease in best corrected visual acuity (BCVA), compared to BCVA measured by an optometrist masked to treatment allocation at the 16 week TRI visit;
ii. new active inflammatory chorioretinal lesions (masked review of fundus colour and autofluorescence images by clinician during the RCT; unmasked during the OLE);
iii. >20% increase in central macular thickness (CMT), compared to CMT at the 16 week TRI timepoint (CMT is a parameter that is measured by an automated algorithm when doing optical coherence tomography (OCT));
iv. onset or worsening of retinal vasculitis (masked clinician assessment of fundus fluoroscein angiogram during the RCT; unmasked during the OLE);
v. 2 step worsening of vitreous haze compared to score at the 16-week TRI visit (masked clinician assessment during the RCT; unmasked during the OLE);
vi. prescription by a masked clinician of >5mg/day oral prednisolone to maintain uveitis remission (i.e. to avert relapse before any of the above criteria for manifest active disease (i-v) during the RCT; unmasked during the OLE).

Prescription of >5mg/day oral prednisolone for a co-morbidity (e.g. rheumatic disease) but with none of i-v being present does not constitute TF.
If the participant experiences any of criteria i-vi within 14 days of having intraocular surgery, it does not constitute TF. If the criteria persists beyond 14 days (except >5mg/day oral prednisolone) then it does constitute TF.

The arrangements for managing a participant's continued follow-up in the trial following TF have been described above, including switching treatment allocation without unmasking allocation (see 5.1 and 5.7).

5.8.2 Secondary outcomes

Secondary outcomes will include:

a) Individual TF components, assessed at each trial visit;
b) Retinal morphology (OCT; macular and retinal nerve fibre layer), assessed at each trial visit;
c) AEs, assessed at each trial visit;
d) HRQoL measured using the EQ-5D-5L questionnaire [3][7] at the start of TRI, at 16 weeks immediately before randomisation, then 12-weekly after randomization up to week 48 and 16-weekly thereafter;
e) Patient-reported symptoms of side-effects at the start of TRI, at 16 weeks (immediately before randomisation), then at each trial visit after randomisation and at any interim attendance prompted by an AE;
f) Patient-reported visual function [2], at the start of TRI, at 16 weeks immediately before randomisation, 12-weekly up to week 48 and 16 weekly thereafter;
g) Best corrected visual acuity (BCVA) assessed at each trial visit;
h) Employment status at the start of TRI, at 16 weeks immediately before randomisation, 12-weekly up to week 48 and 16 weekly thereafter;
i) Resource use at the start of TRI, at 16 weeks immediately before randomisation, and every 12 weeks (up to week 48) or 16 weeks (after week 48) during follow-up after randomisation.

5.9 Sample size justification
We hypothesise that participants randomised to receive adalimumab will have a lower risk of TF than those allocated to receive placebo. The sample size has been chosen to test this hypothesis. In estimating the sample size, we have considered the results observed in previous studies; hazard ratios (HRs) for time to first TF in two previous trials of adalimumab (in patients with active and inactive ANIU, respectively) were 0.50 and 0.57 [1, 4]. As our study will only randomise participants who are found to respond to treatment in the TRI phase, we anticipate that we will observe a greater benefit than in these trials, which included both responders and non-responders. Therefore, we have set the target HR at 0.5. Assuming that 27% in the placebo group survive free from TF 12 months after randomisation (estimated from placebo groups of the two trials with 40% active and 60% inactive disease), we have set a target sample size of 174 randomised participants. A study of this size will have 90% power to detect a HR 0.5 at a 5% two-sided significance level, allowing for 10% loss-to-follow-up in the first 12 months.

6. Trial methods

6.1 Description of randomisation
Eligibility will be checked, and consent obtained to the whole study at the beginning of the TRI, with both being confirmed at the 16-week visit when responder status has been ascertained and eligibility for the trial has been confirmed. Randomisation will be carried out at this visit so that the appropriate IMP can be prescribed at the same time. Randomisation will be performed by a member of the local research team on the delegation log who is authorised to do so using a secure internet-based randomisation system ensuring allocation concealment. Participants will be allocated in a 1:1 ratio to either adalimumab or placebo.

The random allocations will be computer-generated by a statistician in the trials unit in blocks of varying size (unknown to trial personnel) and stratified by centre, before the trial starts to recruit. The allocations will be embedded in the trial database and concealed from all clinical and research personnel until a participant has been recruited. Key data to characterise a participant’s current clinical status at will be collected at the time of randomisation.

6.2 Masking and unmasking
During the RCT, participants, their clinical care team (i.e. their ophthalmologist and other members of eye care team) and the research nurse(s) responsible for participant follow-up, will all be masked to participants’ allocations. Except for the study statistician, clinical trials staff managing the trial will also be masked to allocation. The randomisation system will provide a unique code which the study pharmacy (Sciensus, formerly called Healthcare at Home, Ltd) will use to identify the pens to be dispensed. It will not be possible to distinguish between syringes containing drug and placebo dispensed during the RCT on the basis of their appearance or any other characteristics. No one will be masked during the OLE.

The IMP will be injected subcutaneously by participants at home. The prefilled syringes will look identical and we are not aware of any sensation from self-injection of adalimumab that will differ from injection of placebo. Adalimumab may induce side-effects in some patients that will inadvertently unmask participants. We acknowledge this may be a limitation of the study. We will collect information to document the extent to which this happens.

Participants will be made aware before entering the study that they will not be told which treatment they will receive during the RCT. Doctors will prescribe the ‘study medication’ with a unique code; appropriate pens, corresponding to the code, will be dispensed to participants. Bristol Trials Centre (BTC) and other designated personnel will be able to break the code in the

event of an emergency but will not know the allocation in the usual course of dispensing a prescription for trial IMP.

If clinically indicated (i.e. in the event of a serious adverse event (SAE) requiring knowledge of the allocation for treatment) the treatment allocation will be unmasked by the BTC during office hours or by the on-call pharmacist at UHBW. Unmasking will be done using the secure web-based IMP-Track IMP management and accountability system [57]. Only authorised personnel will have access to the IMP-Track system. Any request for unmasking will be fully documented, recording the identity of the person asking for the allocation to be unmasked and the reason for unmasking. Instances of unmasking will be monitored throughout the trial.

6.3 Research procedures

Dedicated uveitis clinics are run in each centre, which will consider all new referrals. Medical and uveitis history will be recorded, screening investigations will be ordered and eligibility will be assessed.

Potentially eligible patients will be consented (to screening and the full study on one consent form) prior to full screening assessments (as per Table 1. Schedule of events during the trial) being done. Patients who are eligible to take part after all required screening assessments are done will be enrolled in the TRI after confirming they are still willing to take part.

Data will be collected on the numbers screened, eligible pre- and post-consent and starting the TRI, including reasons for declining participation. Data will be captured in a purpose-designed secure database, with 'real time' validation, which will be developed by the BTC to support the trial. Resource use data will be collected using bespoke questionnaires and trial case report forms (CRFs).

Participants starting the TRI will be seen at 4 weeks (at hospital), 8 weeks (telephone call review) and 16 weeks (at hospital). This visit frequency approximates usual care, when a patient starts treatment with adalimumab. Study assessments will be completed as per the Schedule of events during the trial (table 1).

At the 16 week visit, if a participant is classified as a responder (see 6.3.1) and agrees to continue in the randomised trial, he/she will be randomised (see 5.7, 5.8.1 and 6.1).

Participants who meet the responder criteria will be randomised into the RCT and will have follow-up visits will be scheduled at 12-weekly intervals up to 48 weeks after randomisation and 16-weekly intervals thereafter, consistent with usual care for patients with ANIU.

If treatment failure is suspected in between 12-weekly follow up visits, an unscheduled treatment failure assessment visit should be conducted to include all follow-up assessments as per the schedule of events. If at an unscheduled visit, TF is NOT confirmed to have occurred the participants will still need to attend the next scheduled visit and all assessments conducted as per protocol.

Participants who consent to donate optional blood serum samples will have these taken at baseline, TRI 16 week visit, (both responders and non-responders) and, if randomised, 48 week visit and 96 week visits in the RCT, and at any post randomisation visit where a participant experiences TF (whether this is observed in the RCT or OLE).

Participant who consent to optional whole blood DNA samples will have these taken at baseline.

Optional serum and DNA will be prepared from the blood samples and stored. These samples, and retinal images taken as part of the study, will be collected and stored as a resource for future studies of markers for (a) responsiveness to adalimumab and (b) TF.

At their last visit before expiry of the placebo, participants who give consent will transition to the OLE (see 5.1 and 5.3). Participants in the OLE will continue with the same visit and treatment schedule as they had in the RCT until they either experience TF (when they will exit the trial) or they reach the end of the OLE period (when they will exit the trial at the last visit before 31st March 2026).

6.3.1 Responder criteria

A responder will be defined as meeting ALL of the following criteria at the 16 week TRI visit:

i. No activity on colour fundus or autofluorescence imaging indicative of inflammatory chorioretinal lesions;
ii. CMT ≤ 320µm on any OCT machine and no evidence from OCT of cystoid macular oedema (CMO)* [11];
iii. No evidence of active retinal vasculitis [11];
iv. ≤ 0.5 vitreous haze Binocular Indirect Ophthalmoscopy (BIO) score;
v. Prescription of ≤ 5.0mg/day oral prednisolone to achieve/maintain uveitis remission [11] (participant is a responder if prescribed >5.0mg/day oral prednisolone for a co-morbidity (e.g. rheumatic disease) where ALL of i-iv are met).

* An eye can be classified as a responder if CMT > 320µm providing that there is full clinical resolution, i.e. complete resolution of macular thickening, cysts and intra/subretinal fluid.

6.3.2 Investigations during follow-up

Investigations relating to eligibility, data about safety of medications, refraction and imaging will be carried out during the TRI. The following investigations will be carried out at all visits after randomisation (see Table 1):

a) Ophthalmic examination, including slit lamp examination with dilated fundoscopy and tonometry;
b) Vital signs / weight (every other follow-up visit);
c) Changes in medications, AEs, adherence to medications
d) BCVA (ETDRS letter chart) determined by an optometrist masked to treatment allocation;
e) Retinal imaging, comprising OCT, macular and retinal nerve fibre layer;
f) Colour fundus imaging in all participants, and autofluorescence and fundus fluorescein angiogram in those where it was required to determine if disease was active or not at baseline.*
g) In addition, the following patient-reported outcome measures will be collected: EQ-5D 5L; VCM1; Work Productivity and Activity Impairment Questionnaire: Specific Health Problem Questionnaire (WPAI-SHP); symptoms of side effects.

*If AF/FFA are required to determine that disease is active or not at baseline, these tests will need to be repeated when establishing responder status or TF.

6.4 Duration of treatment period

As described in section 5.1, we have chosen to try to keep participants in the trial until the trial will be close to reporting [53]. Treating with adalimumab for longer than 48 weeks is consistent with how adalimumab is used in practice in patients who are maintained on it successfully, so will contribute valuable longer term evidence about the intervention. This approach is likely to foster better commitment among participants and is supported by the PPI panel.

6.5 Definition of end of trial

The RCT will end for a participant after they have completed follow up (including repeat TRIs) for a minimum of 12 weeks up to a maximum of 170 weeks (approx. between 3 and 40 months) post-randomisation, or earlier if they withdraw from the study completely. The OLE will extend

follow-up for another 76 weeks (approx. 18 months). The maximum period any participant could be in the trial is 246 weeks (approx. 58 months).

The end of the RCT will be after all trial participants have completed placebo-controlled follow up in the RCT, all data queries have been resolved and the RCT part of the database has been locked. The end of the OLE will be after all trial participants have completed the last follow up before ending the study, all data queries have been resolved and the OLE part of the database has been locked. Data from the OLE will be used to update estimates of treatment effectiveness.

Data collection

Data collection will include the elements described in Table 1.

Table 1 Schedule of events during the RCT and OLE

			**Treatment run-in**				**RCT / OLE**					
**No.**	**Data item to be collected**	**Screen/ baseline**	**1st injection (wk 0)**	**4 w**	**8 w** (phone call)	**16 w / Rx**	**12 w**	**24 w**	**36 w**	**48 w**	**Then:**	**Unsch.** (*6)
1.	Confirm eligibility	✓				✓						
2.	Consent confirmed	✓				✓*3						
3.	Medical history	✓										
4.	Baseline characteristics	✓										
5.	Vital signs	✓				✓	✓	✓	✓	✓	16 w+	✓
6.	Weight	✓				✓		✓		✓	16 w+	✓
7.	History directed medical exam	✓				✓	✓	✓	✓	✓	16 w+	✓
8.	Full blood count (FBC)	✓		✓		✓	✓	✓	✓	✓	16 w+	✓
9.	Liver function tests	✓		✓		✓	✓	✓	✓	✓	16 w+	✓
10.	Electrolytes profile	✓		✓		✓	✓	✓	✓	✓	16 w+	✓
11.	Glucose test	✓		✓								
12.	TB IGRA test	✓										
13.	Chest X-ray	✓										
14.	12-lead ECG*1	✓										
15.	MRI*1	✓										
16.	Syphilis test	✓										
17.	HIV test	✓										
18.	Hep B / Hep C test	✓										
19.	Lyme IgG/IgM Antibody Serology*1	✓										
20.	Varicella history	✓										
21.	Pregnancy test (women only)	✓										
22.	BCVA	✓		✓		✓	✓	✓	✓	✓	16 w+	✓
23.	OCT	✓				✓	✓	✓	✓	✓	16 w+	✓

			Treatment run-in				RCT / OLE					
**No.**	**Data item to be collected**	**Screen/ baseline**	**1st injection (wk 0)**	**4 w**	**8 w** (phone call)	**16 w / Rx**	**12 w**	**24 w**	**36 w**	**48 w**	**Then:**	**Unsch.** (*6)
24.	Fundus colour imaging	✓				✓	✓	✓	✓	✓	16 w+	✓
25.	Autofluorescence (AF) imaging	✓				✓*2	✓*2	✓*2	✓*2	✓*2	16 w+*2	✓*2
26.	Fundus fluorescein angiogram (FFA)	✓				✓*2	✓*2	✓*2	✓*2	✓*2	16 w+*2	✓*2
27.	Clinical exam (including slit-lamp examination and indirect ophthalmoscopy)	✓		✓								
28.	Clinical exam for treatment failure (including slit-lamp examination and indirect ophthalmoscopy)					✓	✓	✓	✓	✓	16 w+	✓
29.	Telephone wellbeing review				✓							
30.	Adverse events			✓	✓	✓	✓	✓	✓	✓	✓	✓
31.	EQ-5D-5L	✓				✓	✓	✓	✓	✓	16 w+	✓
32.	VCM1	✓				✓	✓	✓	✓	✓	16 w+	✓
33.	Symptoms of side-effects	✓				✓	✓	✓	✓	✓	16 w+	✓
34.	WPAI-SHP questionnaire	✓				✓	✓	✓	✓	✓	16 w+	✓
35.	Blood sample (for serum)*4	✓				✓	✓*4	✓*4	✓*4	✓	48 w+*4	✓*4
36.	Blood sample (for DNA)	✓										
37.	Resource use					✓	✓	✓	✓	✓	16 w+	✓

- Blood tests done as standard care can be accepted for screening up to a maximum of 3 months prior to the date of the screening visit. This can be extended in exceptional circumstances with approval of both the PI and CI.
- Chest x-ray, MRI and ECG done as standard care can be accepted for screening up to a maximum of 6 months prior to the date of the screening visit. This can be extended in exceptional circumstances with approval of both the PI and CI.
- Historic fundus colour, AF and FFA images can be accepted at baseline up to a maximum of 6 weeks prior to the date of the screening visit. This can be extended in exceptional circumstances with approval of both the PI and CI. OCT must be carried out at screening/baseline visit

- AF and FFA may not be necessary at baseline for those patients who do not need those assessments in order to support the clinical decision about TF. This is at the discretion of PI when determining activity or endpoint constitution of TF for individual patients.
- If AF/FFA are required to determine that disease is active or not at baseline, these tests will need to be repeated when establishing responder status or TF.
- Enrolment in the TRI (i.e. writing the TRI prescription) must be done up to 4 weeks after the date of the screening visit, but can be extended to 6 weeks in exceptional circumstances. If a patient is not enrolled within 6 weeks, the baseline ocular exams and imaging will need to be repeated to determine eligibility.
- TRI follow-ups start from the date of 1st injection (time 0); RCT follow-up starts from the date of randomisation. Repeat TRI follow ups start from date of treatment response assessment visit; repeat RCT follow up starts from date of treatment response assessment visit; OLE follow up starts from date of final RCT assessment visit.
- Visit windows: TRI +7 days; RCT -14 days and +7 days; OLE -14 days and +7 days.

*[1] Only required if clinically indicated.
*[2] Only required if undertaken at baseline to support the clinical decision about TF.
*[3] Written informed consent will be obtained at the start of the TRI; this consent will be confirmed among responders at the start of the RCT.
*[4] Only taken at these time points in the event of treatment failure being detected.
*[5] If patient has no history of varicella, advise to avoid people with chickenpox or shingles for duration of time on study medication.
*[6] If TF is suspected in between RCT follow up visits the participant should attend an unscheduled visit where the research team must complete all assessments as per scheduled RCT follow-up visits. IF TF is confirmed not to have occurred participants must still attend the next scheduled visit and all assessments conducted as per protocol.

BCVA – best corrected visual acuity; **TF** – treatment failure; **OCT** – optical coherence tomogram; **Rx** – Randomisation; **VCM1** – Visual Function Questionnaire; **WPAI-SHP** – Work Productivity and Activity Impairment Questionnaire: Specific Health Problem Questionnaire; **w** - weekly; **w+** - weekly thereafter

6.6 Source data

Source data will differ for different items of data.

- Items 1-22, 27-30 and 37: paper CRFs designed specifically to capture the required data for the study (item 37 prescribed medication source data is the eCRF);
- Items 23-26: OCT, fundus, autofluorescence and fundus fluorescein angiography images submitted for independent grading;
- Items 31-34: Completed questionnaires returned by participants;
- Items 35-36: not applicable; samples for storage only (for future research).
- IMP accountability: IMP-Track Investigational Medicinal Product Management System

6.7 Planned recruitment rate

Projecting recruitment is complicated because: (a) estimates of several parameters are uncertain, (b) participants will be recruited with both incident (active) and prevalent (inactive) ANIU and (c) the TRI design causes a lag. Table 2 shows our projection with the assumptions described in a footnote.

6.8 Participant recruitment

Patients with ANIU present to, and are managed by, specialist ophthalmologists staffing clinics in tertiary hospitals which treat autoimmune ophthalmic diseases. Clinics will include new patients with ANIU and existing patients who are having ongoing treatment monitored. The eligibility criteria allow recruitment of either kind of patient. New patients started on treatment with oral prednisolone will be eligible, with an attempt being made to reduce oral prednisolone to ≤5.0 mg over 16 weeks alongside treatment with adalimumab. Existing patients who are already being treated but who require >5mg/day oral prednisolone will also be eligible, with an attempt being made to reduce oral prednisolone to ≤5.0 mg over 16 weeks alongside treatment with adalimumab. Both new patients and existing patients may be prescribed other non-biologic IMT in addition to oral prednisolone at the discretion of the clinician.

Potential participants will initially be screened from their medical records by the direct care team according to local hospital processes. Patients who are initially eligible will be given or sent an invitation letter and a patient information leaflet (PIL) describing the study. Potential participants will be given time to read the PIL and to ask questions that they may have; the person taking consent will discuss the trial with the potential participant prior to consent. Consent will be taken in clinic. The PIL will describe both the TRI period and the RCT, and the informed consent form (ICF) will include consent to both phases of the trial. Consented participants will undergo further screening assessment to determine their full eligibility to participate. Consented participants who meet all eligibility criteria will be enrolled in the TRI. Eligibility will be confirmed by a clinician on the delegation log. Participants who are classed as responders at the end of the TRI will be asked if they are willing to continue to the randomised phase of the study, before carrying out randomisation; their willingness to continue will be recorded at the time.

At their last visit before expiry of the placebo, participants who give consent will transition to the OLE.

Table 2 projected recruitment of participants with active and inactive ANIU

Trial month	Trial phase	Main trial activity	Number of centres	ANIU eligible for TRI		Number randomised	
				Inactive	Active	per month	cumulative
1 to 7	Pilot	Setup	0	0	0	0	0
8	Pilot	Recruit; TRI only	3	4	1	0	0
9	Pilot	Recruit; TRI only	5	6	2	0	0
10	Pilot	Recruit; TRI only	7	9	3	0	0
11	Pilot	Recruit; TRI only	9	11	4	0	0
12	Pilot	Recruit & rand'x	11	14	5	2	2
13	Pilot	Recruit & rand'x	13	16	6	4	6
14	Pilot	Recruit & rand'x	15	19	7	5	11
15	Pilot	Recruit & rand'x	17	21	8	7	18
16	Pilot	Recruit & rand'x	19	24	9	8	26
17	Pilot	Recruit & rand'x	19	24	9	10	36
18	Pilot	Recruit & rand'x	19	24	9	11	47
19	Pilot	Recruit & rand'x	19	24	9	13	60
20	Main RCT	Recruit & rand'x	19	20	9	14	74
21	Main RCT	Recruit & rand'x	19	18	9	14	88
22	Main RCT	Recruit & rand'x	19	15	9	14	102
23	Main RCT	Recruit & rand'x	19	13	9	14	116
24	Main RCT	Recruit & rand'x	19	10	9	13	129
25	Main RCT	Recruit & rand'x	19	8	9	12	141
26	Main RCT	Rand'x only	19	0	0	10	151
27	Main RCT	Rand'x only	19	0	0	9	160
28	Main RCT	Rand'x only	19	0	0	7	168
29	Main RCT	Rand'x only	19	0	0	7	174
30 to 41	Main RCT	Follow-up only	19	0	0	0	174
42	Main RCT	follow-up	19	0	0	0	174
43	Main RCT	d-b lock	19	0	0	0	174
44 to 46	Main RCT	analysis	19	0	0	0	0
47 to 48	Main RCT	write-up	19	0	0	0	0
**Total**				**285**	**129**	**174**	

Footnote: rand'x – randomise; annual incidence, 0.0003 [30]; prevalence, 0.00083 [30]; ANIU proportion >20yrs, 0.763; total incident cases,12541; total prevalent cases, 34696; centres treating ANIU, 30; incident ANIU/centre/year, 418; prevalent ANIU/centre/year, 1157; p("eligible"), 0.06; p(approached), 1.00, p(ineligible after approach), 0.20; p(consenting for TRI), 0.40; p(responding), 0.50; p(available to randomise at end of TRI), 0.15; number of centres, 19.

6.9 Discontinuation/withdrawal of participants

As described in section 5.1, we will try to keep all participants in the RCT until the last participant has completed the 12-week visit in the RCT. Participants who consent to join the OLE may be followed for up to a further 76 weeks (approx. 18months). Each participant has the right to withdraw at any time. In addition, the investigator may withdraw the participant from treatment with adalimumab/placebo if continuation is considered not to be in the best interests of the participant, e.g. following a SAE or pregnancy. Participants must also stop treatment with

adalimumab/placebo after a maximum of two treatment switches (see section 5.1). In all instances of stopping treatment with adalimumab/placebo we will request that participants continue to attend the scheduled research visits so that the relevant data can be collected. All data and samples collected will be analysed and stored unless a participant expressly requests that his/her data or samples be destroyed.

6.10 Frequency and duration of follow up

The follow-up schedule is described in Table 1, Section 0.

6.11 Likely rate of loss to follow-up

Assuming that 27% in the placebo group are free from TF 12 months after randomisation (estimated from placebo groups of the two trials with 40% active and 60% inactive disease [1, 4]), we have set a target sample size of 174 randomised participants. A study of this size will have 90% to detect a HR 0.5 at the 5% two-sided significance level, allowing for 10% loss-to-follow-up in the first 12 months.

There are no special features to minimise bias due to missing outcome data. Established methods will be used to maximise the proportion of participants for whom all outcome data are available and the proportion of participants who receive the intervention to which they were allocated. However, ANIU is a debilitating condition for patients who are affected and, subject to other comorbidities they experience, we expect participants to attend their follow-up visits as scheduled; these are set at the same frequency as required for usual care so extra research-only visits should be unnecessary.

6.12 Expenses

The schedule of follow-up visits has been designed to be the same as for usual care for a patient with ANIU. Therefore, it is not expected that participants will be reimbursed for travel expenses. Participants may be reimbursed for the cost of parking if a visit takes longer than a usual care visit as a direct result of research-specific procedures (i.e. assessments they would not have had if they were not taking part in the trial).

7. Statistical analyses

7.1 Plan of analysis

The primary analysis of effectiveness will be restricted to data collected in the RCT. The data will be analysed according to intention to treat (ITT) and follow CONSORT reporting guidelines. Analyses will be adjusted for centre. The primary outcome, time from randomisation to first TF in the RCT, will be compared using survival methods, allowing for censoring of any participant who is lost to follow-up. Secondary outcomes up to the time of TF will be compared using a mixed linear or logistic regression model as appropriate, adjusted for baseline measures when available. These outcomes will be modelled jointly with the time to first TF. Changes in treatment effect with time will be assessed by adding a treatment x time interaction to the model and comparing models using a likelihood ratio test. Model fit will be assessed and alternative models and/or transformations (e.g. to induce normality) will be explored where appropriate. Frequencies of AEs will be described. Treatment differences will be reported with 95% CIs.

A secondary analysis will be carried out at the end of the OLE, including data from both the RCT and OLE, updating the primary analysis estimates. If additional first TFs are observed in the OLE, these will increase the precision of updated estimates of treatment effectiveness.

A further secondary analysis will include follow-up time after the first TF. A participant who experiences a first TF will restart adalimumab (repeating the TRI) for 16 weeks. If after 16 weeks the participant satisfies the criteria for being a responder, he/she will re-enter the trial treated with the opposite treatment to the one originally allocated (placebo if originally on intervention, or vice versa). Restarting treatment will become the time origin for the second period of follow-up. (This process can be repeated another time, in the event of a second TF, generating up to three discrete "disconnected" periods of follow-up. Follow-up will cease after a third TF.

The allocation group (and other important covariates) will be updated when "study" treatment (intervention or placebo) is restarted. We will analyse multiple periods of treatment exposure within participants with survival models. The primary analysis, i.e. time to first TF will be unbiased. Analyses of subsequent follow-up periods must be considered to be non-randomised, and potentially biased, since participants' characteristics are likely to predict TF. Therefore, if adalimumab is effective, second and third follow-up periods will not be distributed in a balanced way across the original randomly assigned groups and it will be necessary to consider important covariates. Nevertheless, switches will provide a within-subject comparison of survival free from TF between intervention and placebo.

A detailed analysis plan will be prepared.

7.2 Subgroup analyses

No subgroup analyses are planned.

7.3 Frequency of analyses

The primary analysis will take place when follow-up is complete for all recruited participants. No formal interim analysis is planned. Safety data will be reported to the Data Monitoring and Safety Committee (DMSC) at least annually, together with any additional analyses the committee request. In these reports the data will be presented by group, but the allocation will remain masked.

Interim analyses will be decided in advance in discussion with the Data Monitoring and Safety Committee (DMSC). There is no intention to compare any outcomes between groups at the end of the internal pilot phase; the only analyses will be descriptive statistics to summarise eligibility and recruitment to decide whether the trial satisfies the progression criteria.

7.4 Criteria for the termination of the trial

The trial may be terminated early by the Trial Steering Committee. A decision to terminate may arise from a recommendation by the DMSC to stop the trial, for example based on *an interim analysis of the data from the trial* or if the results of another study make the completion of the trial unnecessary.

7.5 Economic issues

The within-trial economic evaluation will aim to determine the relative cost-effectiveness of adalimumab compared to usual care. We will carry out the cost-effectiveness analysis largely from the perspective of the UK NHS but will also aim to explore costs to society in the form of productivity cost changes.

Established guidelines will be used for the conduct of the economic evaluation [60]. The main outcome measure for the economic evaluation will be quality adjusted life years (QALYs), estimated using the EuroQol EQ-5D 5L, administered at baseline (start of the TRI; n≈400), at 16

weeks (time of exiting the study because of non-response or randomisation; n≈400), then at each subsequent visit (n<=174), i.e. 3-monthly up to 12 months and 4 monthly thereafter). Respondents will be assigned valuations derived from published UK population tariffs [61] and the mean number of QALYs per trial arm and incremental QALYs will be calculated.

For the costing component, resource use data will be collected by adding questions to the trial CRFs. Resource use information will be collected on quantities of drugs of financial significance administered in the two alternative trial arms and resources associated with treating any of these drug side-effects, including hospital admissions and additional outpatient visits and GP visits. We will also collect information on patient employment status during the trial in order to estimate productivity cost changes. Unit costs to be added to the resource use information will be taken from nationally published sources such as the BNF and Emit for drug costs and the NHS Reference Costs for any hospital admissions.

The analysis will calculate the average cost and outcome on a per patient basis and use this information to estimate the incremental cost-effectiveness ratios for the different trial arms will be derived, producing an incremental cost per QALY. Probabilistic sensitivity analysis will be used to demonstrate the impact of the variation around the key parameters in the analysis on the baseline cost-effectiveness results. A key sensitivity analysis will be the cost of adalimumab, where we will explore the use of potentially cheaper biosimilars. Results will be expressed in terms of a cost-effectiveness acceptability curve.

8. Trial management

University Hospitals Bristol NHS Foundation Trust (UHBW) will act as Sponsor. Responsibility for running the RCT will be delegated via an agreement with the University of Bristol. Agreements between the Sponsor and participating centres will be required, as well as standard site-initiation documents, before recruitment commences. The study will be conducted in accordance with Good Clinical Practice (GCP) guidelines, the European Union Directive 2001/20/EC on clinical trials, the Data Protection Act and the UK Policy Framework for Health and Social Care Research. The trial will be registered on an open access clinical trial database (ISRCTN). Clinical trial documents will be archived and held by the Sponsor for 15 years after study closure in accordance with the standard operating procedures of the Sponsor and in compliance with the principles of GCP.

The study will be managed by the CI, clinical and BTC co-applicants, PPI representative and the trial manager, and fully supported by the wider BTC (a UK Clinical Research Network registered clinical trial unit, Reg. No 11 (registered as the Clinical Trials and Evaluation Unit (CTEU). The CTEU has been assimilated into the BTC). The BTC has an established track record of designing, conducting, managing and reporting multi-centre clinical trials in ophthalmology, including trials of investigational medicinal products (e.g. the IVAN and VICI trials, NIHR refs 07/36/01 and 13/94/15). The BTC has experience in building study database systems and providing randomisation services.

The CI and BTC team will work with the co-applicants to prepare the final protocol, submitting the Ethics/HRA, MHRA and local application packs for each site, preparing trial manuals, providing the randomisation service and designing and implementing the data management system. The CI, BTC team and project Sponsor will endeavour to ensure that the trial runs according to the pre-agreed timetable, recruitment targets are met, the CRFs are completed accurately, complies with relevant ethical and other regulatory standards, and that all aspects of the study are performed to the highest quality. Clinical co-applicants and BTC team members

will also train investigators at participating centres, check that centres are ready to start ("green light") and monitor their progress during the study. The trial manager will be the contact point to provide support and guidance to the participating centres/specialties throughout the study.

8.1 Day-to-day management

The trial will be managed by a Trial Management Group (TMG), which will meet face-to-face or by teleconference approximately monthly. The TMG will be chaired by a Chief Investigator and will include all members of the named research team (see Chief Investigator & Research Team Contact Details).

An appropriately qualified person by training will be responsible at each site for identifying potential trial participants, seeking informed participant consent, randomising participants, liaising with pharmacy, collecting trial data and ensuring the trial protocol is adhered to.

8.2 Monitoring of sites

8.2.1 Initiation visit

Before the study commences, training session(s) will be organised by BTC. These sessions will ensure that personnel at each site involved fully understand the protocol, CRFs and the operational requirements of the study.

8.2.2 Site monitoring

The trial coordinating centre will carry out regular monitoring and audit of compliance of centres with GCP and data collection procedures described in section 0. Monitoring of data collection will be via the study database (checks for data completeness and routine data query review), which will be carried out on a regular basis. The ISF and CRFs will be monitored by site self-completed checklists at least once in the lifecycle of the trial. The TMG will review accumulating data on, including but not limited to, screening, eligibility, recruitment, data completeness, adherence to follow-up, adverse events and protocol deviations in the form of central monitoring reports generated approximately monthly.

8.3 Trial Steering Committee and Data Monitoring and Safety Committee

An independent Trial Steering Committee (TSC) will be established to oversee the conduct of the study. It is anticipated that the TSC will comprise the CI and co-lead investigator, an independent chair and at least two additional independent members, at least one of whom will be a patient/public representative. The TSC will develop terms of reference outlining their responsibilities and operational details. The TSC will meet before recruitment begins and regularly (at intervals to be agreed by the TSC but a minimum of once per year) during the course of the study.

An independent DMSC will be established to review safety data during the course of the study and will advise on interim analyses. The DMSC will develop a charter outlining their responsibilities and operational details. The DMSC will meet (before or jointly with the TSC) before the trial begins and they will meet regularly thereafter (at intervals to be agreed by the DMSC but a minimum of once per year). Stopping rules for the trial will be discussed at the first DMSC meeting, and decisions documented in the DMSC Charter.

9. Safety reporting

SAEs and AEs will be recorded and reported in accordance with GCP guidelines and University Hospitals Bristol and Weston NHS Foundation Trust (UHBW) Research Safety Reporting Standard Operating Procedure (see Figure 3).

Expected events are those associated with adalimumab and are listed in the SmPC (see section 9.1). Events that are fatal or life threatening will follow the same reporting procedure as for unexpected events.

All AEs will be recorded on CRFs. The investigator will notify all SAEs to the Bristol Trials Centre within 24 hours of knowledge of the event. The Bristol Trials Centre will report all SAEs to the Sponsor within 24 hours of being notified of the event by the investigator. If the event is expected or is unexpected but not causally related to the intervention, the Bristol Trials Centre will report to the REC, MHRA and DMSC at least annually. If the event is unexpected and causally related to the intervention (suspected unexpected serious adverse reaction (SUSAR)) reporting will be expedited according to the schedule in Figure 3; the Sponsor will report to the MHRA and the Bristol Trials Centre will report to the REC and the DMSC.

In the event that personnel from Sciensus, formerly called Healthcare at Home Ltd are made aware of an adverse event they will report the event to the prescribing investigator according to their standard operating procedure. The investigator will then follow the safety reporting schedule in Figure 3.

The Sponsor delegates assessment of expectedness and relatedness to the investigator but will also review each reported SAE and confirm this assessment.

For all SAEs that are ongoing at the time of the initial report, the participant will be actively followed up, and the investigator (or delegated person) will provide a follow-up report within five working days after the initial report and further follow-up reports as new information becomes available until the SAE has resolved.

Elective surgery or intervention(s) during the trial that were planned prior to recruitment to the trial will not be reported as an SAE.

9.1 Expected adverse events associated with the study medication

The SmPC for Imraldi™ 40mg solution for injection in using pre-filled pen (Biogen GmbH) Section 4.8 dated 16-August-2021 forms the reference safety information and is approved by the MHRA for this trial.

Any updates made to section 4.8 of the SmPC will be reviewed by the CI and, in consultation with the Sponsor, a decision made whether the updated document will be submitted to the MHRA for use as the RSI for the trial.

9.2 Period for recording serious adverse events

Data on AEs will be collected from the start of the TRI (first IMP administration) until 1) the end of the TRI for participants who do not respond and leave the trial at this stage or, 2) the end of the follow-up period in the RCT/OLE for all participants who respond and progress into the RCT/OLE.

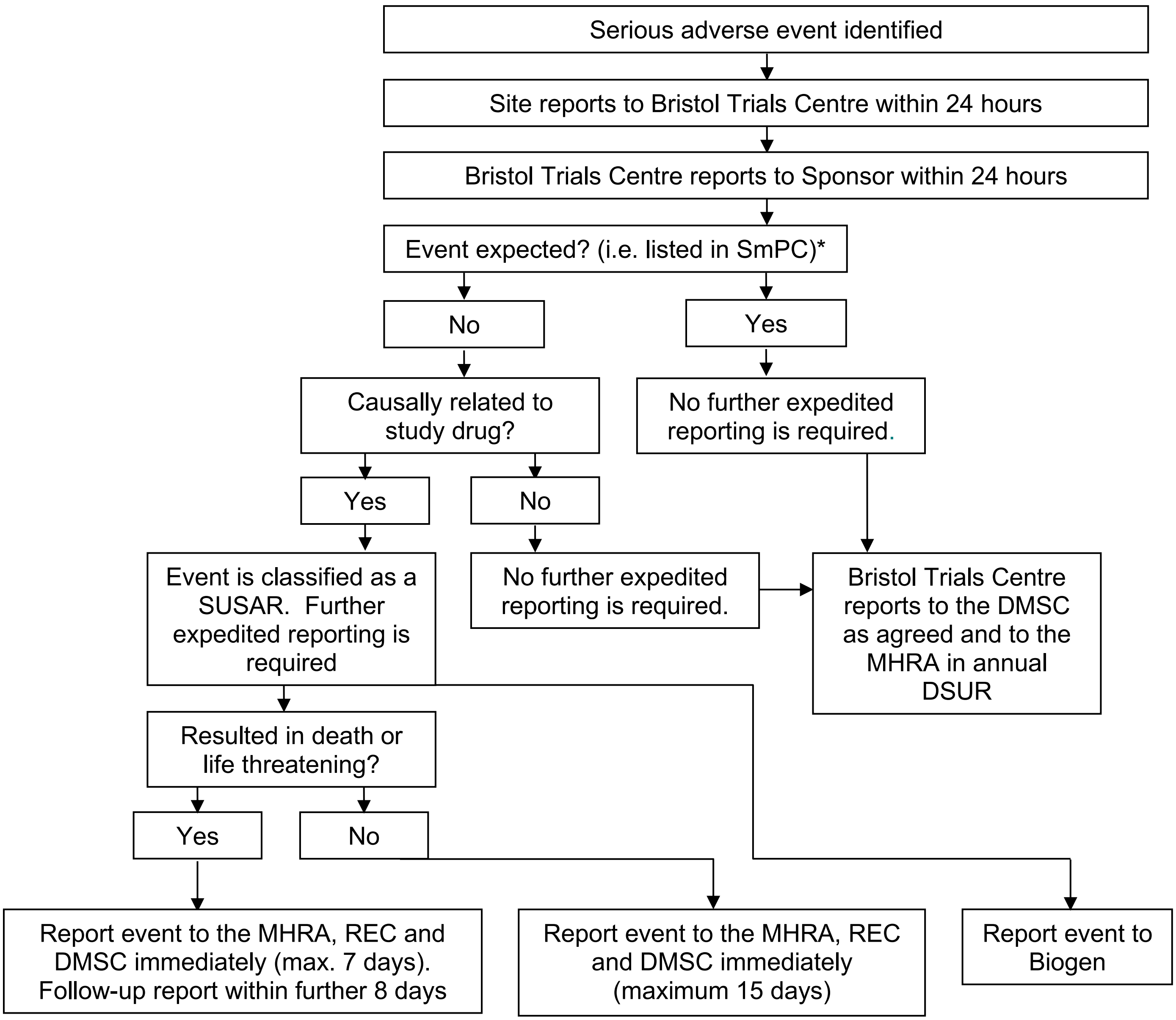

***NB** – if the event was fatal or life threatening, should follow the same reporting procedure as for unexpected events.

Figure 3 Serious adverse event reporting flow chart

10. Ethical considerations

10.1 Review by an NHS Research Ethics Committee

The research will be performed subject to a favourable opinion from an NHS REC and Health Research Authority (HRA) approval, including any provisions of a non-NHS site assessment form. Ethics review of the protocol for the trial and other trial related essential documents (e.g. PIL and consent form) will be carried out by a UK NHS REC. Any subsequent amendments to these documents will be submitted to the REC and HRA for approval prior to implementation.

10.2 Risks and anticipated benefits

For patients who are not eligible for adalimumab under NICE guidance, the main benefit of participation is the provision of adalimumab for the period of the TRI and, if classified as a responder conditional randomization, subsequently in the RCT. For patients who are eligible for adalimumab under NICE guidance, the main benefit will be knowing more accurately whether they are responders or not and further contributing to the UK cohort of safety and tolerability (which is being maintained for the benefit of all patients being treated with adalimumab).

For patients who are eligible for adalimumab under NICE guidance, the main risk of participation is potential permanent damage from ANIU if a participant is allocated to placebo after being classified as a responder. However, the risk is low as patients with the highest risk of deterioration (e.g. patients with Behcet's disease) will not be included in the study and follow-up visits are frequent enough that treatment failure should be identified before any permanent damage occurs. For all participants, there is also a potential risk of a serious adverse reaction from adalimumab, although such events are rare.

10.2.1 COVID-19 risk/benefit analysis

In 2020 the COVID-19 pandemic caused significant disruption to the NHS and the provision of non-COVID-19 clinical research. See Table 3 for a risk benefit analysis of the ASTUTE trial in relation to COVID-19.

Table 3 COVID-19 risk benefit analysis

Risk	Benefit
IMP supply to participants	
The risk is that participants will not receive their IMP or receive it late due to Sciensus, formerly called Healthcare at Home (HaH), being unable to deliver the IMP due to local lock downs.<br><br>We do not anticipate this will be a problem as Sciensus deliver Imraldi as part of usual NHS care and during the first wave of the pandemic, this service continued as normal.	The benefit of the design of the trial is that the IMP will be delivered to participant's homes by Sciensus, rather than participants collecting the IMP from the hospital pharmacies. Therefore, participants will not need to wait in the hospital for longer than their appointment time for their IMP to be dispensed by the hospital clinical trials pharmacy, which can sometimes delay patients from being able to leave the hospital.
Patient on immunosuppressants	

The risk is the concern that patients on immunosuppressants are at a greater risk if they become infected by COVID-19.<br><br>Based on the first wave of the pandemic, there does not appear to be any additional risk for individual's on immunosuppressants when adopting appropriate "hands, face, space" behaviours.<br><br>There is also no additional risk to participants of having to attend hospital appointments for the research, as the trial visit schedule has been designed to reflect usual care in this population. Care for patients who are eligible for the trial is now being provided face-to-face in a safe clinic environment (as before the pandemic but with extra COVID-19 precautions).	Many of the patients who will be recruited into the ASTUTE trial will already be taking another form of immunosuppressant. Therefore, participating in the trial will not put them at any additional risk from COVID-19. A benefit of the trial is that it may reduce the use of corticosteroids in these participants.<br><br>This was a commissioned call highlighting the importance of this research. Trials are an important part of providing the best possible care to participants.
Capacity of the research team to conduct the trial	
The risk is that if research staff are re-deployed to front line services, this will impact the running of the trial and, in particular, prompt identification of adverse events.<br>Based on the first wave of the pandemic, trusts where recruitment had to be stopped due to capacity were nevertheless able to ensure that the reduced research teams focused their efforts on maintaining safety in the trial participants. The safety of participants will remain a top priority even if some research staff are redeployed.<br>A risk to the trial is that recruitment proceeds more slowly or be paused at some sites.	The recently released NIHR guidance for a second wave of the pandemic highlighted that "NIHR funded research staff should not be deployed to front line duties except in exceptional circumstances" [62]. This guidance emphasises the importance of continuing to support non-COVID-19 research.<br><br>As clinical care for sight-threatening uveitis will not pause during a second wave, it is less likely that recruitment will be paused as this trial reflects standard of care.

10.3 Informing potential study participants of possible benefits and known risks

Information about possible benefits and risks of participation will be described in the PIL.

10.4 Obtaining informed consent from participants

All participants will be required to give written informed consent. This process, including the information about the trial given to patients in advance of recruitment, is described above in section 6.8.

The PI or members of the team delegated by the PI will be responsible for obtaining informed consent. The consent process will be described in detail in the Trial Manual. Research

personnel authorised to obtain consent will be recorded on the Delegation of Responsibilities Log. All individuals obtaining informed consent will have received GCP training. Potential study subjects will be fully apprised of potential risks and benefits of study participation and will be provided with detailed study information prior to written informed consent being sought.

10.5 Co-enrolment

Subject to agreement with the Chief Investigator, a participant may be co-enrolled to a non-intervention study as well as to the ASTUTE trial. A participant must not be co-enrolled to another intervention study.

11. Research governance

This study will be conducted in accordance with:

- The Medicine for Human Use (Clinical Trial) Regulations 2004
- Good Clinical Practice (GCP) guidelines
- UK Policy Framework for Health and Social Care Research
- European Union Directive 2001/20/EC on clinical trials

11.1 Sponsor approval

Any amendments to the trial documents must be approved by the sponsor prior to submission to the REC, HRA and MHRA (if applicable).

11.2 NHS confirmation of capacity and capability

Confirmation of capacity and capability is required prior to the start of the trial at each participating site.

Any amendments to the trial documents approved by the REC, HRA and MHRA (if applicable) will be submitted to participating sites for information and implementation, as required.

11.3 Investigators' responsibilities

Investigators will be required to ensure that local research approvals have been obtained and that any contractual agreements required have been signed off by all parties before recruiting any participant. Investigators will be required to ensure compliance to the protocol and study manual and with completion of the CRFs. Investigators will be required to allow access to study documentation or source data on request for monitoring visits and audits performed by the Sponsor or BTC or any regulatory authorities.

Investigators will be required to read, acknowledge and inform their trial team of any amendments to the trial documents approved by the REC, HRA and MHRA (if applicable) that they receive and ensure that the changes are complied with.

11.4 Monitoring by sponsor

The study will be monitored and audited in accordance with University Hospitals Bristol’s Monitoring and Oversight of Research Activity SOP, which is consistent with the UK Policy Framework for Health and Social Care Research and the Medicines for Human Use (Clinical Trials) Regulations 2004. All study related documents will be made available on request for monitoring and audit by the sponsor (or BTC if they have been delegated to monitor see 8.2.2), the relevant REC and for inspection by the HRA, MHRA or other licensing bodies. A monitoring plan will be prepared by the Sponsor.

11.5 Indemnity

This is an NHS-sponsored research study. For NHS sponsored research if there is negligent harm during the clinical trial when the NHS body owes a duty of care to the person harmed, NHS Indemnity covers NHS staff, medical academic staff with honorary contracts, and those conducting the trial. NHS Indemnity does not offer no-fault compensation and is unable to agree in advance to pay compensation for non-negligent harm. Ex-gratia payments may be considered in the case of a claim.

11.6 Clinical Trial Authorisation

Imraldi™ is classed as an investigational medicinal product and a Clinical Trial Authorisation from the MHRA must be in place before starting the trial.

12. Data protection and participant confidentiality

12.1 Data protection

Data will be collected and retained in accordance with the UK Data Protection Act 2018 and General Data Protection Regulation (GDPR) 2016.

12.2 Data handling, storage and sharing

12.2.1 Data handling

The ASTUTE study team will provide the Sponsor with a Data Management Plan prior to the study opening to recruitment.

Data will be entered into a purpose-designed server database hosted on the NHS network. Information capable of identifying individuals and the nature of treatment received will be held in the database with passwords restricted to ASTUTE study staff at the participating site and the co-ordinating centre. Information capable of identifying participants will not be made available in any form to those outside the study. The database and randomisation system will be designed so as to protect patient information in line with data protection legislation. Trial staff will ensure that the participants' anonymity is maintained through protective and secure handling and storage of patient information at participating sites and in accordance with ethics approval. All documents will be stored securely and only accessible by trial staff and authorised personnel. Data will be collected and retained in accordance with data protection legislation.

Access to the database will be via a secure password-protected web-interface. Study data transferred electronically to the University of Bristol network for statistical analyses will be pseudonymised and transferred via a secure network. The participants will be identified using their name and unique study identifier on the secure NHS hosted database.
Data will be entered promptly and data validation and cleaning will be carried out throughout the trial. The trial manual will cover database use, data validation and data cleaning. The manual will be available and regularly maintained.

12.2.2 Data storage

All study documentation will be retained in a secure location during the conduct of the study and for 15 years after the end of the study, when all patient identifiable paper records will be destroyed by confidential means. Where trial related information is documented in the medical records, these records will be identified by a label bearing the name and duration of the trial and clearly stating the 'do not destroy before' date. Where electronic records are in use, local site

policy will be followed. In compliance with the MRC Policy on Data Sharing, relevant 'meta'-data about the trial and the full dataset, but without any participant identifiers other than the unique participant identifier, will be held indefinitely (University of Bristol server).

12.2.3 Data sharing

Data will not be made available for sharing until after publication of the main results of the study. Thereafter, anonymised individual patient data will be made available for secondary research, conditional on assurance from the secondary researcher that the proposed use of the data is compliant with the MRC Policy on Data Sharing regarding scientific quality, ethical requirements and value for money. A minimum requirement with respect to scientific quality will be a publicly available pre-specified protocol describing the purpose, methods and analysis of the secondary research, e.g. a protocol for a Cochrane systematic review. The second file containing patient identifiers would be retained on a secure NHS server for 15 years for record linkage or a similar purpose, subject to confirmation that the secondary research protocol has been approved by a UK REC or other similar, approved ethics review body. Patient identifiers would not be passed on to any third party.

13. Dissemination of findings

The findings will be disseminated by usual academic channels, i.e. presentation at international meetings, as well as by peer-reviewed publications and through patient organisations and newsletters to patients, where available.

14. References

1. Jaffe, G.J., et al., *Adalimumab in Patients With Active, Non-infectious Uveitis Requiring High-dose Corticosteroids: the VISUAL-1 Trial*, in *Annals of the Rheumatic Diseases*. 2015. p. 894-850.
2. Frost, N.A., et al., *Development of a questionnaire for measurement of vision-related quality of life.* Ophthalmic Epidemiol, 1998. **5**(4): p. 185-210.
3. Group, E., *EuroQol-a new facility for the measurement of health-related quality of life.* Health Policy, 1990. **16**(3): p. 199-208.
4. Nguyen, Q.D., et al., *Adalimumab for prevention of uveitic flare in patients with inactive non-infectious uveitis controlled by corticosteroids (VISUAL II): a multicentre, double-masked, randomised, placebo-controlled phase 3 trial.* Lancet, 2016. **388**(10050): p. 1183-92.
5. Organization, W.H. *The World Health Report, (2010) Global Data on Visual Impairments*. 04/12/2019]; Available from: http://www.who.int/blindness/GLOBALDATAFINALforweb.pdf.
6. Nussenblatt, R.B., *The natural history of uveitis.* Int Ophthalmol, 1990. **14**(5-6): p. 303-8.
7. Bodaghi, B., et al., *Chronic severe uveitis: etiology and visual outcome in 927 patients from a single center.* Medicine (Baltimore), 2001. **80**(4): p. 263-70.
8. Rothova, A., et al., *Uveitis and systemic disease.* Br J Ophthalmol, 1992. **76**(3): p. 137-41.
9. Darrell, R.W., H.P. Wagener, and L.T. Kurland, *Epidemiology of uveitis. Incidence and prevalence in a small urban community.* Arch Ophthalmol, 1962. **68**: p. 502-14.
10. Suttorp-Schulten, M.S. and A. Rothova, *The possible impact of uveitis in blindness: a literature survey.* Br J Ophthalmol, 1996. **80**(9): p. 844-8.
11. Jabs, D.A., et al., *Standardization of uveitis nomenclature for reporting clinical data. Results of the First International Workshop.* Am J Ophthalmol, 2005. **140**(3): p. 509-16.

12. Agarwal, R.K., P.B. Silver, and R.R. Caspi, *Rodent models of experimental autoimmune uveitis.* Methods Mol Biol, 2012. **900**: p. 443-69.
13. Kilmartin, D.J., et al., *Immunogenetics and clinical phenotype of sympathetic ophthalmia in British and Irish patients.* Br J Ophthalmol, 2001. **85**(3): p. 281-6.
14. Islam, S.M., et al., *HLA class II genes in Vogt-Koyanagi-Harada disease.* Invest Ophthalmol Vis Sci, 1994. **35**(11): p. 3890-6.
15. Gocho, K., I. Kondo, and K. Yamaki, *Identification of autoreactive T cells in Vogt-Koyanagi-Harada disease.* Invest Ophthalmol Vis Sci, 2001. **42**(9): p. 2004-9.
16. Sugita, S., et al., *Ocular infiltrating CD4+ T cells from patients with Vogt-Koyanagi-Harada disease recognize human melanocyte antigens.* Invest Ophthalmol Vis Sci, 2006. **47**(6): p. 2547-54.
17. Todd, J.A., J.I. Bell, and H.O. McDevitt, *HLA-DQ beta gene contributes to susceptibility and resistance to insulin-dependent diabetes mellitus.* Nature, 1987. **329**(6140): p. 599-604.
18. Anderson, A.C., et al., *High frequency of autoreactive myelin proteolipid protein-specific T cells in the periphery of naive mice: mechanisms of selection of the self-reactive repertoire.* J Exp Med, 2000. **191**(5): p. 761-70.
19. Luger, D., et al., *Either a Th17 or a Th1 effector response can drive autoimmunity: conditions of disease induction affect dominant effector category.* J Exp Med, 2008. **205**(4): p. 799-810.
20. Peters, A., et al., *Th17 cells induce ectopic lymphoid follicles in central nervous system tissue inflammation.* Immunity, 2011. **35**(6): p. 986-96.
21. Zhou, R., et al., *The living eye "disarms" uncommitted autoreactive T cells by converting them to Foxp3(+) regulatory cells following local antigen recognition.* J Immunol, 2012. **188**(4): p. 1742-50.
22. Dick, A.D., et al., *The role of tumour necrosis factor (TNF-alpha) in experimental autoimmune uveoretinitis (EAU).* Prog Retin Eye Res, 2004. **23**(6): p. 617-37.
23. McMenamin, P.G., et al., *Ultrastructural pathology of experimental autoimmune uveitis in the rat.* Autoimmunity, 1993. **16**(2): p. 83-93.
24. Papotto, P.H., et al., *Immunotherapeutic strategies in autoimmune uveitis.* Autoimmun Rev, 2014. **13**(9): p. 909-16.
25. Dick, A.D., et al., *Inhibition of tumor necrosis factor activity minimizes target organ damage in experimental autoimmune uveoretinitis despite quantitatively normal activated T cell traffic to the retina.* Eur J Immunol, 1996. **26**(5): p. 1018-25.
26. Santos Lacomba, M., et al., *Aqueous humor and serum tumor necrosis factor-alpha in clinical uveitis.* Ophthalmic Res, 2001. **33**(5): p. 251-5.
27. Valentincic, N.V., et al., *Intraocular and serum cytokine profiles in patients with intermediate uveitis.* Mol Vis, 2011. **17**: p. 2003-10.
28. Murphy, C.C., et al., *Systemic CD4(+) T cell phenotype and activation status in intermediate uveitis.* Br J Ophthalmol, 2004. **88**(3): p. 412-6.
29. Schonermarck, U., E. Csernok, and W.L. Gross, *Pathogenesis of anti-neutrophil cytoplasmic antibody-associated vasculitis: challenges and solutions 2014.* Nephrol Dial Transplant, 2015. **30 Suppl 1**: p. i46-52.
30. Gritz, D.C. and I.G. Wong, *Incidence and prevalence of uveitis in Northern California; the Northern California Epidemiology of Uveitis Study.* Ophthalmology, 2004. **111**(3): p. 491-500; discussion 500.
31. Jabs, D., et al., *Guidelines for the use of immunosuppressive drugs in patients with ocular inflammatory disorders: recommendations of an expert panel.* Am J Ophthalmol, 2000. **130**(4): p. 492-513.

32. Castiblanco, C. and C.S. Foster, *Review of Systemic Immunosuppression for Autoimmune Uveitis.* Ophthalmol Ther, 2014. **3**(1-2): p. 17-36.
33. Daniel, E., et al., *Mycophenolate mofetil for ocular inflammation.* Am J Ophthalmol, 2010. **149**(3): p. 423-32 e1-2.
34. Pasadhika, S., et al., *Azathioprine for ocular inflammatory diseases.* Am J Ophthalmol, 2009. **148**(4): p. 500-509 e2.
35. Hogan, A.C., et al., *Long-term efficacy and tolerance of tacrolimus for the treatment of uveitis.* Ophthalmology, 2007. **114**(5): p. 1000-6.
36. Nguyen, Q.D., et al., *A cross-sectional study of the current treatment patterns in noninfectious uveitis among specialists in the United States.* Ophthalmology, 2011. **118**(1): p. 184-90.
37. Stanbury, R.M. and E.M. Graham, *Systemic corticosteroid therapy--side effects and their management.* Br J Ophthalmol, 1998. **82**(6): p. 704-8.
38. Tundia, N., et al., *Cost of complications for patients with non ‐ infectious non ‐ anterior uveitis.*, in *European Association for Vision and Eye Research Conference*. 2014, Wiley.
39. Qian, Y., et al., *Depression and visual functioning in patients with ocular inflammatory disease.* Am J Ophthalmol, 2012. **153**(2): p. 370-378 e2.
40. Naik, R.K., et al., *Normative comparison of patient-reported outcomes in patients with noninfectious uveitis.* JAMA Ophthalmol, 2013. **131**(2): p. 219-25.
41. Murphy, C.C., et al., *Quality of life and visual function in patients with intermediate uveitis.* Br J Ophthalmol, 2005. **89**(9): p. 1161-5.
42. Jalil, A., et al., *Vision-related quality of life and employment status in patients with uveitis of working age: a prospective study.* Ocul Immunol Inflamm, 2012. **20**(4): p. 262-5.
43. Carnahan, M.C. and D.A. Goldstein, *Ocular complications of topical, peri-ocular, and systemic corticosteroids.* Curr Opin Ophthalmol, 2000. **11**(6): p. 478-83.
44. Multicenter Uveitis Steroid Treatment Trial Research, G., et al., *Randomized comparison of systemic anti-inflammatory therapy versus fluocinolone acetonide implant for intermediate, posterior, and panuveitis: the multicenter uveitis steroid treatment trial.* Ophthalmology, 2011. **118**(10): p. 1916-26.
45. Tomkins-Netzer, O., et al., *Long-term clinical outcome and causes of vision loss in patients with uveitis.* Ophthalmology, 2014. **121**(12): p. 2387-92.
46. Mangione, C.M., et al., *Development of the 25-item National Eye Institute Visual Function Questionnaire.* Arch Ophthalmol, 2001. **119**(7): p. 1050-8.
47. Ophthalmology, N.E.S.C.R.G.f.S. *Clinical Commissioning Policy: Infliximab (Remicade) and Adalimumab (Humira) As Anti-TNF Treatment Options For Adult Patients with Severe Refractory Uveitis*. 2015 04/12/2019]; Available from: https://www.engage.england.nhs.uk/consultation/specialised-services-consultation/user_uploads/uveitis-adults-policy.pdf.
48. RNIB, L.A. *The economic impact of partial sight and blindness in the UK adult population*. 2009 04/12/2019]; Available from: http://www.rnib.org.uk/sites/default/files/FSUK_Report.pdf.
49. Levy-Clarke, G., et al., *Expert panel recommendations for the use of anti-tumor necrosis factor biologic agents in patients with ocular inflammatory disorders.* Ophthalmology, 2014. **121**(3): p. 785-96 e3.
50. Cordero-Coma, M., T. Yilmaz, and S. Onal, *Systematic review of anti-tumor necrosis factor-alpha therapy for treatment of immune-mediated uveitis.* Ocul Immunol Inflamm, 2013. **21**(1): p. 19-27.
51. Pasadhika, S. and J.T. Rosenbaum, *Update on the use of systemic biologic agents in the treatment of noninfectious uveitis.* Biologics, 2014. **8**: p. 67-81.

52. Excellence, N.I.f.H.a.C. *Adalimumab and dexamethasone for treating non-infectious uveitis*. 2017 04/12/2019]; Available from: Nice.org.uk/guidance/ta460.
53. Foss, A.J., et al., *Comparing different dosing regimens of bevacizumab in the treatment of neovascular macular degeneration: study protocol for a randomised controlled trial.* Trials, 2015. **16**: p. 85.
54. Higgins, J., et al. *Revised Cochrane risk-of-bias tool for randomized trials (RoB 2)*. 04/12/2019]; Available from: https://sites.google.com/site/riskofbiastool/welcome/rob-2-0-tool.
55. Suhler, E.B., et al., *Adalimumab therapy for refractory uveitis: results of a multicentre, open-label, prospective trial.* Br J Ophthalmol, 2013. **97**(4): p. 481-6.
56. Suhler, E.B., et al., *A prospective trial of infliximab therapy for refractory uveitis: preliminary safety and efficacy outcomes.* Arch Ophthalmol, 2005. **123**(7): p. 903-12.
57. Bristol Trials Centre, U.o.B. *IMP-Track User Manual*. 2018; Available from: https://cpb-eu-w2.wpmucdn.com/blogs.bristol.ac.uk/dist/4/628/files/2020/07/imp-track-user-manual-v10.pdf.
58. Trusko, B., et al., *The Standardization of Uveitis Nomenclature (SUN) Project. Development of a clinical evidence base utilizing informatics tools and techniques.* Methods Inf Med, 2013. **52**(3): p. 259-65, S1-6.
59. Okhravi, N. and S. Lightman, *Cystoid macular edema in uveitis.* Ocul Immunol Inflamm, 2003. **11**(1): p. 29-38.
60. Excellence, N.I.f.H.a.C., *Guide to the Methods of Technology Appraisal*, in *Process and Methods Guides*. 2013: London.
61. Dolan, P., et al., *A Social Tariff for EuroQol: Results from a UK General Population Survey.* University of York: Center for Health Economics, 1995. **138**.
62. NIHR. 2020; Available from: https://www.nihr.ac.uk/news/nihr-issues-guidance-to-ensure-research-protected-in-a-second-wave/25858.

15. Amendments to protocol

Amendment number (i.e. REC and/or MHRA	Previous version	Previous date	New version	New date	Brief summary of change	Date of ethical approval (or NA if non-substantial)
N/A (pre-approvals) Changes made following initial REC review.	2.0	11/03/2020	3.0	20/05/2020	Added ISRCTN number<br>5.6.2 Updated exclusion criteria<br>6.13 Added further detail about expense claims	03/06/2020
N/A (pre-approvals) Changes made following initial MHRA review.	3.0	20/05/2020	4.0	15/10/2020	5.7 Clarified live vaccines cannot be administered<br>10.2.1 Added COVID-19 risk/benefit analysis	26/10/2020
1	4.0	15/10/2020	5.0	26/10/2020	5.6.1 Clarified definition of active ANIU in inclusion criteria<br>5.6.2 Updated exclusion criteria to add toxoplasmosis chorioretinitis, excluded patients who are steroid-dependent for a condition other than ANIU and updated the conditions under which patients are excluded based on previous steroid treatments.<br>5.7 Clarified pharmacy arrangements<br>5.8.1 Clarified TF criteria<br>6.3 Changed 8-week TRI visit to a telephone call.<br>6.3.1 Clarified responder criteria<br>Figure 1 corrected minor errors in schema<br>Table 1 updated TRI assessments regarding week 8 phone call.	17/11/2020

					Table 2 Updated projected recruitment figures.<br>Updated link to reference 57.	
3	5.0	26/10/2020	6.0	11/11/2021	Page 2 - Addition of a new trial manager;<br>Pages 8, 14 & 22 - Addition of BCVA as a secondary outcome;<br>Throughout - Change of “Healthcare at Home Ltd” name to “Sciensus”;<br>Page 22 - Clarification of timepoints for patient-reported side effects to match Table 1;<br>Page 26 - Correction of typo on Table 1 caption, stating RCT visit windows as ±7 days;<br>Page 34 - Clarification of SAE reporting window;<br>Page 34 & Appendix 1 - Updated SmPC;<br>Page 26 - Update to the 4 week window between screening visit and enrolment, allowing it to be extended to 6 weeks in exceptional circumstances with approval of the PI and trial team.	14/12/2021
6	6.0	11/11/2021	7.0	25/04/2022	Page 2 – Addition of a new trial manager;<br>Page 7 – Addition of the exclusion criterion of an epiretinal membrane likely to prevent an eye meeting response criterion at 16 weeks of central macular thickness <320um;<br>Page 19 – Addition of above exclusion criterion<br>Page 21 – Error in SmPC date amended.	01/06/2022

7	7.0	21/02/2022	8.0	29/07/2022	Throughout – changes of reference to BTC CTEU to just BTC in line with trials centre merger.<br>Page 1&2 -change of BTC address. Addition of Sponsor and funder no. Removal of trial previous managers and update of phone numbers.<br>Page 20 – clarification on taking delayed doses in TRI and RCT. Clarify that RCT injection starts 1 week after randomisation<br>Page 25/26 – updated detail about imaging requirements and windows.	17/08/2022
9	8.0	29/07/2022	9.0	31/10/2023	Page 2 – updated co-applicant details<br>Page 20 – added detail on managing intraocular surgery<br>Page 22 – added information about impact of intraocular surgery on TF criteria<br>Throughout correction of sponsor's name from UHB to UBHW<br>Throughout – clarifications on processes	04/12/2023
15	9.0	31/10/2023	10.0	12/07/2024	The protocol has been updated throughout to extend the maximum duration of participation and to decrease the minimum duration.<br>Section 6.3.1 has been updated to clarify the responder criteria.	15/08/2024
16	10.0	12/07/2024	11.0	10/09/2024	The protocol has been updated throughout to incorporate the open-label extension.<br>The study team has been updated.	

17	11.0	10/09/2024	12.0	31/03/2025	Updated protocol to correct minor typo on p27 for clarity as previous protocol contained a double negative. Addition of clarity around safety reporting period for the OLE trial as it was not clear from the previous protocol version.	N/A (HRA confirmation 22/05/2025)
18	12.0	31/03/2025	13.0	22/09/2025	Update of co-applicant contact details. Minor administrative change and correction of typos. Clarification updated about length of OLE follow up and confirmation of when last patient visits will occur for each patient. Removal of appendix 1 (SmPC dated 16-August-2021), Update to named Safety Reporting SOP.	

The ASTUTE Trial is funded by the NIHR HTA Programme (project reference 16/24/09). The views expressed are those of the author(s) and not necessarily those of the NIHR or the Department of Health and Social Care.
