# Supplementary material for: Adalimumab versus placebo as add-on to standard therapy for non-infectious uveitis in the United Kingdom: the ASTUTE randomised controlled trial with treatment run-in
Source: eClinicalMedicine. 2026 Oct 8;101:104240. doi: 10.1016/j.eclinm.2026.104240 (PMC13677017; doi:10.1016/j.eclinm.2026.104240)
Supplement: Supplementary file 2 [file mmc2.pdf]

ASTUTE analyses supplementary file

Table of contents

1 Trial methods – additional information2

1.1 Details of eligibility2

1.2 Details of over-labelling of the pen devices and their distribution4

1.3 Details of switching and further TRI and RCT phases4

1.4 Details of the cost-effectiveness analysis5

2 Supplementary tables and figures11

Trial methods – additional information

Details of eligibility

The target population is adults with active or controlled non-infectious uveitis (NIU). We define NIU in relation to anatomic descriptors of intermediate, posterior and panuveitis, because these features cause most sight loss. These anatomic descriptors allow the results of the trial to be applied across specific diseases, increasing applicability of the results to UK patients with NIU who do not respond to usual care. The frequency of different specific diseases in the trial population was expected to be similar to other trials: ocular autoimmune (idiopathic) 60%; Birdshot Uveitis 30%; multifocal choroiditis 4%; PIC 5%; Vogt Kayanagi Harada 1%; and sarcoidosis where the mechanism is autoimmune and TNFα is a critical cytokine.

Inclusion criteria

A participant could take part in the study if **ALL** of the following applied:

1. Participant is aged 18 years or over;
2. Participant has: (a) active sight threatening NIU (active inflammatory chorioretinal lesions OR abnormal central macular thickness (CMT) OR evidence of retinal vasculitis OR vitreous haze >0.5) and is being prescribed (already taking or being started on, if newly presenting with ANIU) oral prednisolone >5 mg/day; OR (b) has controlled ANIU and is being prescribed oral prednisolone >5 mg/day.
3. Women must have a negative pregnancy test and be willing to use effective contraception* for the duration of the participation in the trial and for 5 months after, or be surgically sterile or post-menopausal for >12 months.
4. Participant has to be able to provide informed consent.

* This includes: progestogen-only oral hormonal contraception, where inhibition of ovulation is not the primary mode of action, male or female condom with or without spermicide cap, diaphragm or sponge with spermicide, combined (oestrogen and progestogen containing) hormonal contraception associated with inhibition of ovulation (oral, intravaginal or transdermal), progestogen-only hormonal contraception associated with inhibition of ovulation: (oral, injectable, implantable), intrauterine device, intrauterine hormone-releasing system, bilateral tubal occlusion, vasectomised partner, sexual abstinence.

NB. A pregnancy test only needed to be repeated if there was reason to suspect the participant had become pregnant. If a participant became pregnant during the trial the treating clinician was required to discuss the options with the patient on whether or not to continue in the trial, referring to the current SmPC for guidance on pregnancy and Imraldi™.

There were no special precautions/contraceptive requirements for male participants with female partners of child-bearing potential.

1 *Exclusion criteria*

A participant could not take part in the study if any of the following applied:

1. Participant has controlled NIU and is maintained on oral prednisolone ≤5 mg/day at the time of screening;
2. Participant has systemic disease (whether associated with NIU or not) that is being treated with steroids and requires >5 mg/day oral prednisolone.
3. Participant has untreated or active tuberculosis;
4. Participant has severe infection, sepsis or opportunistic infection;
5. Participant has uncontrolled glaucoma;
6. Participant has multiple sclerosis;
7. Participant is HIV positive;
8. Participant has hepatitis B or hepatitis C;
9. Participant has syphilis;
10. Participant has Lyme disease;
11. Participant has Behcet's disease;
12. Participant has toxoplasmosis chorioretinitis;
13. Participant has heart failure (NYHA III/IV);
14. Participant has been diagnosed with cancer <5 years ago;
15. Participant is undergoing monitoring for recurrence of cancer / tumour growth where their oncologist has concern that a TNF α inhibitor would be contraindicated;
16. Participant is taking another biologic drug;
17. Participant has taken an anti-TNF drug within the previous 90 days (anakinra and abatacept are contraindicated);
18. Participant has had an Iluvien® implant within the previous 18 months and has controlled ANIU, or has had an Iluvien® implant within the previous 12 weeks regardless of whether ANIU is active or controlled;
19. Participant has had an Ozurdex® implant, or an intravitreal steroid injection, or periocular steroid within the previous 12 weeks regardless of whether ANIU is active or controlled;
20. Participant is pregnant;
21. Participant has a known allergy or hypersensitivity to adalimumab or any of its excipients (refer to summary of product characteristics (SmPC) for a list of excipients);
22. Participant is taking part in another interventional study;

23. Participant has an epiretinal membrane likely to prevent an eye meeting the response criterion of central macular thickness <320um at 16 weeks.

Details of over-labelling of the pen devices and their distribution

All pen devices were over-labelled and assigned serial numbers by a NHS manufacturing pharmacy (University Hospital of Liverpool) to identify them as investigational medicinal product (IMP). IMP was stored by the NHS pharmacy in accordance with the SmPC/dossier and delivered to a third-party homecare delivery company (Sciensus Ltd, Burton-on-Trent) in batches, maintaining a cold chain. Before participants started the TRI, Sciensus trained them on how to store and to self-administer injections. Sciensus dispensed the pen devices in accordance with prescriptions from ophthalmologists at participating hospitals and delivered the pen devices to participants at their specified addresses, maintaining the cold-chain.

Both the IMP and placebo were labelled and stored in accordance with Annex 13 and SmPC/dossier (Appendix 1) respectively. The contents of the label were approved by the MHRA and accounted for according to NHS pharmacy standards.

Details of switching and further TRI and RCT phases

It appeared perverse to us to invite participants to join the treatment run-in (TRI), prescribe open-label adalimumab for 16-weeks, assess whether participants had responded to adalimumab and then to randomise those who responded to adalimumab or placebo. In consultation with patients, we included a switching feature in the design of the study to address this situation.

In the event of a first TF in either or both eyes during the RCT, the participant was offered the opportunity to repeat the TRI. The second TRI proceeded in the same way as the first; if a participant responded at the 16-week visit, they re-started the RCT but with the alternative intervention, keeping all clinical and research staff masked to both the original and alternative allocations. If a participant experienced TF again, this process could be repeated a third time. A participant was withdrawn from the study after a third TF. Exceptionally, if TF was observed at a participant’s final assessment in the RCT before transitioning to the open-label extension (OLE), the participant was withdrawn from the study, i.e. was not offered the opportunity to join the OLE. Data collected during the additional TRI and RCT phases will be reported when the open-label extension concludes.

Details of the cost-effectiveness analysis

The aim of the economic analysis alongside the ASTUTE trial was to compare the cost-effectiveness and cost-utility of an additional 12 months of adalimumab (intervention) compared with stopping adalimumab after 16 weeks (comparator) when used to treat autoimmune non-infectious uveitis in either or both eyes in patients who responded to 16 weeks of adalimumab in the TRI period.[3] The analysis follows the National Institute for Health and Care Excellence (NICE) guidelines,[1] adopting the perspective of the UK National Health Service (NHS). The cost-utility analysis (which will combine EQ5D quality of life data with costs) will be reported separately. This current analysis focuses on the cost-effectiveness analysis, describing the incremental costs of avoiding a treatment failure at month 12 in the intervention and the comparator arms. The analysis included costs for participants from the randomisation to the first treatment failure, or transfer to the open-label extension.

We costed resource use after randomisation, including intervention injections (adalimumab/placebo), monitoring visits and clinical examinations, concomitant medications and treatment of adverse events and serious adverse events. To prevent unrelated resource use from overwhelming the impact of treatment, procedures, admissions, and consultations unrelated to either the study intervention or adverse events/serious adverse events identified by study investigators were excluded from the costing analysis. For each participant in the trial, we computed the total cost per participant by multiplying units of resource use by their unit cost and summing them up. Unit costs are presented in **Table ST1**. Assumptions used to perform the costing analysis are listed below. Costs incurred over 48 weeks were discounted at 3.5% per annum. Costs beyond 12 months (up to 128 weeks where data are available) are presented in **Table ST15** for information, but not combined with effectiveness data after 12 months

To account for differing follow-up times and the potential association between costs and time to first TF, we fitted a joint model comprising:

a) A longitudinal cost sub-model, in which repeated measures of total healthcare costs were analysed using a mixed-effects Gamma regression with a log link. The model included a patient-specific random intercept and random slope for time, and fixed effects for treatment group, time, treatment-by-time interaction, baseline age, baseline EQ-5D utility, and baseline better-seeing-eye visual acuity; and
b) A time-to-event sub-model for time to first TF, specified as a Weibull proportional hazards model. The log-hazard was modelled as a linear function of treatment and the same baseline covariates as above and included the patient-level random intercept from

the cost sub-model as a shared frailty term to induce correlation between the cost and survival processes.

Effectiveness was measured as the difference in the proportion of patients experiencing TF between the intervention and comparator arms. We calculated the incremental cost-effectiveness ratio (ICER) as the additional cost per TF avoided, which can be compared with a willingness-to-pay threshold for avoiding TF. We focus on the ICER at 12 months because beyond this time, the observed number of patients without TF declines markedly, so estimates of costs and effects rely heavily on extrapolation from the model and are therefore associated with substantial uncertainty. Uncertainty was quantified parametrically. STATA 18.0 was used to perform the health economic analysis.

Assumptions underpinning the costing analysis were as follows:

1) Intervention injections (adalimumab/placebo)
    - Adalimumab injections after randomisation were costed based on the actual prescriptions issued, rather than the planned prescriptions. The treatment was self-administered by participants at home, resulting in no administration costs to the NHS.
    - The cost of adalimumab was applied per 14 days, assuming 0.07142857 (=1/14) injections per day.
    - If the number of reported missed doses exceeded the number estimated from visit gaps, the number of IMPs taken was assumed to be zero.
    - One participant did not attend the 24-week visit but attended the following visit; we assumed they did not miss any IMP between the two visits.
    - The cost of the placebo was not considered, as from the NHS perspective, a placebo would not be administered in a routine clinical setting and would have no cost.
2) Monitoring visit and clinical examinations
    - 12-weekly follow-up visits were costed using consultation costs from the Department of Health reference costs, as per the trial protocol.
    - Clinical exams, including slit-lamp examination, direct ophthalmoscopy, optical coherence tomography (OCT), best corrected visual acuity (BCVA) and fundus colour imaging, are typically conducted as part of NHS ophthalmology consultations and therefore were not costed separately.
    - No cost was applied to visits that were scheduled but not attended, regardless of the reason for non-attendance.

- One ophthalmology visit was assigned to each unscheduled visit not recorded in the adverse event or serious adverse event forms.
- No cost was applied to Autofluorescence as these procedures were a part of normal outpatient monitoring visits (counted separately).

3) Concomitant medication
   - New concomitant medications reported on the New Medication forms and started after randomisation were included. This record includes new medications prescribed since the previous visit and those prescribed at this visit, excluding over-the-counter medications and unchanged medications from the previous visit.
   - The base-case analysis excluded over-the-counter medications because they were required to be excluded in the case report form and were not typically reimbursed by the NHS, including Adcal, Aspirin, Calcichew, Canesten Bifonazole, Cetirizine, Clotrimazole Cream, Clotrimazole Pessaries, Colecalciferol, Dermol, Famotidine, Ferrous Sulfate, Fexofenadine, Folic Acid, Gaviscon, Hypromellose, Ibuprofen, Maalox Plus, Omeprazole, Paracetamol, Phenergan, Sodium Hyaluronate, White Soft Paraffin. The base-case analysis also excluded medications unrelated to eye or the trial intervention, as determined by clinical review, including: Amitriptyline, Atorvastatin, Betamethasone sodium phosphate, Bisoprolol, Colchicine, Diazepam, Eplerenone, Estradiol, Estriol, Fluticasone furoate, Fucibet, Indapamide, Levothyroxine, Losartan, Metformin, Montelukast, Naseptin, Naproxen, Progesterone, Semaglutide, Sumatriptan, Tirzepatide, Umeclidinium, Vilanterol, Zopiclone. These medications would be included in sensitivity analyses.
   - Study case report forms recorded the generic name of each drug prescribed since the previous visit, along with the date started, ongoing status, date stopped, dose, dose unit, and route of administration (oral, subcutaneous, or other). These details were used to estimate the duration of medication use and associated costs relative to randomisation. Where only the brand name was reported, the unit cost was calculated using the available dose, route, and brand-specific pricing.
   - Medication unit costs were estimated based on drug name, dose, dose unit, and route, using data from the Prescription Cost Analysis – England – 2023/24,[4] assuming each prescription covers 28 days.
   - Where dose, dose unit, or route were unmatched in PCA data, the route of administration was prioritised, and a weighted average unit cost across all reported doses was applied, assuming participants more accurately recall the

route than the dose. For instance, when methotrexate was reported at a 25 mg dose via the oral route, where no 25 mg oral formulation was available in the PCA, we estimated the unit cost by calculating a weighted average across available oral doses (2 mg, 2.5 mg, 5 mg, 7.5 mg, and 10 mg).

- Medicines reported as administered via intravitreal injection (e.g., betamethasone, mydricaine, mydricaine+betamethasone, oxybuprocaine) were cross-checked against AE/SAE forms, as such procedures are not typically conducted in community settings.
- Vaccinations, including COVID-19 vaccines (e.g., Moderna), shingles vaccination and seasonal flu vaccines, were excluded from the costing analysis.
- Unit prices for concomitant medications were calculated using the weighted average of all prescriptions issued under each generic name. This approach reflects NHS prescribing patterns, where generic options are typically preferred and more frequently used.[5]
- Dosage forms such as paediatric formulations, suppositories, oral suspensions, liquids, drops, rectal solutions, effervescent tablets, and emulsions were excluded from the unit price calculation, as these formulations are not typically prescribed for the target population.
- The cost of oral prednisolone was estimated using the unit price of the 5 mg tablet, as this is the most commonly prescribed strength in NHS community settings (4,937,257 items in 2023/24). Doses below 5 mg were rounded up and costed as 5 mg; higher doses were calculated as integer multiples of the 5 mg unit. For example, a 20 mg prescription was costed as four 5 mg tablets.
- For symptomatic relief medicines (e.g., Gaviscon) and antiviral medicines (e.g., Acyclovir), where the reported stop date preceded the start date, we assumed a recall bias and imputed a treatment duration of 7 days, reflecting typical short-term usage for acute conditions.

4) Adverse events and serious adverse events
    - In collaboration with the ASTUTE trial clinicians, we included adverse events and serious adverse events that are related to the eye and have a plausible relationship to the study intervention.
    - To collect data on the resources that would be required to manage and treat the adverse events, we checked the descriptions of the treatments of adverse events as described in the relevant CRF forms. Where a description was not provided, we consulted clinicians on the treatment patients were likely to have received for their particular adverse events.

The healthcare resources were costed using the service and currency codes reported in the National Cost Collection for the NHS 23-24 and the Unit Costs of Health and Social Care 2024 Manual published by the Personal Social Services Research Unit. We costed primary care services, and hospital services and procedures, including imaging, day surgeries and laboratory tests.

- Medications were considered only in the concomitant medications analysis to avoid double-counting.
- For each trial participant, we calculated the total cost per participant by multiplying units of resource use by their unit cost and summing them up.

Table ST1 Unit costs

**Resource (units)**	**Cost**	**Source**
Adalimumab (per 40 mg dose)	£37.45	Cost per pre-filled pen containing 50 mg/ml for injection The trial supply cost for the adalimumab biosimilar Imraldi is<br>£74.90 excluding VAT for a twin-pack of Imraldi
Monitoring consultation (per consultation)	£140.01	Summary: outpatient attendances code for 130-ophthalmology service (total) in the National Schedule of NHS Costs Year 2023-24
Full blood count	£3.10	Directly accessed pathology services code for PATH05 haematology in the National Schedule of NHS Costs Year 2023-24
Liver function test	£3.10	Directly accessed pathology services code for PATH05 haematology in the National Schedule of NHS Costs Year 2023-24
Electrolyte profile	£3.10	Directly accessed pathology services code for PATH05 haematology in the National Schedule of NHS Costs Year 2023-24
Autofluorescence	£166.97	Outpatient procedure code for BZ88A Retinal Tomography, 19 years and over (130-ophthalmology service) in the National Schedule of NHS Costs Year 2023-24
Fundus fluorescein angiogram	£166.97	Outpatient procedure code for BZ88A Retinal Tomography, 19 years and over (130-ophthalmology service) in National Schedule of NHS Costs Year 2023-24
**Primary care – outpatient**		

GP (per surgery consultation lasting 10 minutes)	40.00	PSSRU 2024,[6] 73 pp. 9.4.2: Unit costs for a GP. Includes qualifications, but excludes direct staff costs
**Outpatient service**		
Clinical Haematology Service	£184.09	National schedule of NHS costs 2023/24.[7] Outpatient care – 330
Ear Nose and Throat Service	£163.38	National schedule of NHS costs 2023/24.[7] Outpatient care – 120
Rheumatology Service	£181.45	National schedule of NHS costs 2023/24.[7] Outpatient care – 410
**Outpatient procedures and imaging**		
Laser retinopexy	£313.89	National schedule of NHS costs 2023/24.[7] Admitted Patient Care – outpatient, BZ86B, Intermediate Vitreous Retinal Procedures, 19 years and over, with CC Score 0-1
Wireless Capsule Endoscopy	£136.40	National schedule of NHS costs 2023/24.[7] Admitted Patient Care – outpatient, FE50A, 19 years and over
Computerised Tomography Scan of One Area, with Post-Contrast Only	£105.43	National schedule of NHS costs 2023/24.[7] Outpatient Imaging, RD21A, 19 years and over for lung CT
Dexa Scan	£75.43	National schedule of NHS costs 2023/24.[7] Outpatient Imaging, RD50Z, 19 years and over for lung CT
Ultrasound Scan with duration of less than 20 minutes, without Contrast	£61.39	National schedule of NHS costs 2023/24.[7] Outpatient Imaging, RD40Z
(Barium swallow) Contrast Fluoroscopy Procedures with duration of less than 20 minutes	£166.73	National schedule of NHS costs 2023/24.[7] Outpatient Imaging, RD30Z
**Day surgery**		
Phacoemulsification Cataract Extraction and Lens Implant, with CC Score 0-1	£1,379.96	National schedule of NHS costs 2023/24.[7] Admitted Patient Care - day case, BZ34C

Abbreviations: GP, general practitioner; PSSRU, Personal Social Services Research Unit; NHS, National Health Service.

Supplementary tables and figures

Figure SF1 Flow of participants after treatment failure in RCT1

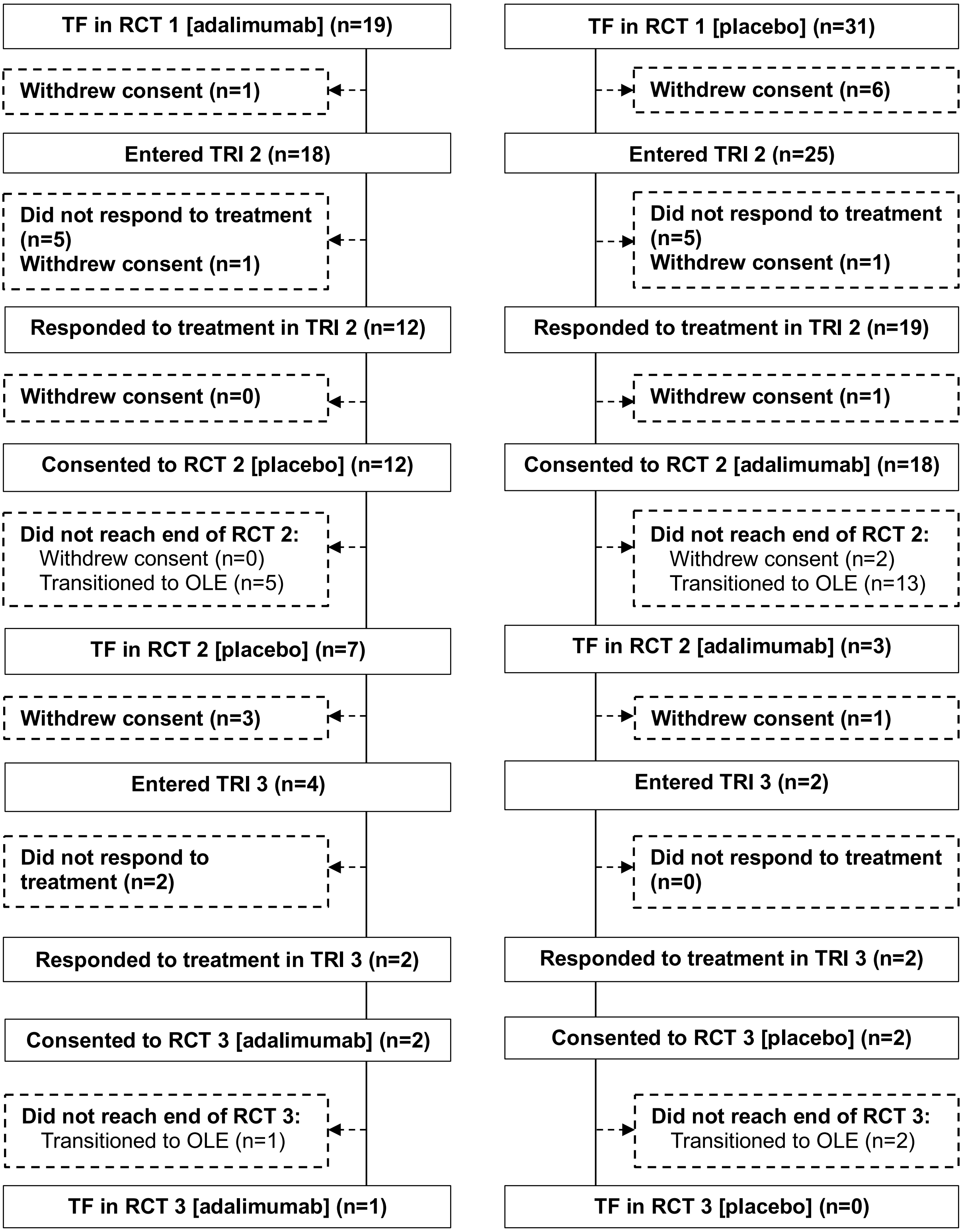

Table ST2 Details of participants who withdrew consent after randomisation

	Randomised to adalimumab (n=60)		Randomised to placebo (n=55)	
**Period**	**Withdrawn from trial (stopped both treatment and follow-up)**	**Withdrawn from treatment (stopped treatment but continued follow-up)**	**Withdrawn from trial (stopped both treatment and follow-up)**	**Withdrawn from treatment (stopped treatment but continued follow-up)**
**During RCT1**	**5**	**0**	**5**	**1**
**Reasons[a]**				
Patient decision	**1**	**0**	**4**	**1**
Does not want to take study drug	1	0	1	0
Did not give a reason	0	0	1	0
Other [b]	0	0	2	1
Clinician decision	**4**	**0**	**3**	**0**
Clinical reasons	2	0	1	0
Concern over adverse event	1	0	2	0
Other [c]	1	0	0	0
Admin reason	**0**	**0**	**0**	**0**

[a] Patients could withdraw for more than one reason

[b] Other reasons are "Following medical advice from Dermatologist and Ophthalmologist, "Patient does not feel she is improving" and "States he has felt unwell since starting the study".

[c] Other reason is "Advised by the Chief Investigator not to randomise as patient had cataract surgery".

Table ST3 Summary of adherence with trial procedures

Criteria	Randomised to adalimumab (n=60)	Randomised to placebo (n=55)
Participant met the trial eligibility criteria and were randomised	60 (100.0%)	54 (98.2%)
Participant received allocated intervention	59 (98.3%)	54 (98.2%)
Participant took at least 70% of trial medication in the first TRI and RCT[a]	59 (98.3%)	52 (94.5%)
**Participants met all criteria**	**58 (96.7%)**	**50 (90.9%)**

[a] Assumed all medication was missed if participant did not attend visit

Table ST4 Adherence of participants to timing of follow-up visits

	Number of in-window visits[a] / Number of due visits (%)	Mean days out of window	Number of in-window visits / Number of due visits (%)	Mean days out of window
**TRI**	**Adalimumab (n=202)**			
Week 4	155/193 (80.3%)	7.6		
Week 8	161/189 (85.2%)	7.1		
Week 16	145/185 (78.4%)	6.2		
**Overall**	**461/567 (81.3%)**	**6.9**		
**RCT**	**Randomised to adalimumab (n=60)**		**Randomised to placebo (n=55)**	
Week 12	52/56 (92.9%)	10.2	42/48 (87.5%)	11.0
Week 24	38/44 (86.4%)	7.4	28/34 (82.4%)	13.0
Week 36	34/38 (89.5%)	12.0	21/25 (84.0%)	17.2
Week 48	29/32 (90.6%)	9.0	13/15 (86.7%)	27.0
Week 64	25/26 (96.2%)	1.0	11/14 (78.6%)	22.0
Week 80	14/15 (93.3%)	9.0	8/9 (88.9%)	6.0
Week 96	9/9 (100.0%)	-	8/8 (100.0%)	-
Week 112	6/7 (85.7%)	6.0	6/6 (100.0%)	-
Week 128	4/4 (100.0%)	-	3/4 (75.0%)	1.0
**Overall**	**211/231 (91.3%)**	**8.9**	**140/163 (85.9%)**	**15.0**

[a] Visit windows were pre-specified

Table ST5 Adherence of participants to schedule of injections

	Number received all injections / Number of due visits (%)[a]	Number received all injections / Number of due visits (%)[a]
**TRI**	**Adalimumab (n=202)**	
Week 4	182/193 (94.3%)	
Week 8	178/189 (94.2%)	
Week 16	162/185 (87.6%)	
**Overall**	**522/567 (92.1%)**	
**RCT**	**Randomised to adalimumab (n=60)**	**Randomised to placebo (n=55)**
Week 12	46/56 (82.1%)	40/48 (83.3%)
Week 24	34/44 (77.3%)	27/34 (79.4%)
Week 36	35/38 (92.1%)	20/25 (80.0%)
Week 48	28/32 (87.5%)	13/15 (86.7%)
Week 64	23/26 (88.5%)	11/14 (78.6%)
Week 80	14/15 (93.3%)	8/9 (88.9%)
Week 96	6/9 (66.7%)	5/8 (62.5%)
Week 112	6/7 (85.7%)	5/6 (83.3%)
Week 128	3/4 (75.0%)	3/4 (75.0%)
**Overall**	**195/231 (84.4%)**	**132/163 (81.0%)**

[a] Patients received an injection every 2 weeks.

Table ST6 Additional baseline characteristics of randomised participants (assessed at TRI entry)

	Randomised to adalimumab (n=60)	Randomised to placebo (n=55)
**CLINICAL CHARACTERISTICS AND BLOOD TESTS**		
Heart rate (bpm); mean (SD)[a]	77.1 (12.2)	74.7 (11.5)
Diastolic blood pressure (mm Hg); mean (SD)	82.0 (10.0)	80.5 (9.7)
Systolic blood pressure (mm Hg); mean (SD)	137.7 (21.2)	134.4 (19.4)
Blood samples taken (yes)	60 (100.0%)	55 (100.0%)
WBC (x10^9 /L); median (IQR)	8.4 (6.5, 10.8)	8.5 (6.6, 10.6)
Lymphocytes (x10^9 /L); median (IQR)	1.3 (0.9, 2.1)	1.4 (0.9, 2.0)
Neutrophils (x10^9 /L); median (IQR)	6.0 (4.4, 8.7)	6.3 (4.3, 8.9)
Haematocrit (%); median (IQR)[b]	41.6 (40.0, 44.8)	42.8 (40.0, 46.2)
Platelets (x10^9 /L); mean (SD)	280.0 (63.0)	286.3 (72.5)
Hb (g /L); mean (SD)	141.5 (13.2)	143.6 (13.7)
MCV (fL); mean (SD)	90.3 (5.4)	90.9 (5.5)
Urea (mmol /L); median (IQR)[c]	4.8 (4.1, 6.1)	5.0 (4.3, 5.8)
Potassium (mmol /L); mean (SD)[d]	4.2 (0.4)	4.2 (0.5)
Sodium (mmol /L); mean (SD)	139.8 (1.8)	140.2 (1.9)
Chloride (mmol /L); mean (SD)[e]	102.2 (2.7)	102.1 (3.0)
Serum Creatinine (mmol /L); median (IQR)	71.0 (62.5, 80.0)	72.0 (66.0, 84.0)
eGFR (ml /min); median (IQR)[f]	90.0 (74.5, 90.0)	85.5 (73, 90.0)
Glucose (mmol /L); median (IQR)[g]	5.5 (4.8, 6.3)	5.4 (4.8, 6.3)
Bilirubin (µmol /L); median (IQR)[h]	9.0 (6.0, 12.0)	10.0 (6.0, 16.0)
Albumin (g /L); mean (SD)[i]	42.3 (3.8)	42.7 (3.8)
ALT (units / L); median (IQR)[j]	20.0 (16.0, 27.5)	26.5 (19.0, 40.5)
Total protein (g/ L); mean (SD)[k]	69.0 (4.5)	69.8 (3.7)
History of varicella zoster (yes)[l]	55 (91.7%)	47 (87.0%)
**VISUAL FUNCTION QUALITY OF LIFE QUESTIONNAIRE[m]**		
Summary score; median (IQR)[n]	1.3 (0.7, 2.1)	0.9 (0.5, 2.2)
Embarrassment score; median (IQR)	0.0 (0.0, 2.0)	0.0 (0.0, 1.0)
Anger score; median (IQR)	2.0 (1.0, 4.0)	2.0 (1.0, 3.0)
Depression score; median (IQR)	0.0 (0.0, 2.0)	0.0 (0.0, 2.0)
Loneliness score; median (IQR)	2.0 (0.0, 3.0)	1.0 (0.0, 2.0)
Fear of deterioration in vision score; median (IQR)	3.0 (2.0, 4.0)	3.0 (2.0, 4.0)
Safety at home score; median (IQR)	0.0 (0.0, 1.0)	0.0 (0.0, 1.0)

	Randomised to adalimumab (n=60)	Randomised to placebo (n=55)
Safety outside the home score; median (IQR)[n]	1.0 (0.0, 2.0)	0.5 (0.0, 2.0)
Coping with daily life score; median (IQR)[n]	1.0 (0.0, 2.0)	1.0 (0.0, 2.0)
Inability to do preferred activities score; median (IQR)	1.0 (0.0, 2.0)	1.0 (0.0, 2.0)
Life-interference	1.0 (0.0, 2.0)	1.0 (0.0, 2.0)
**WORK PRODUCTIVITY AND ACTIVITY IMPAIRMENT QUESTIONNAIRE**		
Employment status (employed)	45 (75.0%)	36 (65.5%)
Percentage of work time missed because of problem associated with uveitis (Absenteeism); median (IQR) [o]	0.0 (0.0, 0.0)	0.0 (0.0, 0.0)
Percentage of impairment while working because of problem associated with uveitis (Presenteeism); median (IQR)[op]	10.0 (0.0, 40.0)	20.0 (0.0, 30.0)
Percentage of overall work impairment [op]; median (IQR)	14.2 (0.0, 40.0)	20.0 (0.0, 30.0)
Percentage of daily activity impairment [o] ; median (IQR)	10.0 (0.0, 30.0)	10.0 (0.0, 25.0)
**OTHER QUALITY OF LIFE QUESTIONNAIRES**		
EQ-5D-5L utility score; median (IQR)	0.88 (0.74, 0.99)	0.87 (0.79, 0.99)
EQ-5D-5L visual analogue scale score; median (IQR)	80.0 (65.0, 87.5)	80.0 (75.0, 90.0)
Symptoms of side-effects summary score; median (IQR)[q]	2.3 (1.0, 3.3)	1.6 (1.0, 3.0)
**UVEITIS HISTORY**		
Primary uveitis diagnosis		
Undifferentiated anterior uveitis (Anterior uveitides)	8 (13.3%)	0 (0.0%)
HLA-B27 uveitis without spondylitis (Anterior uveitides)	0 (0.0%)	1 (1.8%)
Anterior uveitis with spondylitis (Anterior uveitides)	0 (0.0%)	0 (0.0%)
JIA-associated uveitis (Anterior uveitides)	0 (0.0%)	0 (0.0%)
Anterior uveitis with systemic disease other than spondylitis (Anterior uveitides)	1 (1.7%)	0 (0.0%)
Undifferentiated anterior/intermediate uveitis (Intermediate uveitides)	5 (8.3%)	5 (9.1%)
Pars planitis (Intermediate uveitides)	3 (5.0%)	1 (1.8%)

	Randomised to adalimumab (n=60)	Randomised to placebo (n=55)
Intermediate uveitis with systemic disease (Intermediate uveitides)	3 (5.0%)	2 (3.6%)
Undifferentiated posterior uveitis (Posterior uveitides)	3 (5.0%)	3 (5.5%)
Retinal vasculitis (Posterior uveitides)	4 (6.7%)	3 (5.5%)
Serpiginous choroiditis (Posterior uveitides)	0 (0.0%)	1 (1.8%)
Multifocal choroiditis without panuveitis (Posterior uveitides)	0 (0.0%)	1 (1.8%)
Multifocal choroiditis with panuveitis (Posterior uveitides)	1 (1.7%)	2 (3.6%)
Punctate inner choroiditis (Posterior uveitides)	4 (6.7%)	0 (0.0%)
Birdshot retinochoroiditis (Posterior uveitides)	18 (30.0%)	17 (30.9%)
Posterior uveitis with systemic disease (Posterior uveitides)	3 (5.0%)	3 (5.5%)
Undifferentiated panuveitis (Panuveitis)	5 (8.3%)	10 (18.2%)
Vogt-Koyanagi Harada (VKH) disease (Panuveitis)	0 (0.0%)	2 (3.6%)
Sympathetic Ophthalmia (Panuveitis)	0 (0.0%)	0 (0.0%)
Panuveitis with systemic disease other than VKH or Behçet (Panuveitis)	0 (0.0%)	3 (5.5%)
Any other uveitis diagnosis (Other)	2 (3.3%)	1 (1.8%)
**BCVA AND OCULAR EXAM**		
IOP measurement; median (IQR)[r]		
Left eye	16.0 (13.0, 19.0)	15.0 (12.0, 18.0)
Right eye	16.0 (13.0, 19.0)	15.0 (12.0, 17.0)
Cataract present in both eyes	8 (13.3%)	11 (20.0%)
If present, centrally involving in both eyes	7 (87.5%)	3 (27.3%)
If present, centrally involving in one eye	0 (0.0%)	1 (9.1%)
None	1 (12.5%)	7 (63.6%)
Cataract present in one eye	4 (6.7%)	7 (12.7%)
If present, centrally involving	3 (75.0%)	5 (71.4%)
No Cataract present	48 (80.0%)	37 (67.3%)
Lens status		
Both Phakic	50 (83.3%)	45 (81.8%)

	Randomised to adalimumab (n=60)	Randomised to placebo (n=55)
Both Pseudophakic	8 (13.3%)	4 (7.3%)
Both Aphakic	0 (0.0%)	1 (1.8%)
Phakic & Pseudophakic	2 (3.3%)	5 (9.1%)
Any non-ocular medical abnormality identified during ocular exam[s]	1 (1.7%)	3 (5.5%)
Cotton wool spots / intraretinal haemorrhage / other retinal lesion		
Both eyes	4 (6.7%)	0 (0.0%)
One eye	1 (1.7%)	2 (3.6%)
None	55 (91.7%)	53 (96.4%)
Other active disease in the retina and optic nerve		
Both eyes	2 (3.3%)	3 (5.5%)
One eye	1 (1.7%)	2 (3.6%)
None	57 (95.0%)	50 (90.9%)
Pars plana snowbanking		
Both eyes	3 (5.0%)	3 (5.5%)
One eye	1 (1.7%)	0 (0.0%)
None	56 (93.3%)	52 (94.5%)
Hyper– or hypo– chorioretinal pigmented (inactive) scars		
Both eyes	19 (31.7%)	12 (21.8%)
One eye	7 (11.7%)	4 (7.3%)
None	34 (56.7%)	39 (70.9%)
Choroidal neovascularisation		
Both eyes	0 (0.0%)	2 (3.6%)
One eye	2 (3.3%)	2 (3.6%)
None	58 (96.7%)	51 (92.7%)
Disc swelling		
Both eyes	4 (6.7%)	6 (10.9%)
One eye	3 (5.0%)	4 (7.3%)
None	53 (88.3%)	45 (81.8%)
Retinal vascular occlusion		
Both eyes	1 (1.7%)	0 (0.0%)
One eye	0 (0.0%)	1 (1.8%)
None	59 (98.3%)	54 (98.2%)
Pre-retinal or optic disc neovascularisation		
Both eyes	0 (0.0%)	0 (0.0%)

	Randomised to adalimumab (n=60)		Randomised to placebo (n=55)	
One eye	0 (0.0%)		2 (3.6%)	
None	60 (100.0%)		53 (96.4%)	
Other fundus abnormalities related to uveitis				
Both eyes	1 (1.7%)		3 (5.5%)	
One eye	2 (3.3%)		1 (1.8%)	
None	57 (95.0%)		51 (92.7%)	
Rhegmatogenous (old or new) retinal detachment				
Both eyes	0 (0.0%)		0 (0.0%)	
One eye	0 (0.0%)		1 (1.8%)	
None	60 (100.0%)		54 (98.2%)	
Epiretinal membrane (with or without lamellar hole)				
Both eyes	13 (21.7%)		9 (16.4%)	
One eye	12 (20.0%)		10 (18.1%)	
None	35 (58.3%)		36 (65.5%)	
Glaucomatous cupping				
Both eyes	1 (1.7%)		1 (1.8%)	
One eye	0 (0.0%)		0 (0.0%)	
None	59 (98.3%)		54 (98.2%)	
Optic atrophy				
Both eyes	0 (0.0%)		0 (0.0%)	
One eye	1 (1.7%)		1 (1.8%)	
None	59 (98.3%)		54 (98.2%)	
Other structural fundus abnormalities				
Both eyes	4 (6.7%)		0 (0.0%)	
One eye	0 (0.0%)		0 (0.0%)	
None	56 (93.3%)		55 (100.0%)	
	Left eye	Right eye	Left eye	Right eye
AC inflammatory cells in the anterior chamber visible in a 1mm height narrow slit beam				
0	47 (78.3%)	45 (75.0%)	38 (69.1%)	40 (72.8%)
+0.5	10 (16.7%)	11 (18.3%)	9 (16.4%)	7 (12.7%)
+1.0	2 (3.3%)	2 (3.3%)	8 (14.6%)	8 (14.6%)
+2.0	1 (1.7%)	2 (3.3%)	0 (0.0%)	0 (0.0%)

	Randomised to adalimumab (n=60)		Randomised to placebo (n=55)	
Vitreous cells seen in 1 X 0.5mm beam				
0	27 (45.0%)	26 (43.3%)	18 (32.7%)	23 (41.8%)
+0.5	18 (30.0%)	17 (28.3%)	22 (40.0%)	19 (34.6%)
+1.0	11 (18.3%)	12 (20.0%)	13 (23.6%)	11 (20.0%)
+2.0	4 (6.7%)	4 (6.7%)	2 (3.6%)	2 (3.6%)
+3.0	0 (0.0%)	1 (1.7%)	0 (0.0%)	0 (0.0%)
Vitreous haze				
0	40 (66.7%)	38 (63.3%)	40 (72.7%)	40 (72.7%)
+0.5	15 (25.0%)	15 (25.0%)	14 (25.5%)	12 (21.8%)
+1.0	1 (1.7%)	5 (8.3%)	1 (1.8%)	2 (3.6%)
+2.0	4 (6.7%)	1 (1.7%)	0 (0.0%)	0 (0.0%)
Cannot access	0 (0.0%)	1 (1.7%)	0 (0.0%)	1 (1.8%)
**OCT EVALUATION**				
Subretinal fluid present[t]	3 (5.0%)	2 (3.3%)	3 (5.6%)	2 (3.7%)
Cystoid spaces present	9 (15.0%)	9 (15.0%)	6 (11.1%)	9 (16.7%)
OCT machine used to capture OCT image				
Heidelberg	47 (78.3%)		38 (69.1%)	
Topcon	13 (21.7%)		17 (30.9%)	
Zeiss	0 (0.0%)		0 (0.0%)	
Other	0 (0.0%)		0 (0.0%)	
**OCULAR MEDICAL HISTORY**				
Ocular hypertension (IOP >21mmHg or requiring medication				
Both eyes	7 (11.7%)		7 (12.7%)	
One eye	7 (11.7%)		4 (7.3%)	
None	46 (76.7%)		44 (80.0%)	
Glaucoma (ocular hypertension with glaucomatous optic nerve damage or visual field loss				
Both eyes	1 (1.7%)		3 (5.5%)	
One eye	3 (5.0%)		1 (1.8%)	
None	56 (93.3%)		51 (92.7%)	

	Randomised to adalimumab (n=60)	Randomised to placebo (n=55)
Macular oedema		
Both eyes	20 (33.3%)	16 (29.1%)
One eye	8 (13.3%)	8 (14.5%)
None	32 (53.3%)	31 (56.4%)
Epiretinal membrane		
Both eyes	10 (16.7%)	8 (14.5%)
One eye	13 (21.7%)	7 (12.7%)
None	37 (61.7%)	40 (72.7%)
Optic nerve oedema		
Both eyes	6 (10.0%)	10 (18.2%)
One eye	2 (3.3%)	4 (7.3%)
None	52 (86.7%)	41 (74.5%)
Pre-retinal/optic nerve neovascularisation		
Both eyes	0 (0.0%)	0 (0.0%)
One eye	0 (0.0%)	1 (1.8%)
None	60 (100.0%)	54 (98.2%)
Traction/rhegmatogenous retinal detachment		
Both eyes	0 (0.0%)	0 (0.0%)
One eye	0 (0.0%)	1 (1.8%)
None	60 (100.0%)	54 (98.2%)
Vitreous haemorrhage		
Both eyes	0 (0.0%)	0 (0.0%)
One eye	1 (1.7%)	0 (0.0%)
None	59 (98.3%)	55 (100.0%)
Amblyopia		
Both eyes	0 (0.0%)	0 (0.0%)
One eye	0 (0.0%)	2 (3.6%)
None	60 (100.0%)	53 (96.4%)
Other ocular medical history		
Both eyes	4 (6.7%)	2 (3.6%)
One eye	3 (5.0%)	2 (3.6%)
None	53 (88.3%)	51 (92.7%)
**OCULAR PROCEDURE HISTORY**		
Cataract surgery		
Both eyes	7 (11.7%)	3 (5.5%)
One eye	2 (3.3%)	6 (10.9%)
None	51 (85.0%)	46 (83.6%)

	Randomised to adalimumab (n=60)	Randomised to placebo (n=55)
Vitrectomy		
Both eyes	0 (0.0%)	0 (0.0%)
One eye	2 (3.3%)	2 (3.6%)
None	58 (96.7%)	53 (96.4%)
Panretinal photocoagulation /laser cryotherapy		
Both eyes	3 (5.0%)	0 (0.0%)
One eye	1 (1.7%)	0 (0.0%)
None	56 (93.3%)	55 (100.0%)
Intravitreal corticosteroid injections		
Both eyes	2 (3.3%)	3 (5.5%)
One eye	1 (1.7%)	2 (3.6%)
None	57 (95.0%)	50 (90.9%)
Periocular corticosteroids		
Both eyes	1 (1.7%)	2 (3.6%)
One eye	3 (5.0%)	2 (3.6%)
None	56 (93.3%)	51 (92.7%)
Dexamethasone implant (Ozurdex)/fluoscinolone implant (Iluvien)		
Both eyes	6 (10.0%)	6 (10.9%)
One eye	3 (5.0%)	3 (5.5%)
None	51 (85.0%)	46 (83.6%)
Glaucoma surgery e.g. filtration/shunt/stent		
Both eyes[u]	2 (3.3%)	1 (1.8%)
One eye	0 (0.0%)	0 (0.0%)
None	58 (96.7%)	54 (98.2%)
**NON-OCULAR MEDICAL HISTORY**		
Spondyloarthropathy	1 (1.7%)	0 (0.0%)
Psoriasis	2 (3.3%)	3 (5.5%)
Juvenile idiopathic arthritis	0 (0.0%)	0 (0.0%)
Sarcoidosis	4 (6.7%)	9 (16.4%)
Inflammatory bowel disease	2 (3.3%)	1 (1.8%)
Vogt-Koyanagi Harada disease	0 (0.0%)	2 (3.6%)
Other[v]	3 (5.0%)	7 (12.7%)
Diagnosed with any malignancy	0 (0.0%)	4 (7.3%)
Being actively monitored for any malignancy	0 (0.0%)	0 (0.0%)
Cardiovascular disease	4 (6.7%)	3 (5.5%)
**SMOKING STATUS**		

	Randomised to adalimumab (n=60)	Randomised to placebo (n=55)
Never smoked	23 (38.3%)	20 (36.4%)
Current cigarette smoker	3 (5.0%)	4 (7.3%)
Former cigarette smoker	9 (15.0%)	5 (9.1%)
Current alternative smoker	3 (5.0%)	3 (5.5%)
Former alternative smoker	20 (33.3%)	21 (38.2%)
**BASELINE MEDICATIONS**		
Pressure reducing eye drops	7 (12%)	7 (13%)
Steroid eye drops	13 (22%)	10 (18%)
Nonsteroidal anti-inflammatory drop	2 (3%)	1 (2%)

1 patient with missing data (1,0)

[b] 12 patients with missing data (5,7)

[c] 9 patients with missing data (4,5)

[d] 4 patients with missing data (1,3)

[e] 36 patients with missing data (18,18)

[f] 7 patients with missing data (4,3)

[g] 9 patients with missing data (5,4)

[h] 1 patient with missing data (0,1)

[i] 1 patient with missing data (0,1)

[j] 3 patients with missing data (0,3)

[k] 24 patients with missing data (12,12)

[l] 1 patient with missing data (0,1)

[m] Each item (and the summary score) ranges from 0 (“not at all”) to 5 (“all the time”), reflecting how frequently eyesight interferes with daily life

[n] 1 patient with missing data (0,1)

[o] Scores only computed for employed participants

[p] 8 patients with missing data (3,5)

[q] Score ranges from 1 (“Not at all bothersome”) to 5 (“extremely bothersome”) for medication side effects. 2 patients with missing data (1,1)

[r] 1 patient with missing data

[s] Reported events include: “inferior left premolar tooth ache” (adalimumab), “pigment deposits of anterior lens surface right eye & osteoporosis” (placebo), “Cutaneous lupus rash” (placebo) and “Hypertrophic Cardiomyopathy” (placebo)

[t] 1 patient with missing data (0,0,1,1)

[u] 2 patients underwent tube surgeries (1,1) with Mitomycin C as the anti-proliferative agent. 1 patient had a Preserflo procedure (1,0)

[v] Other non-ocular medical history in the adalimumab group included asthma, irritable bowel syndrome and pre-diabetic, and allergic rhinitis. In the placebo group, reported conditions were Bell’s palsy, Sjögren’s syndrome, tattoo inflammation, hypertension, tuberculosis, cutaneous lupus, and Suspect current uveitis is VKH although no CNS or hearing issues.

Table ST7 Baseline characteristics of participants completing the TRI

	TRI responder (n=119)	TRI non-responder (n=63)
**DEMOGRAPHIC CHARACTERISTICS**		
Sex (male)	43.0 (36.1%)	29.0 (46.0%)
Age (years); median (IQR)	50.0 (37.0, 58.0)	55.0 (39.0, 66.0)
Ethnicity (White or Caucasian)	106.0 (89.1%)	54.0 (85.7%)
Weight (kg); median (IQR)	82.0, (69.0, 95.0)	83.0 (70.0, 100.0)
**CLINICAL CHARACTERISTICS AND BLOOD TESTS**		
Heart rate (bpm); mean (SD)[a]	76.1 (11.7)	72.2 (12.3)
Diastolic blood pressure (mm Hg); mean (SD)	81.1 (10.0)	81.0 (10.7)
Systolic blood pressure (mm Hg); mean (SD)	136.0 (20.1)	137.7 (20.8)
Blood samples taken (yes)	119.0 (100.0%)	63.0 (100.0%)
WBC ($x10^9$ /L); median (IQR) [b]	8.5 (6.5, 10.7)	9.1 (6.6, 11.5)
Lymphocytes ($x10^9$ /L); median (IQR) [c]	1.3 (0.9, 2.0)	1.7 (1.1, 2.3)
Neutrophils ($x10^9$ /L); mean (SD)[d]	6.7 (2.9)	6.7 (2.9)
Haematocrit (%); mean (SD)[e]	42.7 (3.7)	42.0 (3.7)
Platelets ($x10^9$ /L); mean (SD)[f]	284.9 (67.0)	260.1 (60.3)
Hb (g /L); mean (SD)[g]	142.4 (13.3)	142.1 (12.8)
MCV (fL); mean (SD)[h]	90.7 (5.4)	90.1 (5.6)
Urea (mmol /L); median (IQR)[i]	5.0 (4.1, 5.9)	5.2 (4.1, 6.7)
Potassium (mmol /L); mean (SD)[j]	4.2 (0.4)	4.3 (0.4)
Sodium (mmol /L); mean (SD)[k]	140.0 (1.8)	139.8 (2.1)
Chloride (mmol /L); mean (SD)[l]	102.3 (2.9)	103.1 (2.9)
Serum Creatinine (mmol /L); median (IQR)[m]	71.0 (65.0, 81.0)	72.0 (64.0, 82.0)
eGFR (ml /min); median (IQR)[n]	88.0 (73.5, 90.0)	90.0 (77.0, 90.0)
Glucose (mmol /L); median (IQR)[o]	5.5 (4.8, 6.3)	5.4 (4.8, 6.5)
Bilirubin (µmol /L); median (IQR)[p]	9.0 (6.0, 12.0)	9.0 (6.0, 11.0)
Albumin (g /L); mean (SD)[q]	42.5 (3.7)	42.2 (3.9)
ALT (units / L); median (IQR)[r]	22.0 (17.0, 32.5)	23.0 (18.0, 30.0)
Total protein (g/ L); mean (SD)[s]	69.4 (4.1)	69.7 (4.1)
History of varicella zoster (yes)[t]	104.0 (87.4%)	43.0 (68.3%)
**VISUAL FUNCTION QUALITY OF LIFE QUESTIONNAIRE[n]**		
Summary score; median (IQR)[u]	1.3 (0.6, 2.1)	1.6 (0.7, 2.3)
Embarrassment score; median (IQR)	0.0 (0.0, 2.0)	1.0 (0.0, 2.0)
Anger score; median (IQR)	2.0 (1.0, 3.0)	3.0 (2.0, 4.0)
Loneliness score; median (IQR)	0.0 (0.0, 2.0)	0.0 (0.0, 1.0)

	TRI responder (n=119)	TRI non-responder (n=63)
Depression score; median (IQR)	2.0 (0.0, 3.0)	2.0 (0.0, 3.0)
Fear of deterioration in vision score; median (IQR)[v]	3.0 (2.0, 4.0)	3.0 (2.0, 4.0)
Safety at home score; median (IQR)[w]	0.0 (0.0, 1.0)	1.0 (0.0, 2.0)
Safety outside the home score; median (IQR)[x]	1.0 (0.0, 2.0)	2.0 (0.0, 2.0)
Coping with daily life score; median (IQR)[y]	1.0 (0.0, 2.0)	2.0 (0.0, 2.0)
Inability to do preferred activities score; median (IQR)[z]	1.0 (0.0, 2.0)	1.0 (0.0, 2.0)
Life-interference score; median (IQR)[aa]	1.0 (0.0, 2.0)	2.0 (1.0, 3.0)
**WORK PRODUCTIVITY AND ACTIVITY IMPAIRMENT QUESTIONNAIRE**		
Employment status (employed); n (%)	83.0 (69.8%)	33.0 (52.4%)
Percentage of work time missed because of problem associated with uveitis (Absenteeism); median (IQR) [ab]	0.0 (0.0, 0.0)	0.0 (0.0, 0.0)
Percentage of impairment while working because of problem associated with uveitis (Presenteeism); median (IQR)[ab ac]	20.0 (0.0, 30.0)	20.0 (10.0, 50.0)
Percentage of overall work impairment; median (IQR) [ab ac]	20.0 (0.0, 30.0)	20.0 (10.0, 50.0)
Percentage of daily activity impairment; median (IQR)[ab]	10.0 (0.0, 30.0)	20.0 (0.0, 30.0)
**OTHER QUALITY OF LIFE QUESTIONNAIRES**		
EQ-5D-5L utility score; median (IQR)[ad]	0.87 (0.75, 0.99)	0.89 (0.77, 0.99)
EQ-5D-5L visual analogue scale score; median (IQR)	80.0 (70.0, 90.0)	80.0 (75.0, 90.0)
Symptoms of side-effects summary score; median (IQR)[ae]	1.0 (0.0, 2.3)	1.0 (0.0, 2.3)
**UVEITIS HISTORY**		
Uveitis status		
Bilateral active	76 (63.9%)	39 (61.9%)
Unilateral active (one eye active and one inactive)	12 (10.1%)	10 (15.9%)
Bilateral inactive	26 (21.9%)	10 (15.9%)
Unilateral uveitis (active)	3 (2.5%)	4 (6.4%)
Unilateral uveitis (inactive)	2 (1.7%)	0 (0.0%)
Primary uveitis diagnosis		
Undifferentiated anterior uveitis (Anterior uveitides)	8 (6.7%)	1 (1.6%)

	TRI responder (n=119)	TRI non-responder (n=63)
HLA-B27 uveitis without spondylitis (Anterior uveitides)	1 (0.8%)	1 (1.6%)
Anterior uveitis with spondylitis (Anterior uveitides)	0 (0.0%)	0 (0.0%)
JIA-associated uveitis (Anterior uveitides)	0 (0.0%)	0 (0.0%)
Anterior uveitis with systemic disease other than spondylitis (Anterior uveitides)	1 (0.8%)	0 (0.0%)
Undifferentiated anterior/intermediate uveitis (Intermediate uveitides)	10 (8.4%)	12 (19.1%)
Pars planitis (Intermediate uveitides)	4 (3.4%)	2 (3.2%)
Intermediate uveitis with systemic disease (Intermediate uveitides)	5 (4.2%)	4 (6.4%)
Undifferentiated posterior uveitis (Posterior uveitides)	6 (5.0%)	3 (4.8%)
Retinal vasculitis (Posterior uveitides)	7 (5.9%)	9 (14.3%)
Serpiginous choroiditis (Posterior uveitides)	1 (0.8%)	0 (0.0%)
Multifocal choroiditis without panuveitis (Posterior uveitides)	1 (0.8%)	1 (1.6%)
Multifocal choroiditis with panuveitis (Posterior uveitides)	4 (3.4%)	3 (4.8%)
Punctate inner choroiditis (Posterior uveitides)	5 (4.2%)	1 (1.6%)
Birdshot retinochoroiditis (Posterior uveitides)	36 (30.3%)	13 (20.6%)
Posterior uveitis with systemic disease (Posterior uveitides)	6 (5.0%)	2 (3.2%)
Undifferentiated panuveitis (Panuveitis)	16 (13.5%)	6 (9.5%)
Vogt-Koyanagi Harada (VKH) disease (Panuveitis)	2 (1.7%)	1 (1.6%)
Sympathetic Ophthalmia (Panuveitis)	0 (0.0%)	1 (1.6%)
Panuveitis with systemic disease other than VKH or Behçet (Panuveitis)	3 (2.5%)	2 (3.2%)
Any other uveitis diagnosis (Other)[af]	3 (2.5%)	1 (1.6%)
**BCVA AND OCULAR EXAM**		
BCVA score; median (IQR)		
Left eye[ag]	85.0 (77.0, 89.0)	79.0 (68.0, 85.0)
Right eye[ah]	84.0 (77.0, 87.0)	80.0 (70.0, 85.0)

	**TRI responder (n=119)**	**TRI non-responder (n=63)**
IOP measurement; median (IQR)[ai]		
Left eye	16.0 (13.0, 18.0)	16.0 (13, 18.0)
Right eye	15.0 (12.0, 19.0)	15.0 (13.0, 17.0)
Cataract present in both eyes	19 (16.0%)	12 (19.0%)
If present, centrally involving in both eye	10 (52.6%)	9 (75.0%)
If present, centrally involving in either one eye	1 (5.3%)	1 (8.3%)
None	8 (42.1%)	2 (16.7%)
Cataract present in one eye	12 (10.1%)	8 (12.7%)
If present, centrally involving	9 (75.0%)	5 (62.5%)
No Cataract present	88 (73.9%)	43 (68.3%)
Lens status		
Both Phakic	98 (82.4%)	38 (60.3%)
Both Pseudophakic	12 (10.1%)	16 (25.4%)
Both Aphakic	1 (0.8%)	0 (0.0%)
Phakic & Pseudophakic	8 (6.7%)	8 (12.7%)
Phakic & Aphakic	0 (0.0%)	1 (1.6%)
Any non-ocular medical abnormality identified during ocular exam[aj]	4 (3.4%)	1 (1.6%)
Retinal vasculitis / sheathing		
Both eyes	41 (34.5%)	19 (30.2%)
One eye	6 (5.0%)	5 (7.9%)
None	72 (60.5%)	39 (61.9%)
Cotton wool spots / intraretinal haemorrhage / other retinal lesion		
Both eyes	4 (3.4%)	3 (4.8%)
One eye	3 (2.5%)	0 (0.0%)
None	112 (94.1%)	60 (95.2%)
Macular oedema present		
Both eyes	14 (11.8%)	18 (28.6%)
One eye	18 (15.1%)	14 (22.2%)
None	87 (73.1%)	31 (49.2%)
Active choroidal / chorioretinal lesions		
Both eyes	31 (26.1%)	14 (22.2%)
One eye	6 (5.0%)	5 (7.9%)
None	82 (68.9%)	44 (69.8%)
Other active disease in the retina and optic nerve		
Both eyes	5 (4.2%)	1 (1.6%)

	TRI responder (n=119)	TRI non-responder (n=63)
One eye	3 (2.5%)	0 (0.0%)
None	111 (93.3%)	62 (98.4%)
Pars plana snowbanking		
Both eyes	6 (5.0%)	4 (6.3%)
One eye	1 (0.8%)	0 (0.0%)
None	112 (94.1%)	59 (93.7%)
Hyper– or hypo– chorioretinal pigmented (inactive) scars		
Both eyes	34 (28.6%)	22 (34.9%)
One eye	12 (10.1%)	3 (4.8%)
None	73 (61.3%)	38 (60.3%)
Choroidal neovascularisation		
Both eyes	3 (2.5%)	1 (1.6%)
One eye	5 (4.2%)	0 (0.0%)
None	111 (93.3%)	62 (98.4%)
Disc swelling		
Both eyes	10 (8.4%)	4 (6.3%)
One eye	7 (5.9%)	6 (9.5%)
None	102 (85.7%)	53 (84.1%)
Retinal vascular occlusion		
Both eyes	1 (0.8%)	0 (0.0%)
One eye	1 (0.8%)	2 (3.2%)
None	117 (98.3%)	61 (96.8%)
Pre-retinal or optic disc neovascularisation		
Both eyes	0 (0.0%)	0 (0.0%)
One eye	2 (1.7%)	1 (1.6%)
None	117 (98.3%)	62 (98.4%)
Other fundus abnormalities related to uveitis		
Both eyes	4 (3.4%)	3 (4.8%)
One eye	3 (2.5%)	3 (4.8%)
None	112 (94.1%)	57 (90.5%)
Rhegmatogenous (old or new) retinal detachment		
Both eyes	0 (0.0%)	0 (0.0%)
One eye	1 (0.8%)	2 (3.2%)
None	118 (99.2%)	61 (96.8%)
Epiretinal membrane (with or without lamellar hole)		
Both eyes	23 (19.3%)	21 (33.3%)
One eye	22 (18.5%)	12 (19.0%)

	TRI responder (n=119)		TRI non-responder (n=63)	
None	74 (62.2%)		30 (47.6%)	
Glaucomatous cupping				
Both eyes	2 (1.7%)		3 (4.8%)	
One eye	1 (0.8%)		2 (3.2%)	
None	116 (97.5%)		58 (92.1%)	
Optic atrophy				
Both eyes	0 (0.0%)		0 (0.0%)	
One eye	2 (1.7%)		1 (1.6%)	
None	117 (98.3%)		62 (98.4%)	
Other structural fundus abnormalities				
Both eyes	4 (3.4%)		1 (1.6%)	
One eye	0 (0.0%)		3 (4.8%)	
None	115 (96.6%)		59 (93.7%)	
	Left eye	Right eye	Left eye	Right eye
AC inflammatory cells in the anterior chamber visible in a 1mm height narrow slit beam				
0	89 (74.8%)	89 (74.8%)	52 (82.5%)	56 (88.9%)
+0.5	19 (16.0%)	18 (15.1%)	8 (12.7%)	4 (6.4%)
+1.0	10 (8.4%)	10 (8.4%)	2 (3.2%)	3 (4.8%)
+2.0	1 (0.8%)	2 (1.7%)	1 (1.6%)	0 (0.0%)
Vitreous cells seen in 1 X 0.5mm beam				
0	49 (41.2%)	53 (44.5%)	23 (36.5%)	23 (36.5%)
+0.5	40 (33.6%)	36 (30.3%)	18 (28.6%)	17 (27.0%)
+1.0	24 (20.2%)	23 (19.3%)	15 (23.8%)	18 (28.6%)
+2.0	6 (5.0%)	6 (5.0%)	5 (7.9%)	4 (6.4%)
+3.0	0 (0.0%)	1 (0.8%)	1 (1.6%)	1 (1.6%)
Cannot access	0 (0.0%)	0 (0.0%)	1 (1.6%)	0 (0.0%)
Vitreous haze				
0	84 (70.1%)	82 (68.9%)	30 (47.6%)	31 (49.2%)

	TRI responder (n=119)		TRI non-responder (n=63)	
+0.5	29 (24.4%)	27 (22.7%)	17 (27.0%)	17 (27.0%)
+1.0	2 (1.7%)	7 (5.9%)	10 (15.9%)	12 (19.1%)
+2.0	4 (3.4%)	1 (0.8%)	4 (6.4%)	3 (4.8%)
+4.0	0 (0.0%)	0 (0.0%)	1 (1.6%)	0 (0.0%)
Cannot access	0 (0.0%)	2 (1.7%)	1 (1.6%)	0 (0.0%)
**OCT EVALUATION**				
Central subfield thickness; median (IQR) [ak]	286.0 (254.0, 313.0)	279.5 (254.0, 309.0)	318.0 (278.0, 357.0)	319.0 (279.0, 354.0)
Subretinal fluid present[al]	6 (5.1%)	4 (3.4%)	6 (9.5%)	3 (4.8%)
Cystoid spaces present[am]	16 (13.6%)	19 (16.1)	22 (34.9%)	26 (41.3%)
OCT machine used to capture OCT image				
Heidelberg	88 (74.0%)		56 (88.9%)	
Topcon	31 (26.1%)		5 (7.9%)	
Zeiss	0 (0.0%)		2 (3.2%)	
Other	0 (0.0%)		0 (0.0%)	
**OCULAR MEDICAL HISTORY**				
Ocular hypertension (IOP >21mmHg or requiring medication				
Both eyes	14 (11.8%)		15 (23.8%)	
One eye	12 (10.1%)		5 (7.9%)	
None	93 (78.2%)		43 (68.3%)	
Glaucoma (ocular hypertension with glaucomatous optic nerve damage or visual field loss				
Both eyes	4 (3.4%)		3 (4.8%)	
One eye	4 (3.4%)		1 (1.6%)	
None	111 (93.3%)		59 (93.7%)	
Macular oedema				
Both eyes	36 (30.3%)		22 (34.9%)	
One eye	18 (15.1%)		14 (22.2%)	
None	65 (54.6%)		27 (42.9%)	
Epiretinal membrane				
Both eyes	19 (16.0%)		23 (36.5%)	
One eye	20 (16.8%)		8 (12.7%)	
None	80 (67.2%)		32 (50.8%)	

	TRI responder (n=119)	TRI non-responder (n=63)
Optic nerve oedema		
Both eyes	16 (13.4%)	5 (7.9%)
One eye	6 (5.0%)	3 (4.8%)
None	97 (81.5%)	55 (87.3%)
Pre-retinal/optic nerve neovascularisation		
Both eyes	0 (0.0%)	0 (0.0%)
One eye	1 (0.8%)	0 (0.0%)
None	118 (99.2%)	63 (100.0%)
Traction/rhegmatogenous retinal detachment		
Both eyes	0 (0.0%)	0 (0.0%)
One eye	1 (0.8%)	0 (0.0%)
None	118 (99.2%)	63 (100.0%)
Vitreous haemorrhage		
Both eyes	0 (0.0%)	0 (0.0%)
One eye	1 (0.8%)	1 (1.6%)
None	118 (99.2%)	62 (98.4%)
Amblyopia		
Both eyes	0 (0.0%)	0 (0.0%)
One eye	2 (1.7%)	1 (1.6%)
None	117 (98.3%)	62 (98.4%)
Other ocular medical history		
Both eyes	6 (5.0%)	4 (6.3%)
One eye	6 (5.0%)	4 (6.3%)
None	107 (89.9%)	55 (87.3%)
**OCULAR PROCEDURE HISTORY**		
Cataract surgery		
Both eyes	10 (8.4%)	12 (19.0%)
One eye	9 (7.6%)	11 (17.5%)
None	100 (84.0%)	40 (63.5%)
Vitrectomy		
Both eyes	0 (0.0%)	2 (3.2%)
One eye	4 (3.4%)	6 (9.5%)
None	115 (96.6%)	55 (87.3%)
Panretinal photocoagulation /laser cryotherapy		
Both eyes	3 (2.5%)	1 (1.6%)
One eye	1 (0.8%)	4 (6.3%)
None	115 (96.6%)	58 (92.1%)
Intravitreal corticosteroid injections		

	TRI responder (n=119)	TRI non-responder (n=63)
Both eyes	5 (4.2%)	5 (7.9%)
One eye	3 (2.5%)	4 (6.3%)
None	111 (93.3%)	54 (85.7%)
Periocular corticosteroids		
Both eyes	3 (2.5%)	1 (1.6%)
One eye	5 (4.2%)	9 (14.3%)
None	111 (93.3%)	53 (84.1%)
Dexamethasone implant (Ozurdex)/fluoscinolone implant (Iluvien)		
Both eyes	12 (10.1%)	7 (11.1%)
One eye	6 (5.0%)	6 (9.5%)
None	101 (84.9%)	50 (79.4%)
Glaucoma surgery e.g. filtration/shunt/stent		
Both eyes[an]	3 (2.5%)	0 (0.0%)
One eye[ao]	0 (0.0%)	1 (1.6%)
None	116 (97.5%)	62 (98.4%)
**NON-OCULAR MEDICAL HISTORY**		
Spondyloarthropathy	1 (0.8%)	0 (0.0%)
Psoriasis	5 (4.2%)	3 (4.8%)
Juvenile idiopathic arthritis	0 (0.0%)	0 (0.0%)
Sarcoidosis	13 (10.9%)	8 (12.7%)
Inflammatory bowel disease	3 (2.5%)	2 (3.2%)
Vogt-Koyanagi Harada disease	2 (1.7%)	1 (1.6%)
Other[ap]	10 (8.4%)	7 (11.1%)
Diagnosed with any malignancy	4 (3.4%)	3 (4.8%)
Being actively monitored for any malignancy	0 (0.0%)	0 (0.0%)
Cardiovascular disease	7 (5.9%)	5 (7.9%)
**SMOKING STATUS[aq]**		
Never smoked	45 (37.8%)	23 (36.5%)
Current cigarette smoker	7 (5.9%)	4 (6.3%)
Former cigarette smoker	15 (12.6%)	6 (9.5%)
Current alternative smoker	6 (5.0%)	1 (1.6%)
Former alternative smoker	42 (35.3%)	23 (36.5%)

[a] 2 patients with missing data (1,1)

[b] 1 patient with missing data (0,1)

[c] 1 patient with missing data (0,1)

[d] 1 patient with missing data (0,1)

[e] 21 patient with missing data (13,8)

[f] 1 patient with missing data (0,1)

[g] 1 patient with missing data (0,1)
[h] 1 patient with missing data (0,1)
[i] 14 patients with missing data (9,5)
[j] 6 patients with missing data (4,2)
[k] 1 patient with missing data (0,1)
[l] 68 patients with missing data (39,29)
[m] 2 patients with missing data (0,2)
[n] 13 patients with missing data (7,6)
[o] 15 patients with missing data (9,6)
[p] 2 patients with missing data (1,1)
[q] 2 patients with missing data (1,1)
[r] 5 patients with missing data (3,2)
[s] 39 patients with missing data (25,14)
[t] 1 patient with missing data (1,0)
[u] 2 patients with missing data (1,1)
[v] 1 patient with missing data (0,1)
[w] 1 patient with missing data (0,1)
[x] 2 patients with missing data (1,1)
[y] 2 patients with missing data (1,1)
[z] 1 patient with missing data (0,1)
[aa] 1 patient with missing data (0,1)
[ab] Scores only computed for employed participants
[ac]12 patients with missing data (8,4)
[ad] 1 patient with missing data (1,0)
[ae] 3 patients with missing data (2,1)
[af] Other uveitis diagnoses are acute posterior multifocal placoid pigment epitheliopathy APMPPE (responder), Azoor (responder), right optic nerve granuloma (responder) and retinal vasculitis (non-responder)
[ag] 2 patients had a BCVA of 0 (1,1), with vision at the level of light perception (LP) and no light perception (NLP) respectively
[ah] 2 patients had a BCVA of 0 (2,0), with vision at the level of counting fingers (CF) and hand movement (HM) respectively
[ai] 1 patient with missing data (1,0)
[aj] Reported events include: 'inferior left premolar tooth ache' (responder), 'pigment deposits of anterior lens surface right eye & osteoporosis' (responder), 'Cutaneous lupus rash' (responder), 'Hypertrophic Cardiomyopathy' (responder) and CT Chest show evidence of pulmonary sarcoidosis (non-responder)
[ak] 2 patients with missing data (1,1,0,0)
[al] 2 patients with missing data (1,1,0,0)
[am] 2 patients with missing data (1,1,0,0)
[an] 2 patients underwent tube surgeries with Mitomycin C as the anti-proliferative agent; 1 patient had a Preserflo procedure
[ao] 1 patient had GATT procedure
[ap] Other non-ocular medical history in the responder group included allergic rhinitis, irritable bowel syndrome and pre-diabetic, tattoo inflammation, Bell's palsy, cutaneous lupus, tuberculosis, hypertension, asthma, Sjögren's syndrome, and Suspect current uveitis is VKH although no CNS or hearing issues. In the non-responder group, reported conditions were Lichen sclerosis, polymyalgia rheumatica and hypertension eumatica, psoriatic

arthropathy, endometriosis and depression, osteochondroma of femoral head, Diverticulitis and GORD, and mild asthma

[aq] 1 patient with missing data (1,0).

Table ST8 Sensitivity analysis based on per protocol population

	Randomised to adalimumab (n=58)	Randomised to placebo (n=50)	Hazard Ratio (95% CI)	p-value
Proportion with treatment failure at 12 months	28.8%	59.2%	0.39 (0.21, 0.72)	0.002

Table ST9 Individual components of treatment failure outcome

	Randomised to adalimumab (n=60)	Randomised to placebo (n=55)
**Treatment failure in RCT**	19/60 (31.7%)	31/55 (56.4%)
**Treatment failure assessment criteria:[a]**		
≥15 letter decrease in BCVA, compared to the last TRI visit	4/19 (21.1%)	4/31 (12.9%)
New active inflammatory chorioretinal lesions	5/19 (26.3%)	8/31 (25.8%)
>20% increase in central subfield thickness, compared to the last TRI visit	3/19 (15.8%)	2/31 (6.5%)
Onset or worsening of retinal vasculitis	6/19 (31.6%)	8/31 (25.8%)
Two-step worsening of vitreous haze compared to score at the last TRI visit	2/19 (10.5%)	7/31 (22.6%)
Prescription of >5 mg/day oral prednisolone to maintain uveitis remission	11/19 (57.9%)	19/31 (61.3%)

[a] Not mutually exclusive, i.e. participant could meet more than one criteria.

Table ST10 Summary of longitudinal outcomes

		Randomised to adalimumab (n=60)	Randomised to placebo (n=55)	Treatment effect (95% CI)[b]	p-value
**EQ5D UTILITY SCORE[a]: Median (IQR), n**					
**TRI**	Baseline	0.88 (0.74, 0.99), 60	0.87 (0.79, 0.99), 55		
	Week 16	0.86 (0.67, 0.93), 60	0.87 (0.76, 0.99), 54		
**RCT**	Week 12	0.86 (0.63, 0.99), 55	0.84 (0.73, 0.98), 43		
	Week 24	0.86 (0.63, 0.99), 43	0.87 (0.71, 0.99), 32		
	Week 36	0.89 (0.65, 0.99), 37	0.80 (0.72, 0.99), 24		
	Week 48	0.89 (0.67, 0.99), 32	0.79 (0.63, 0.89), 14		
	Week 64	0.88 (0.56, 0.99), 26	0.89 (0.73, 0.98), 13		
	Week 80	0.79 (0.66, 0.99), 15	0.81 (0.65, 0.89), 9		
	Week 96	0.86 (0.67, 0.99), 9	0.79 (0.70, 0.80), 6		
	Week 112	0.86 (0.63, 0.99), 7	0.78 (0.69, 0.93), 6		
	Week 128	0.89 (0.68, 0.89), 4	0.74 (0.66, 0.89), 4		
Test for treatment*time interaction					0.39
Overall treatment effect estimate				MD[c]=0.027 (-0.020, 0.074)	0.26
**EQ5D VISUAL ANALOGUE SCORE[a]: Median (IQR), n**					
**TRI**	Baseline	80.0 (65.0, 87.5), 60	80.0 (75.0, 90.0), 55		
	Week 16	80.0 (70.0, 90.0), 60	80.0 (65.0, 90.0), 54		
**RCT**	Week 12	80.0 (65.0, 90.0), 55	75.0 (65.0, 90.0), 43		
	Week 24	80.0 (70.0, 90.0), 43	85.0 (60.0, 91.0), 32		
	Week 36	85.0 (65.0, 90.0), 37	82.5 (67.5, 91.0), 24		

		Randomised to adalimumab (n=60)	Randomised to placebo (n=55)	Treatment effect (95% CI)[b]	p-value
	Week 48	85.0 (72.5, 92.5), 32	70.0 (60.0, 90.0), 14		
	Week 64	82.5 (66.0, 90.0), 26	80.0 (70.0, 91.0), 13		
	Week 80	80.0 (70.0, 85.0), 15	86.0 (80.0, 95.0), 9		
	Week 96	65.0 (50.0, 85.0), 9	75.0 (50.0, 90.0), 6		
	Week 112	79.0 (60.0, 90.0), 7	57.5 (40.0, 85.0), 6		
	Week 128	85.0 (70.0, 90.0), 4	67.5 (45.0, 91.5), 4		
Test for treatment*time interaction					0.71
Overall treatment effect estimate				MD[c]=-1.10 (-4.92, 2.72)	0.57
**PATIENT REPORTED VISUAL FUNCTION[d]: Median (IQR), n**					
**TRI**	Baseline	1.30 (0.65, 2.05), 60	0.90 (0.50, 2.20), 54		
	Week 16	0.80 (0.20, 1.90), 60	0.90 (0.20, 1.50), 53		
**RCT**	Week 12	0.80 (0.20, 1.40), 55	0.75 (0.15, 1.50), 44	GMR[c]=1.07 (0.75, 1.51)	0.72
	Week 24	0.50 (0.10, 1.40), 43	0.75 (0.15, 1.65), 32	GMR[c]=0.95 (0.69, 1.30)	0.75
	Week 36	0.50 (0.10, 1.50), 37	0.60 (0.10, 1.60), 23	GMR[c]=0.85 (0.63, 1.15)	0.28
	Week 48	0.45 (0.10, 1.65), 32	1.05 (0.60, 1.90), 14	GMR[c]=0.76 (0.55, 1.03)	0.079
	Week 64	0.15 (0.00, 1.40), 26	1.20 (0.40, 1.70), 13	GMR[c]=0.65 (0.45, 0.93)	0.019
	Week 80	0.50 (0.20, 1.40), 15	0.70 (0.30, 1.60), 9	GMR[c]=0.56 (0.36, 0.86)	0.009
	Week 96	0.30 (0.20, 1.50), 9	0.90 (0.30, 2.70), 7	Not estimated	
	Week 112	0.30 (0.20, 3.60), 7	1.45 (0.60, 1.90), 6	Not estimated	
	Week 128	1.15 (0.25, 2.70), 4	1.45 (0.80, 2.35), 4	Not estimated	

		Randomised to adalimumab (n=60)		Randomised to placebo (n=55)		Treatment effect (95% CI)[b]	p-value
Test for treatment*time interaction							0.011
**SYMPTOMS OF SIDE EFFECTS SUMMARY SCORE[e]: Proportion with non-zero score (%)**							
**TRI**	Baseline	37/59 (62.7%)		29/54 (53.7%)			
	Week 16	33/60 (55.0%)		27/54 (50.0%)			
**RCT**	Week 12	22/55 (40.0%)		26/44 (59.1%)			
	Week 24	12/43 (27.9%)		16/32 (50.0%)			
	Week 36	15/37 (40.5%)		9/24 (37.5%)			
	Week 48	10/32 (31.3%)		5/14 (35.7%)			
	Week 64	8/26 (30.8%)		3/13 (23.1%)			
	Week 80	3/15 (20.0%)		3/9 (33.3%)			
	Week 96	2/9 (22.2%)		3/7 (42.9%)			
	Week 112	1/7 (14.3%)		2/6 (33.3%)			
	Week 128	2/4 (50.0%)		1/4 (25.0%)			
Test for treatment*time interaction							0.36
Overall treatment effect estimate						OR[f]=0.47 (0.17, 1.27)	0.14
**BEST CORRECTED VISUAL ACUITY[g]: Median (IQR), n**							
		**Left eye**	**Right eye**	**Left eye**	**Right eye**		
**TRI**	Baseline	85.0 (77.5, 89.5), 60	84.0 (79.0, 87.0), 59	84.0 (76.0, 89.0), 54	84.0 (77.0, 87.0), 54		

		Randomised to adalimumab (n=60)		Randomised to placebo (n=55)		Treatment effect (95% CI)[b]	p-value
	Week 16	84.0 (79.5, 89.5), 60	85.0 (79.5, 88.5), 60	85.0 (77.0, 90.0), 54	85.5 (79.0, 89.0), 54		
**RCT**	Week 12	85.0 (82.0, 89.0), 56	85.5 (80.5, 88.0), 56	84.0 (75.0, 89.0), 46	84.0 (80.0, 87.0), 46		
	Week 24	85.0 (81.0, 89.0), 43	86.0 (82.0, 88.0), 43	83.5 (78.5, 88.5), 32	84.5 (78.5, 88.5), 32		
	Week 36	87.0 (83.0, 90.0), 38	84.5 (82.0, 89.0), 38	82.5 (73.5, 87.5), 24	82.5 (74.5, 86.0), 24		
	Week 48	87.0 (82.5, 91.0), 32	86.0 (82.0, 89.0), 32	81.5 (77.0, 86.0), 14	83.0 (76.0, 90.0), 15		
	Week 64	85.0 (80.0, 89.0), 26	85.0 (81.0, 90.0), 26	82.0 (77.0, 88.0), 13	84.0 (74.0, 89.0), 14		
	Week 80	88.0 (84.0, 90.0), 15	85.0 (81.0, 89.0), 15	77.0 (74.0, 91.0), 9	84.0 (72.0, 87.0), 9		
	Week 96	88.0 (82.0, 89.0), 9	85.0 (80.0, 86.0), 9	82.0 (73.0, 91.5), 8	84.0 (70.5, 89.0), 8		
	Week 112	84.0 (80.0, 85.0), 7	84.0 (77.0, 89.0), 7	83.0 (72.0, 94.0), 6	86.5 (72.0, 90.0), 6		
	Week 128	84.5 (78.0, 86.5), 4	82.5 (76.0, 87.0), 4	78.0 (71.0, 88.5), 4	81.5 (54.0, 91.0), 4		
Test for treatment*time interaction							0.49
Overall treatment effect estimate						MD[h]=0.88 (-0.82, 2.57)	0.31

		Randomised to adalimumab (n=60)		Randomised to placebo (n=55)		Treatment effect (95% CI)[b]	p-value
**CENTRAL MACULAR THICKNESS[i]: Median (IQR), n**							
		**Left eye**	**Right eye**	**Left eye**	**Right eye**		
**TRI**	Baseline	287.0 (252.0, 318.5), 60	273.5 (249.0, 308.0), 60	286.0 (254.0, 302.0), 54	291.0 (261.0, 310.0), 54		
	Week 16	275.0 (245.5, 300.0), 60	265.5 (245.0, 303.0), 60	282.0 (251.0, 296.0), 55	282.0 (260.0, 298.0), 54		
**RCT**	Week 12	274.0 (238.0, 301.0), 56	263.0 (249.5, 304.0), 56	281.5 (243.0, 297.0), 46	277.0 (256.0, 298.0), 45		
	Week 24	265.5 (240.0, 292.0), 42	261.0 (239.0, 290.0), 42	286.0 (249.0, 301.0), 33	274.0 (260.5, 299.0), 32		
	Week 36	263.5 (240.0, 291.0), 38	258.5 (232.0, 286.0), 38	292.0 (250.0, 302.0), 25	272.0 (260.0, 307.0), 24		
	Week 48	272.5 (249.5, 297.5), 32	263.5 (239.5, 294), 32	287.0 (230.0, 297.0), 15	268.0 (255.0, 292.0), 15		
	Week 64	270.5 (242.0, 293.0), 26	263.0 (230.0, 298.0), 26	286.5 (253.0, 301.0), 14	269.0 (255.0, 297.0), 14		
	Week 80	258.0 (249.0, 286.0), 15	253.0 (221.0, 266.0), 15	283.0 (260.0, 302.0), 9	276.0 (264.0, 289.0), 9		
	Week 96	259.0 (249.0, 282.0), 9	257.0 (221.0, 260.0), 9	298.5 (268.0, 307.0), 8	279.0 (259.5, 312.5), 8		
	Week 112	255.0 (251.0, 277.0), 7	259.0 (214.0, 263.0), 7	289.5 (254.0, 301.0), 6	266.0 (257.0, 313.0), 6		

	Randomised to adalimumab (n=60)		Randomised to placebo (n=55)		Treatment effect (95% CI)[b]	p-value
Week 128	255.5 (252.0, 271.0), 4	258.5 (238.0, 264.5), 4	276 (232, 296.5), 4	259 (186.5, 289.5), 4		
Test for treatment*time interaction						0.42
Overall treatment effect estimate					MD[h,j]=-4.03 (-8.25, 0.19)	0.062

[a] A higher score indicates better quality of life

[b] MD = mean difference, GMR=geometric mean ratio, OR=odds ratio. All models also include data from unscheduled visits

[c] Joint model estimate: a Gaussian longitudinal model adjusting for baseline score and site size as fixed effects, patient as a random effect and time as a random slope, and a Weibull survival model for time to treatment failure

[d] A higher score indicates more vision related quality of life problems

[e] A non-zero score indicates patient has reported some medication side effects

[f] Joint model estimate: a Bernoulli longitudinal model adjusting for baseline score and site size as fixed effects, patient as a random effect and time as a random slope, and a Weibull survival model for time to treatment failure

[g] A higher score indicates better vision. Two patient-eyes (both placebo group) have been excluded from the table and analyses due to BCVA being 0 at all time points (including TRI entry), data from the other eye was included for both patients

[h] Joint model estimate: a Gaussian longitudinal model adjusting for baseline score, site size and time as fixed effects and patient-eye as a random effect, and a Weibull survival model for time to treatment failure

[i] A high value indicates poorer vision

[j] Removed 13 outlying measurements (2 Adalimumab – 2 patients, 11 Placebo – 4 patients) from the model to enable the model to fit: 5 measurements had very high standardised residuals due to high follow-up CMT measurements in relation to baseline and 8 had very low standardised residuals due to low follow-up CMT in relation to baseline.

Table ST11 Employment status

Change from baseline (TRI week 16) to last RCT visit[a]	Randomised to adalimumab (n=60)	Randomised to placebo (n=55)
**Net change in employment**	**-3 (-5.0%)**	**-1 (-1.8%)**
Gained employment	0 (0.0%)	2 (3.6%)
Lost employment	3 (5.0%)	3 (5.5%)
Stayed unemployed	15 (25.0%)	12 (22.8%)
Stayed employed	40 (66.7%)	34 (61.8%)
Change in percentage of work time missed because of problem associated with uveitis (Absenteeism); median (IQR)[b]	0.0 (0.0, 0.0)	0.0 (0.0, 0.0)
Change in percentage of impairment while working because of problem associated with uveitis (Presenteeism); median (IQR)[b,c]	0.0 (0.0, 10.0)	0.0 (-10.0, 10.0)
Change in percentage of overall work impairment; median (IQR)[b,c]	0.0 (0.0, 10.0)	0.0 (-10.0, 10.0)
Change in percentage of daily activity impairment; median (IQR)[b]	0.0 (-10.0, 10.0)	0.0 (-10.0, 10.0)

[a] 6 patients with missing data (2,4)

[b] Data only collected for participants who remained employed

[c] 11 patients with missing data (6,5).

Table ST12 Safety data by treatment received

System organ class term	No. of AEs	No. of SAEs	AEs/100 patient-years	SAEs/100 patient-years	No. of AEs	No. of SAEs	AEs/100 patient-years	SAEs/100 patient-years
**TRI**	**Received adalimumab (n= 202)**				**Received placebo (n=0)**			
**Number of patients with one or more AEs**	**116 (57.4%)**	**4 (2.0%)**						
**Any adverse event**	**276**	**5**	**356.1**	**6.5**				
Infections and infestations	50	1	64.5	1.3				
Immune system disorders	3	1	3.9	1.3				
Metabolism and nutrition disorders	1	0	1.3	0				
Psychiatric disorders	9	0	11.6	0				
Nervous system disorders	28	0	36.1	0				
Eye disorders	48	0	61.9	0				
Ear and labyrinth disorders	4	0	5.2	0				
Cardiac disorders	3	0	3.9	0				
Vascular disorders	6	0	7.7	0				
Respiratory, thoracic and mediastinal disorders	10	0	12.9	0				
Gastrointestinal disorders	31	2	40.0	2.6				
Hepatobiliary disorders	2	0	2.6	0				
Skin and subcutaneous tissue disorders	22	0	28.4	0				
Musculoskeletal and connective tissue disorders	17	1	21.9	1.3				

System organ class term	No. of AEs	No. of SAEs	AEs/100 patient-years	SAEs/100 patient-years	No. of AEs	No. of SAEs	AEs/100 patient-years	SAEs/100 patient-years
Renal and urinary disorders	3	0	3.9	0				
General disorders and administration site conditions	22	0	28.4	0				
Investigations	1	0	1.3	0				
Injury, poisoning and procedural complications	5	0	6.5	0				
Surgical and medical procedures	1	0	1.3	0				
Pregnancy, puerperium and perinatal conditions	1	0	1.3	0				
Covid-19	9	0	11.6	0				
**RCT**	**Received adalimumab (n= 59)**				**Received placebo (n=56)**			
**Number of patients with one or more AEs**	**33 (55.9%)**	**1 (1.7%)**			**37 (66.1%)**	**1 (1.8%)**		
**All adverse events**	**95**	**1**	**162.4**	**1.7**	**100**	**1**	**233.8**	**2.3**
Infections and infestations[a]	22	0	37.6	0	27	0	63.8	0
Neoplasms benign, malignant and unspecified (including cysts and polyps)	0	1	0	1.7	1	0	2.3	0
Metabolism and nutrition disorders	0	0	0	0	1	0	2.3	0
Psychiatric disorders	2	0	3.4	0	1	0	2.3	0
Nervous system disorders	5	0	8.5	0	3	0	7.0	0
Eye disorders	21	0	35.9	0	35	0	81.8	0

System organ class term	No. of AEs	No. of SAEs	AEs/100 patient-years	SAEs/100 patient-years	No. of AEs	No. of SAEs	AEs/100 patient-years	SAEs/100 patient-years
Ear and labyrinth disorders	0	0	0	0	1	0	2.3	0
Cardiac disorders	0	0	0	0	1	1	2.3	2.3
Vascular disorders	2	0	3.4	0	1	0	2.3	0
Respiratory, thoracic and mediastinal disorders	3	0	5.1	0	0	0	0	0
Gastrointestinal disorders	4	0	6.8	0	2	0	4.7	0
Hepatobiliary disorders	1	0	1.7	0	2	0	4.7	0
Skin and subcutaneous tissue disorders[b]	5	0	8.5	0	5	0	11.7	0
Musculoskeletal and connective tissue disorders	7	0	12.0	0	4	0	9.4	0
Renal and urinary disorders	0	0	0	0	1	0	2.3	0
General disorders and administration site conditions	5	0	8.5	0	4	0	9.4	0
Investigations	4	0	6.8	0	2	0	4.7	0
Injury, poisoning and procedural complications	5	0	8.5	0	4	0	9.4	0
Surgical and medical procedures	3	0	5.1	0	1	0	2.3	0
Covid-19	6	0	10.3	0	4	0	9.4	0

Table ST13 Comparisons of pre-specified groups of safety event rates

	Receive adalimumab		Received placebo		Incident rate ratio (95% CI)	p-value
	No. of AEs	AEs/100 patient-years	No. of AEs	AEs/100 patient-years		
Infections and infestations (including COVID-19)	28	47.9	31	73.2	0.70 (0.35,1.42)	0.33
Skin and subcutaneous tissue disorders	5	8.5	5	11.7	0.64 (0.17, 2.48)	0.52

Table ST14 Listing of possibly, probably or definitely related SAEs

	System organ class	SAE name	Expectedness	Period	Treatment received	Duration (days)	Relatedness	Intensity	Outcome
1	Immune system disorders	Allergies	Yes	TRI	Adalimumab	6	Definitely related	Mild	Resolved, no sequelae
2	Infections and infestations	Pneumonia	Yes	TRI	Adalimumab	2	Possibly related	Severe	Resolved, no sequelae
3	Musculoskeletal and connective tissue disorders	Musculoskeletal pain	Yes	TRI	Adalimumab	40	Probably related	Moderate	Resolved, with sequelae
4	Neoplasms benign, malignant and unspecified (including cysts and polyps)	Breast DCIS	Yes	RCT	Adalimumab	96	Possibly related	Moderate	Resolved, with sequelae

Table ST15 Longitudinal costs

		Randomised to adalimumab (n=60)	Randomised to placebo (n=55)	Treatment effect (95% CI)[b]	p-value
**Cost until:[a] £ Mean (SD), n**					
**RCT 1**	Week 12	433 (206), 58	251 (385), 53	MD = 280 (86, 474)	0.01
	Week 24	848 (301), 48	464 (463), 36	MD = 373 (117, 629)	<0.01
	Week 36	1239 (380), 43	647 (521), 29	MD = 497 (158, 836)	<0.01
	Week 48	1689 (392), 35	884 (609), 19	MD = 662 (211, 1134)	<0.01
	Week 64	2092 (455), 32	955 (271), 15	MD = 971 (305, 1637)	<0.01
	Week 80	2530 (569), 23	1094 (284), 15	MD = 1423 (432, 2413)	0.01
	Week 96	3206 (701), 13	1346 (371), 8	Not estimated	
	Week 112	3984 (812), 8	1539 (503), 6	Not estimated	
	Week 128	4488 (448), 6	1616 (403), 4	Not estimated	
Test for treatment*time interaction					0.61

[a] Joint model estimate: a gamma longitudinal model for costs (covariates: sex, baseline age, baseline EQ-5D utility, baseline better-seeing-eye visual acuity, time, patient random effect and random time slope) and a Weibull model for time to treatment failure

[b] MD = mean difference.

References

1. National Institute for Health and Care Excellence. NICE health technology evaluations: the manual. 31 January 2022. Last Updated Date 23 October 2025. https://www.nice.org.uk/process/pmg36/resources/nice-health-technology-evaluations-the-manual-pdf-72286779244741 (accessed 04 Dec 2025.
2. Pezzullo L, Streatfeild J, Simkiss P, Shickle D. The economic impact of sight loss and blindness in the UK adult population. *BMC Health Serv Res* 2018; **18**(1): 63.
3. Hazell M, Reeves B, Rogers CA, et al. Adalimumab vs placebo as add-on to Standard Therapy for autoimmune Uveitis: Tolerability, Effectiveness and cost-effectiveness-a protocol for a randomised controlled trial (ASTUTE trial). *BMJ Open* 2024; **14**(1): e082246.
4. NHS Business Services Authority. Prescription Cost Analysis – England – 2023/24. 2024. https://www.nhsbsa.nhs.uk/statistical-collections/prescription-cost-analysis-england/prescription-cost-analysis-england-202324 (accessed 17 July 2025).
5. NHS. Medicines information. 2023. https://www.nhs.uk/conditions/medicines-information/ (accessed 08 March 2024).
6. Jones K, H. W. Unit Costs of Health and Social Care 2023. https://kar.kent.ac.uk/105685/ (accessed 04 December 2025).
7. NHS Digital. National Cost Collection: National Schedule of NHS costs - Year 2023-24 - NHS trust and NHS foundation trusts. 2024. https://www.england.nhs.uk/costing-in-the-nhs/national-cost-collection/ (accessed 04 December 2025).
