# Supplementary material for: Adalimumab versus placebo as add-on to standard therapy for non-infectious uveitis in the United Kingdom: the ASTUTE randomised controlled trial with treatment run-in
Source: eClinicalMedicine. 2026 Oct 8;101:104240. doi: 10.1016/j.eclinm.2026.104240 (PMC13677017; doi:10.1016/j.eclinm.2026.104240)
Supplement: Supplementary file 3 [file mmc4.pdf]

Bristol Trials Centre
Bristol Medical School
University of Bristol
1-5 Whiteladies Road
Bristol. BS8 1NU

btc-mailbox@bristol.ac.uk
www.bristol.ac.uk/health-sciences/research/clinical-trials/

ASTUTE TRIAL

Adalimumab vs placebo as add-on to Standard Therapy for autoimmune Uveitis: Tolerability, Effectiveness and cost-effectiveness: a randomized controlled trial.

The ASTUTE trial – Statistical Analysis Plan

IRAS ID 271051
ISRCTN No ISRCTN31474800
EUDRACT no. 2020-000754-97
Sponsor no. OP/2017/6195
Funder no. 16/24/09

Role	Name	Signature	Date
Study Statistician	Mandy Lui (University of Bristol)		28/04/2025
Authorising Statistician	Katie Pike (University of Bristol)		27/03/2025
Chief Investigator	Andrew Dick (University of Bristol)		22nd April 2025
Co-Lead Clinical Investigator	Srilakshmi Sharma (Oxford University Hospitals NHS		22/04/2025

	Foundation Trust)		
Senior Methodologist	Barney Reeves (University of Bristol)		14/04/2025

Table of contents

1 Introduction 5
1.1 Summary of document 5
1.2 Background of study 5
1.2.1 Study rationale 5
1.2.2 Aims and objectives 5
2 Study methods 6
2.1 Design 6
2.2 Randomisation 6
2.3 Framework 6
2.4 Sample size 6
2.5 Masking 6
3 Populations 6
3.1 Study population 6
3.1.1 Inclusion criteria 7
3.1.2 Exclusion criteria 7
3.2 Data sources 8
3.3 Analysis populations 11
3.3.1 Intention-to-treat population 11
3.3.2 Per-protocol population 11
3.3.3 Safety population 11
3.3.4 Withdrawals 11
4 Statistical analyses and report content 11
4.1 General considerations 11
4.2 Outcomes 11
4.2.1 Responder criteria 11
4.2.2 Primary outcome 12
4.2.3 Secondary outcomes 12
4.3 Definitions and derivations 13
4.3.1 Primary outcomes 13
4.3.2 Secondary outcomes 13
4.3.3 Other variables 15
4.4 Analysis of the outcomes 16
4.4.1 Time frame for modelling 16
4.4.2 Adjustment in models 16
4.4.3 Analysis models 16
4.4.4 Accounting for different durations of follow-up in longitudinal outcomes 18
4.4.5 Model assumptions 18
4.5 General content 18

4.5.1 Participant flow 18
4.5.2 Baseline 18
4.5.3 Safety data 18
4.5.4 Sensitivity analyses 19
4.5.5 Subgroup analyses 19
4.5.6 Auxiliary analyses 19
4.6 Missing data and outliers 19
4.7 Multiple testing 20
5 References 20
6 Glossary 20
7 Revision History 21
8 Sample tables and figures 22

1 Introduction

1.1 Summary of document

The statistical analysis plan for the ASTUTE study has been written in accordance with Bristol Trials Centre (BTC) standard operating procedures (SOPs), the CONSORT statement, and International Conference on Harmonisation (ICH) Statistical Principles for Clinical Trials E9. The first version was prepared by Mandy Lui, Medical Statistician at BTC, University of Bristol, under the supervision of Katie Pike, senior statistician for the ASTUTE study, and covers all final statistical analyses to be performed as outlined in the study protocol.

1.2 Background of study

1.2.1 Study rationale

Autoimmune non-infectious, non-anterior uveitis (ANIU) is a term for several rare clinically defined phenotypic eye conditions in which the body's own immune system causes sight-threatening damage to the light sensitive retina at the back of the eye. Uveitis causes sight loss from inflammation inside the eye that may result in destruction of photoreceptors and retinal structure damage to blood vessels in the retina or leakage of fluid into the central, most sensitive area of the retina. Usual treatment includes induction of remission with high dose steroids and then maintenance with low dose steroids and one or two other drugs (immunmodulators) to reduce inflammation. However, many patients do not respond to usual treatment, or they need high dose steroid to control the disease. The trial arises because of the views of ophthalmologists that adalimumab is effective in a wider range of patients with ANIU than covered by the technology appraisal published by NICE and the drive from patients that there remains a reliance of steroids at unacceptable doses, despite use of immunomodulators.

1.2.2 Aims and objectives

To compare the effectiveness and cost-effectiveness of adalimumab when used to treat ANIU in either or both eyes in patients taking less than or equal to 5mg of steroids per day and other Immunomodulatory treatment (IMT) drugs, as required. Specifically, to test the hypothesis that adalimumab reduces the hazard of treatment failure (TF) in patients with ANIU, after weaning of oral prednisolone to <=5mg/day in a treatment run-in period (TRI). Participants in the trial progress through three phases: treatment run-in (TRI), randomised controlled trial (RCT) and open-label extension (OLE). This SAP relates only to analyses of data collected up to the end of the RCT.

Specific objectives are:

a) To estimate the hazard of TF in the group allocated to adalimumab compared with the group allocated to placebo.
b) To estimate differences between groups with respect to a range of secondary outcomes including: separate components of the composite TF outcome, visual function, BCVA, ocular and retinal signs of disease activity, health-related quality of life (HRQoL), resource use and costs, adverse events (AEs).
c) To estimate the cost-effectiveness of adalimumab compared to usual care.
d) To investigate associations between patient factors and ocular and retinal signs at the start of the TRI with responder status at 16 weeks, and to explore emerging participant phenotypes associated with TF during follow-up in the main trial.

Objectives c) and d) are not covered by this SAP. Objective c) will be included in a separate Health Economic Analysis Plan, and objective d) will be included in a separate SAP.

2 Study methods

2.1 Design

ASTUTE is a multi-centre, parallel group, placebo-controlled, pragmatic randomised controlled trial (RCT) with a TRI and OLE. Participants will be randomised on a 1:1 basis to receive either placebo or Adalimumab.

Consented and eligible participants will be enrolled into a 16-week TRI of active, open label adalimumab treatment. This will be reviewed at 4-weeks, 8-weeks and 16-weeks, allowing a 7 day visit window. Participants classified as 'responders' (see 4.2.1) who agree to continue into the RCT, will be randomized after assessment at the end of TRI.

Participants will be assessed for TF at each visit after randomisation (see 4.2.2). Follow-up visits will be scheduled at 12-week intervals up to 48 weeks and 16-week intervals thereafter (allowing -14 days and +7 days visit windows), consistent with usual care for patients with ANIU.

In the event of a TF in either eye, the participant will re-enter the TRI and be assessed at the 16-week visit. If the participant meets the criteria of 'responder' again, he/she will re-start the RCT but switch to the alternative allocated group to the group allocated at randomisation. If the participant does not respond, he/she will be withdrawn from the trial, without unmasking the allocation. A maximum of two switches will be allowed, i.e. in total there will be a maximum of three RCT phases for each participant.

2.2 Randomisation

Randomisation will be carried out electronically using a secure internet-based randomisation system by a member of the local research team. Block randomisation in varying block sizes of 2, 4 and 6 will be performed, in addition to stratification by centre.

2.3 Framework

The difference between the intervention groups will be compared according to a superiority framework.

2.4 Sample size

The planned sample size is 174 randomised participants. This sample size will have 90% power to detect a hazard ratio of 0.5 at 5% statistical significance (2-sided), assuming 27% in the placebo group survive free from TF 12 months after randomisation and allowing for 10% loss-to-follow-up in the first 12 months.

2.5 Masking

Participants, their clinical care team and all members of the research team except for the study statistician will be masked to allocation.

3 Populations

3.1 Study population

3.1.1 Inclusion criteria

- Participant is aged 18 years or over;
- Participant has: (a) active sight threatening ANIU (active inflammatory chorioretinal lesions OR abnormal central macular thickness (CMT) OR evidence of retinal vasculitis OR vitreous haze >0.5) and is being prescribed (already taking or being started on, if newly presenting with ANIU) oral prednisolone >5.0mg/day; OR (b) has controlled ANIU and is being prescribed oral prednisolone >5.0mg/day.
- Women must have a negative pregnancy test and be willing to use effective contraception for the duration of the participation in the trial and for 5 months after, or be surgically sterile or post-menopausal for >12 months.
- Participant is able to provide informed consent.

3.1.2 Exclusion criteria

- Participant has controlled ANIU and is maintained on oral prednisolone ≤5.0mg/day at the time of screening;
- Participant has systemic disease (whether associated with ANIU or not) that is being treated with steroids and requires >5mg/day oral prednisolone.
- Participant has untreated or active tuberculosis;
- Participant has severe infection, sepsis or opportunistic infection;
- Participant has uncontrolled glaucoma;
- Participant has multiple sclerosis;
- Participant is HIV positive;
- Participant has hepatitis B or hepatitis C;
- Participant has syphilis;
- Participant has Lyme disease;
- Participant has Behcet’s disease;
- Participant has toxoplasmosis chorioretinitis
- Participant has heart failure (NYHA III/IV);
- Participant has been diagnosed with cancer <5 years ago;
- Participant is undergoing monitoring for recurrence of cancer / tumour growth where their oncologist has concern that a TNFalpha inhibitor would be contraindicated;
- Participant is taking another biologic drug;
- Participant has taken an anti-TNF drug within the previous 90 days (anakinra and abatacept are contraindicated);
- Participant has had an Iluvien® implant within the previous 18 months and has controlled ANIU, or has had an Iluvien® implant within the previous 12 weeks regardless of whether ANIU is active or controlled;
- Participant has had an Ozurdex® implant, or an intravitreal steroid injection, or periocular steroid within the previous 12 weeks regardless of whether ANIU is active or controlled;
- Participant is pregnant;
- Participant has a known allergy or hypersensitivity to adalimumab or any of its excipients
- Participant is taking part in another interventional study;
- Participant has an epiretinal membrane likely to prevent an eye meeting response criterion at 16 weeks of central macular thickness <320um.

3.2 Data sources

A summary of data collection is shown below.

			Treatment run-in				RCT					
No.	Data item to be collected	Screen/baseline	1st injection (wk 0)	4 w	8 w (phone call)	16 w / Rx	12 w	24 w	36 w	48 w	Then:	Unsch. (*6)
1.	Confirm eligibility	✓				✓						
2.	Consent confirmed	✓				✓*[3]						
3.	Medical history	✓										
4.	Baseline characteristics	✓										
5.	Vital signs	✓				✓	✓	✓	✓	✓	16 w+	✓
6.	Weight	✓				✓		✓		✓	16 w+	✓
7.	History directed medical exam	✓				✓	✓	✓	✓	✓	16 w+	✓
8.	Full blood count (FBC)	✓		✓		✓	✓	✓	✓	✓	16 w+	✓
9.	Liver function tests	✓		✓		✓	✓	✓	✓	✓	16 w+	✓
10.	Electrolytes profile	✓		✓		✓	✓	✓	✓	✓	16 w+	✓
11.	Glucose test	✓		✓								
12.	TB IGRA test	✓										
13.	Chest X-ray	✓										
14.	12-lead ECG*[1]	✓										
15.	MRI*[1]	✓										
16.	Syphilis test	✓										
17.	HIV test	✓										
18.	Hep B / Hep C test	✓										
19.	Lyme IgG/IgM Antibody Serology*[1]	✓										
20.	Varicella history	✓										
21.	Pregnancy test (women only)	✓										
22.	BCVA	✓		✓		✓	✓	✓	✓	✓	16 w+	✓
23.	OCT	✓				✓	✓	✓	✓	✓	16 w+	✓

			Treatment run-in				RCT					
**No.**	**Data item to be collected**	**Screen/baseline**	**1st injection (wk 0)**	**4 w**	**8 w** (phone call)	**16 w / Rx**	**12 w**	**24 w**	**36 w**	**48 w**	**Then:**	**Unsch.** (*6)
24.	Fundus colour imaging	✓				✓	✓	✓	✓	✓	16 w+	✓
25.	Autofluorescence (AF) imaging	✓				✓*2	✓*2	✓*2	✓*2	✓*2	16 w+*2	✓*2
26.	Fundus fluorescein angiogram (FFA)	✓				✓*2	✓*2	✓*2	✓*2	✓*2	16 w+*2	✓*2
27.	Clinical exam (including slit-lamp examination and indirect ophthalmoscopy)	✓		✓								
28.	Clinical exam for treatment failure (including slit-lamp examination and indirect ophthalmoscopy)					✓	✓	✓	✓	✓	16 w+	✓
29.	Telephone wellbeing review				✓							
30.	Adverse events			✓	✓	✓	✓	✓	✓	✓	✓	✓
31.	EQ-5D-5L	✓				✓	✓	✓	✓	✓	16 w+	✓
32.	VCM1	✓				✓	✓	✓	✓	✓	16 w+	✓
33.	Symptoms of side-effects	✓				✓	✓	✓	✓	✓	16 w+	✓
34.	WPAI-SHP questionnaire	✓				✓	✓	✓	✓	✓	16 w+	✓
35.	Blood sample (for serum)*4	✓				✓	✓*4	✓*4	✓*4	✓	48 w+*4	✓*4
36.	Blood sample (for DNA)	✓										
37.	Resource use					✓	✓	✓	✓	✓	16 w+	✓

*1 Only required if clinically indicated.
*2 Only required if undertaken at baseline to support the clinical decision about TF .
*3 Written informed consent will be obtained at the start of the TRI; this consent will be confirmed among responders at the start of the RCT.
*4 Only taken at these time points in the event of treatment failure being detected.
*5 If patient has no history of varicella, advise to avoid people with chickenpox or shingles for duration of time on study medication.
*6 If TF is suspected in between RCT follow up visits the participant should attend an unscheduled visit where the research team must complete all assessments as per scheduled RCT follow-up visits. IF TF is confirmed not to have occurred participants must still attend the next scheduled visit and all assessments conducted as per protocol.

BCVA – best corrected visual acuity; **TF** – treatment failure; **OCT** – optical coherence tomogram; **Rx** – Randomisation; **VCM1** – Visual Function Questionnaire; **WPAI-SHP** – Work Productivity and Activity Impairment Questionnaire: Specific Health Problem Questionnaire; **w** - weekly; **w+** - weekly thereafter

The data items are collected from difference source:

- Items 1-22, 27-30 and 37: paper CRFs designed specifically to capture the required data for the study (item 37 prescribed medication source data is the eCRF);
- Items 23-26: OCT, fundus, autofluorescence and fundus fluorescein angiography images submitted for independent grading;
- Items 31-34: Completed questionnaires returned by participants;
- Items 35-36: not applicable; samples for storage only (for future research).
- IMP accountability: IMP-Track Investigational Medicinal Product Management System

3.3 Analysis populations

3.3.1 Intention-to-treat population

All summaries and analyses of the primary and secondary outcomes (except for the safety outcome) will be conducted on the intention-to-treat (ITT) population. The ITT population will consist of all randomised participants grouped according to treatment allocated, regardless of whether they are ineligible or were treated according to the randomised allocation or not.

3.3.2 Per-protocol population

The following types of protocol deviations will be defined:

- Participant did not meet the study eligibility criteria but was treated in the study
- Participant did not receive allocated intervention

1. Participant failed to take at least 70% of trial medication in the first TRI and RCT. Note, by definition this would capture participants who had a significant pause (accounting for, in total, >30% of trial medication, cumulative over multiple pauses if applicable)-.

Participants who are considered a deviation by at least one of these criteria will be excluded from the per-protocol population and resultant sensitivity analysis (see section 4.5.4).

3.3.3 Safety population

The safety population will consist of all randomised participants. Participants will be grouped according to the treatment received and presented by trial periods due to treatment crossover.

3.3.4 Withdrawals

Participants can withdraw/be withdrawn from treatment and/or follow-up at any stage of the study at their own discretion or that of the treating clinician. All participants withdrawn from the study, in addition to those found to be ineligible post-randomisation, will be continued to followed up until the end of the study. All data previously collected by the participant will be used in the analyses.

4 Statistical analyses and report content

4.1 General considerations

The statistical analysis is the responsibility of the BTC study statistician.

Where interventions are being compared formally using statistical modelling, the placebo group will act as the reference category. All applicable statistical tests will be 2-sided and will be performed using a 5% significance level, with the exception of tests for interactions that will be performed using a 10% significance level due to the reduced power of such tests.

4.2 Outcomes

4.2.1 Responder criteria

A responder will be defined as meeting **all** of the following criteria in both eyes at the 16 week TRI visit:

i. No activity on colour fundus or autofluorescence imaging indicative of inflammatory chorioretinal lesions;
ii. Central subfield thickness (CMT) ≤ 320μm on any OCT machine and no evidence from OCT of cystoid macular oedema (CMO);

iii. No evidence of active retinal vasculitis;
iv. ≤ 0.5 vitreous haze Binocular Indirect Ophthalmoscopy (BIO) score;
v. Prescription of ≤ 5.0mg/day oral prednisolone to achieve/maintain uveitis remission (participant is a responder if prescribed >5.0mg/day oral prednisolone for a co-morbidity (e.g. rheumatic disease) where ALL of **i-iv** are met).

4.2.2 Primary outcome

The primary outcome is time to the first TF for a participant. Any of the following criteria in one or both eyes, where applicable, will constitute TF:

i. >=15 letter decrease in BCVA, compared to BCVA measured by an optometrist masked to treatment allocation at the 16 week TRI visit;
ii. new active inflammatory chorioretinal lesions (masked review of fundus colour and autofluorescence images by clinician);
iii. >20% increase in CMT, compared to CMT at the 16 week TRI timepoint (CMT is a parameter that is measured by an automated algorithm when doing OCT);
iv. onset or worsening of retinal vasculitis (masked clinician assessment of fundus fluorescein angiogram);
v. 2 step worsening of vitreous haze compared to score at the 16-week TRI visit (masked clinician assessment);
vi. prescription by a masked clinician of >5mg/day oral prednisolone to maintain uveitis remission (i.e. to avert relapse before any of the above criteria for manifest active disease (**i-v**)).

Prescription of >5mg/day oral prednisolone for a co-morbidity (e.g. rheumatic disease) (**vi**) but with none of **i-v** being present does not constitute TF.

If the participant experiences any of criteria **i-vi** within 14 days of having intraocular surgery, it does not constitute TF. If the criteria persists beyond 14 days then it does constitute TF.

4.2.3 Secondary outcomes

The secondary outcomes include:

1. Individual TF components, assessed at each trial visit;
2. Retinal morphology (OCT; macular and retinal nerve fibre layer), assessed at each trial visit;
3. AEs, assessed at each trial visit;
4. EQ-5D-5L questionnaire at the start of TRI, at 16 weeks immediately before randomisation, then 12-weekly after randomization up to week 48 and 16-weekly thereafter;
5. Patient-reported symptoms of side-effects at the start of TRI, at 16 weeks (immediately before randomisation), then at each trial visit after randomisation and at any interim attendance prompted by an AE;
6. Patient-reported visual function, at the start of TRI, at 16 weeks immediately before randomisation, 12-weekly up to week 48 and 16 weekly thereafter;
7. Best corrected visual acuity (BCVA) assessed at each trial visit;
8. Employment status at the start of TRI, at 16 weeks immediately before randomisation, 12-weekly up to week 48 and 16 weekly thereafter;
9. Resource use at the start of TRI, at 16 weeks immediately before randomisation, and every 12 weeks (up to week 48) or 16 weeks (after week 48) during follow-up after randomisation.

Note outcome 9 (resource use) will be included in the Health Economics Analysis Plan (HEAP) and therefore is not covered in this SAP.

4.3 Definitions and derivations

4.3.1 Primary outcomes

New variable	Rules
Treatment failure	**YES:** if any of the following criteria in one or both eyes:<br>i. >= 15 decrease in BCVA, compared to the 16 week TRI visit;<br>ii. New active inflammatory chorioretinal lesions;<br>iii. > 20% increase in CMT, compared to the 16 week TRI visit;<br>iv. Onset or worsening of retinal vasculitis;<br>v. 2-step worsening of vitreous haze compared to score at the 16-week TRI visit;<br>vi. Prescription of >5mg/day oral prednisolone to maintain uveitis remission;<br>**NO:** if the participant does not meet the above criteria (including Fundus Fluorescein Angiogram (FFA) not done for **iv**) OR meet **vi** for a co-morbidity (e.g. rheumatic disease) but with none of **i-v** OR if the participant experiences any of criteria **i-vi** within 14 days of having intraocular surgery<br>**Missing:** otherwise
Time to first treatment failure (days)	= (visit date – randomisation date) if patient had TF<br>= (date of last follow-up – randomisation date) if patient did not have TF before the end of follow-up<br>= (date of withdrawal – randomisation date) if patient did not have TF and withdrew before the end of follow-up and stated they did not wish to continue follow-up visits
Censor variable	**YES:** if patient did not have TF before the end of follow-up<br>**No:** if patient had TF<br>**Missing:** otherwise

4.3.2 Secondary outcomes

All the secondary outcomes are measured at multiple time points: baseline, training visit (AEs only), the start of TRI, the end of TRI (week 16) and each visit after randomisation (including unscheduled visits).

1. **Individual TF components;**
   The six TF components outlined in section 4.3.1 will be derived for each RCT visit.

2. **Retinal morphology (OCT; macular and retinal nerve fibre layer);**
   Intact OCT macula and OCT retinal nerve fibre layer will be provided directly from the source data.

3. **AEs;**
   MedDRA System Organ Classes (SOCs) and Preferred Terms will be derived for all events using the most recent version of MedDRA at the time of analysis. Incidence rates of AEs and SAEs in the following categories will be calculated across all time patients are exposed to Adalimumab/placebo:

New variable	Rules
Incidence rate of AEs/SAEs classified as "Infections and infestations" or "COVID-19" (in 100 patient-years)	Number of AEs (or SAEs) classified as "Infections and infestations" or "COVID-19" / (Total time of patients are exposed to treatment in years * 100)
Incidence rate of AEs/SAEs classified as "Skin and subcutaneous tissue disorders" (in 100 patient-years)	Number of AEs (or SAEs) classified as "Skin and subcutaneous tissue disorders" / (Total time of patients are exposed to treatment in years * 100)

* Excluding COVID-19 (on CRFs COVID-19 is classified as "Other")

4. **EQ-5D-5L;**
The EQ5D summary score will be derived as follows:

New variable	Rules
EQ5D single summary index score	Five digit 'state' score is derived as:<br><br>*10000*mobility score + 1000*self-care score + 100*usual activities score + 10*pain/discomfort score + anxiety/depression score.*<br><br>Each 5L state score is assigned a single summary index score by mapping to the 3L valuation set according to the most current NICE guidelines at the time of analysis. Index scores are numerical and range from -0.594 to 1.00, with a score of 1.00 denoting perfect health. Where required, deaths will be rated as a score of zero as per the EQ-5D-3L user guide.

The EQ5D VAS score will be provided directly from source data (i.e. no further derivations are required).

5. **Patient-reported symptoms of side-effects;**
The medication side effect questionnaire includes a leading question and 4 questions (bothersome of side effects, interference with physical function, interference with mental function and interference on overall satisfaction). Details of how to derive a summary score are described below.[1]

New variable	Rules
Summary score for medication side effects questionnaire	**At each timepoint:**<br>= mean of questions 2-5, if all non-missing and question 1 = YES<br>= 0, if question 1 = NO<br>**MISSING:** otherwise

6. **Patient-reported visual function;**
   The visual function quality of life questionnaire (VCM1) consists of 10 questions (embarrassment, anger, loneliness, depression, fear of deterioration in vision, safety at home, safety outside the home, coping with daily life, inability to do preferred activities and life-interference), ranging from 0 (not at all) to 5 (all the time). Details of how to derive a single score are described below.[2]

**New variable**	**Rules**
Summary score for VCM1	**At each timepoint:**<br>= mean of questions 1 – 10, if all non-missing<br>**MISSING:** otherwise

7. **Best corrected visual acuity (BCVA);**
   BCVA of study eye(s) will be determined using validated Early Treatment Diabetic Retinopathy Study (ETDRS) vision charts, by an optometrist masked to treatment allocation. A zero BCVA value indicates worse visual acuity, and 100 best.

**New variable**	**Rules**
BCVA at standard luminance	**At each timepoint:**<br>= ETDRS letters read at standard luminance in study eye if ETDRS letters read at standard luminance not missing; OR<br>= 0, if ETDRS letters read at standard luminance in study eye = 0 AND count fingers; OR<br>= -15, if ETDRS letters read at standard luminance in study eye = 0 AND hand movements; OR<br>= -30, if ETDRS letters read at standard luminance in study eye = 0 AND perception of light; OR<br>= -45, if ETDRS letters read at standard luminance in study eye = 0 AND (no perception of light OR artificial eye); OR<br>ELSE<br>**MISSING:** otherwise

8. **Employment status**
   Participants completed the work productivity and activity impairment questionnaire (WPAI:SHP).

4.3.3 Other variables

**New variable**	**Rules**
Subgroup analysis categories: eligibility for adalimumab under current NICE technology appraisal (TA460)[3]	**Eligible:** if **ALL** of criteria 1-4 are met:<br>1. Oral prednisolone (CRF C4) dose >7.5mg<br>2. Is uveitis active (CRF B5) = YES **AND** 'Features of active disease' (CRF B7) = YES for something other than 'Macular oedema' in **at least one eye**<br>3. At least one of the following medications (CRF C4) is TRUE:<br>a. Methotrexate<br>b. Mycophenolate motefil<br>c. Tacrolimus<br>d. Azathiroprine

	e. Other: cyclosporin<br>**4. EITHER:**<br>a. Is uveitis active (CRF B5) = YES or NO for **both** eyes **AND** 'Features of active disease' (CRF B7) = YES for something other than 'Macular oedema' for **both** eyes<br>**OR**<br>b. (Intermediate uveitis with systemic disease = YES **OR** Posterior uveitis with systemic disease = YES **OR** Panuveitis with systemic disease other than VKH or Behcet = YES (CRF B5)) **AND** disease listed has been approved by Chief Investigator<br>**OR**<br>c. Is uveitis active (CRF B5) = YES or NO for **one** eye **AND** for the opposite eye BCVA (CRF B6) = AE/CF/HM/PL/LP OR <51<br>**Ineligible:** otherwise

4.4 Analysis of the outcomes

4.4.1 Time frame for modelling

All outcomes will only be formally compared for the first RCT period (i.e. until the first TF, or the patient withdraws from the study/completes follow-up). Outcomes for subsequent time periods will be described but not formally compared, except for within an additional within-patient analysis (see section 4.5.6).

4.4.2 Adjustment in models

For continuous and binary outcomes, centre will be fitted as a random effect where possible. Time to event models will adjust for centre using a shared frailty term, if possible. If the shared frailty term for centre leads to convergence issues, time to event models will be stratified by centre size.

If the frequency of the outcome is insufficient to allow estimation of regression coefficients for all these variables (e.g. rare binary outcomes), the variables with coefficients that cannot be estimated will be omitted.

The ED-5D-5L, medication side effects, visual function and BCVA outcomes are collected at baseline, at the start of TRI, at end of TRI (week 16) and at each visit post-randomisation for all participants. The data collected at the 16 week TRI visit will be used as a covariate if available, otherwise missing values will be imputed (see section 4.6) and subsequent values after randomisation will be modelled (see section 4.4.3).

4.4.3 Analysis models

All outcomes listed in the study protocol will be presented. General methods of presentation and assessing treatment effects are outlined below.

Most outcomes will be analysed at the patient level; e.g. TF in either eye will be considered a primary outcome event. Exceptions will be retinal morphology and BCVA outcomes, which will be analysed at the eye level. For these outcomes, summary statistics will be reported separately for each eye (left and right). However, models will include data from both eyes, an "eye" fixed effect will be included allowing for

correlation within patients and the treatment effect will be estimated and reported averaging across both eyes.

Each outcome will be considered under a certain data type, as outlined in the table below:

Data type	Outcomes
Time to event	Time to first TF (primary outcome)
Continuous longitudinal	EQ-5D-5L (secondary outcome 4): utility score and visual analogue scale (VAS) score<br>Medication side effects (secondary outcome 5): summary score<br>Visual function (secondary outcome 6): summary score<br>BVCA (secondary outcome 7)
Binary longitudinal	Retinal morphology (secondary outcome 2): intact OCT macular and OCT retinal nerve fibre layer
Incidence rate	AEs/SAEs (secondary outcome 3): incidence across two categories
Descriptive outcomes	Individual TF components (secondary outcome 1)<br>AEs/SAEs (secondary outcome 3): individual events<br>Employment status (secondary outcome 8): change in employment status from baseline to final visit

Each data type will be summarised and compared between the groups according to the following:

- **Time to event outcomes** will be summarised by the median and IQR in each treatment group, estimated from survival modelling. Outcomes will be compared using Cox's proportional hazards models. If the assumptions of the Cox proportional hazard model are violated, alternative methods, such as restricted mean survival time or parametric models will be used as appropriate. Treatment comparisons will be presented as hazard ratios (HRs) and 95% confidence intervals (CIs).

- **Continuous longitudinal outcomes** will be summarised as means and SDs (or medians and IQRs if distributions are skewed) at each time point. Outcomes will be compared using linear mixed effects methodology. Due to the outcomes being measured at a number of time points, including some unscheduled visits, time will be fitted continuously, with various parametrisations of time explored. The final model will be selected based on the best fit. Estimated treatment effects will be presented at the time of each study visit in the first RCT.

- **Binary longitudinal outcomes** will be presented as numbers and percentages of patients at each time point by treatment group. Outcomes will be compared between treatment groups using mixed effects logistic regression models, using the same modelling strategy as outlined for continuous longitudinal outcomes (i.e. time will be fitted continuously). Treatment estimates will be presented as adjusted incidence rate ratios (IRR) with 95% CIs at the time of each study visit in the first RCT. Formal statistical comparisons of treatment effects will only be performed if more than ten patients in total experience the outcome (with at least one event in each treatment group).

- **Incidence rate outcomes** will be presented as means and SDs, Outcomes will be compared using Poisson models. Treatments estimates will be presented as adjusted incidence rate ratios (IRR) with 95% CIs.

- **Descriptive outcomes** will be simply summarised by treatment group as means and SDs (or medians and IQRs if distributions are skewed) for continuous data and numbers and percentages of patients for categorical data.

4.4.4 Accounting for different durations of follow-up in longitudinal outcomes

All longitudinal outcomes (continuous, binary and incidence rate) will be modelled jointly with time to first treatment failure; this analysis technique takes into account different follow-up times across participants. If joint models do not converge other methods will be explored.

Sensitivity analyses will be performed to assess the robustness of this approach (see section 4.5.4).

4.4.5 Model assumptions

For all methods outlined underlying assumptions will be checked using standard methods, e.g. residual plots, tests for proportional hazards, etc. If assumptions are not valid then alternative methods of analysis will be sought. If outlying observations are found which mean models do not fit the data adequately, such observations will be excluded from the main analyses and comments made in footnotes. Sensitivity analyses may be performed to examine the effect on the conclusions of the study of excluding outlying observations.

4.5 General content

4.5.1 Participant flow

The CONSORT flow diagram will be used to summarise the flow of participants through screening until the third follow-up period of the study. Protocol deviations defined in section 3.3.2 will be summarised by treatment group. The number of withdrawals will be presented, along with reasons for withdrawal.

4.5.2 Baseline

Baseline data (i.e. patient demography and past history) will be described by treatment group for patients in the analysis population. Continuous variables will be summarised using the mean and standard deviation (SD) (or median and inter quartile range (IQR) if the distribution is skewed), and categorical data will be summarised as a number (n/N) and percentage.

Baseline data will also be described by TRI responder status for all patients who consent to the TRI.

4.5.3 Safety data

As described in section 4.4.3, safety data will be described for the safety population, i.e. by treatment received (section 3.3.3). Tabulations will be performed for each TRI/RCT period. However an overall tabulation will be done of the total time period a participant received Adalimumab/placebo (including TRI periods). Secondary outcome 3 (incidence rate of AEs/SAEs) provides a formal comparison of safety events.

4.5.4 Sensitivity analyses

Primary outcome

- A per-protocol sensitivity analysis of the primary outcome will be performed by performing the primary outcome analysis on the per-protocol population (section 3.3.2), to investigate the impact of protocol deviations on the intervention.
- There are two types of censoring for the primary outcome: a) due to participant withdrawal, b) due to the trial ending. We do not anticipate informative censoring problems due to the latter, however, if the number of participant withdrawals (prior to TF) are significant (>10%), we will investigate potential informative censoring by imputing values for participant withdrawals.

Secondary outcomes

Sensitivity analyses will be performed for longitudinal outcomes to assess the robustness of the approach used to account for differing lengths of follow-up. Methods such as imputation via inverse probability censoring weighting and/or treatment switching models (utilising data from the entire trial period) will be explored. These methods are considered exploratory.

4.5.5 Subgroup analyses

A subgroup analysis (that is not pre-specified in the study protocol) is planned for the primary outcome comparing participants who are eligible to receive adalimumab under the current NICE technology appraisal (TA460)[3] with those who are ineligible. These subgroup effects will be investigated by adding subgroup*treatment interaction terms to the models already described and presenting treatment effect estimates in each subgroup on a forest plot. This subgroup analysis will have very low power and therefore the interaction term is unlikely to reach statistical significance. However, it may result in indications of any differences between those enrolled as active vs in drug-induced remission but on >5mg prednisone. No further subgroup analyses are planned.

4.5.6 Auxiliary analyses

To investigate within-subject comparisons of the primary and secondary outcomes between adalimumab and placebo, additional analyses will be performed including outcomes from all TRI and RCT periods (i.e. all follow-up periods after randomisation). Such analyses will use the point of randomisation into the first RCT phase as the time origin and other covariates (i.e. treatment and baseline variables) will be updated when participants switch to different TRI/RCT phases.

Treatment effect estimates for primary and secondary outcomes will be updated including data from the OLE. These analyses are considered auxiliary analyses and may be biased as participants and clinicians will be unmasked when OLE data are collected. Therefore these estimates are **not** considered the primary treatment effect estimates.

4.6 Missing data and outliers

A thorough data cleaning process will be carried out and attempts will be made to obtain any missing data by chasing until it is either received or confirmed as not available.

In all tables missing data for continuous variables will be indicated by footnotes. For categorical variables, missing data will be highlighted by use of observation counts. If the amount of missing data differs substantially between treatment groups potential reasons will be explored.

Missing predictors:
There will be no missing data for any of the randomisation factors (by design). All other potential predictors are pre-randomisation measurements of continuous longitudinal outcomes. If missing for fewer than 5% of participants, the missing values will be imputed with mean values for the cohort as a whole. If missing for 5% or more then multiple imputation will be considered.

Missing longitudinal data:
The assumption of data missing at random (MAR) will be examined by comparing the variances of the continuous outcomes across the groups and the withdrawals/dropouts in each group; if similar, data can be assumed to be MAR[4]. It is anticipated that outcomes are more likely to be missing if the participant has a ‘poor’ outcome but it is not expected that missingness will be related to treatment group. If the MAR assumption does not hold and dropout/missingness is not balanced across groups, imputation methods which do not assume MAR will be considered.[5]

4.7 Multiple testing

No formal adjustment will be made for multiple testing for all outcomes. However, as previously described formal statistical comparisons will not be made for outcomes with low event rates and no treatment effects will be estimated for RCT periods 2 and 3. Consideration will be given when interpreting the results of the number of statistical tests performed and the consistency, magnitude, and direction of treatment estimates for different outcomes.

5 References

1. Ruiz MA, Pardo A, Rejas J, Soto J, Villasante F, Aranguren JL. Development and validation of the "Treatment Satisfaction with Medicines Questionnaire" (SATMED-Q). Value Health. 2008 Sep-Oct;11(5):913-26. doi: 10.1111/j.1524-4733.2008.00323.x. Epub 2008 May 20. PMID: 18494753.
2. Frost NA, Sparrow JM, Durant JS, Donovan JL, Peters TJ, Brookes ST. Development of a questionnaire for measurement of vision-related quality of life. Ophthalmic Epidemiol. 1998 Dec;5(4):185-210. doi: 10.1076/opep.5.4.185.4191. PMID: 9894804.
3. https://www.nice.org.uk/guidance/ta460/chapter/1-Recommendations
4. Treatment group outcome variance difference after dropout as an indicator of missing-not-at-random bias in randomized clinical trials. (2022). https://www.medrxiv.org/content/10.1101/2022.04.15.22273918v1 [Accessed 21 Feb.2023].
5. Tompsett DM, Leacy F, Moreno-Betancur M, Heron J, White IR. On the use of the not-at-random fully conditional specification (NARFCS) procedure in practice. Stat Med. 2018 Jul 10;37(15):2338-2353. doi: 10.1002/sim.7643. Epub 2018 Apr 2. PMID: 29611205; PMCID: PMC6001532.

6 Glossary

ANIU	Autoimmune non-infectious uveitis
AE	Adverse event - any undesirable event in a subject receiving treatment according to the protocol, including occurrences which are not necessarily caused by or related to administration of the research procedures.
ASTUTE	Adalimumab vs placebo as add-on to Standard Therapy for autoimmune Uveitis: Tolerability, Effectiveness and cost-effectiveness. The ASTUTE pragmatic randomized controlled trial.
BCVA	Best corrected visual acuity
BTC	Bristol Trials Centre

CI	Confidence interval
CMT	Central macular thickness
CRF	Case report form
DMSC	Data monitoring and safety committee
EQ-5D-3L	EuroQol-5 Dimensions, 3-level version
EQ-5D-5L	EuroQol-5 Dimensions, 5-level version
FFA	Fundus Fluorescein Angiogram
GMR	Geometric mean ratio
HR	Hazard ratio
HRQoL	Health-related quality of life
ICH	International Conference on Harmonisation
IMT	Immunomodulatory treatment
IRR	Incidence rate ratio
ITT	Intention-to-treat
IQR	Inter quartile range
MAR	Missing at random
MD	Mean differences
OCT	Optical coherence tomography
RCT	Randomised controlled trial
SAE	Serious adverse event - events which result in death, are life threatening, require hospitalisation or prolongation of hospitalisation, result in persistent or significant disability or incapacity.
SD	Standard deviation
SOC	System Organ Class
TF	Treatment failure
TRI	Treatment run-in – Participant will receive open label Adalimumab for 16 weeks. Different treatment run-in periods will be presented as TRI1, TRI2 and TRI3.
VCM1	Visual function quality of life questionnaire
WPAI:SHP	Work productivity and activity impairment questionnaire: specific health problem

7 Revision History

Version number	Revision date	Justification for revision
2.0		• Addition of analysis of data from TRI and OLE periods<br>• Clarification of how data from participants who withdraw will be used in derivation of the primary outcome<br>• Addition/clarification of sensitivity analyses to assess robustness of analysis approaches used<br>• Removal of formal comparison of safety events classified as “other” (events are now planned to be MedDRA coded and included in correct category)

8 Sample tables and figures

Example tables and figures are given below to indicate how data will be summarised and presented. **Please not these are not presented for all planned analyses**; they are intended to be an indication only and are not exhaustive.

Figure F1 Flow of participants

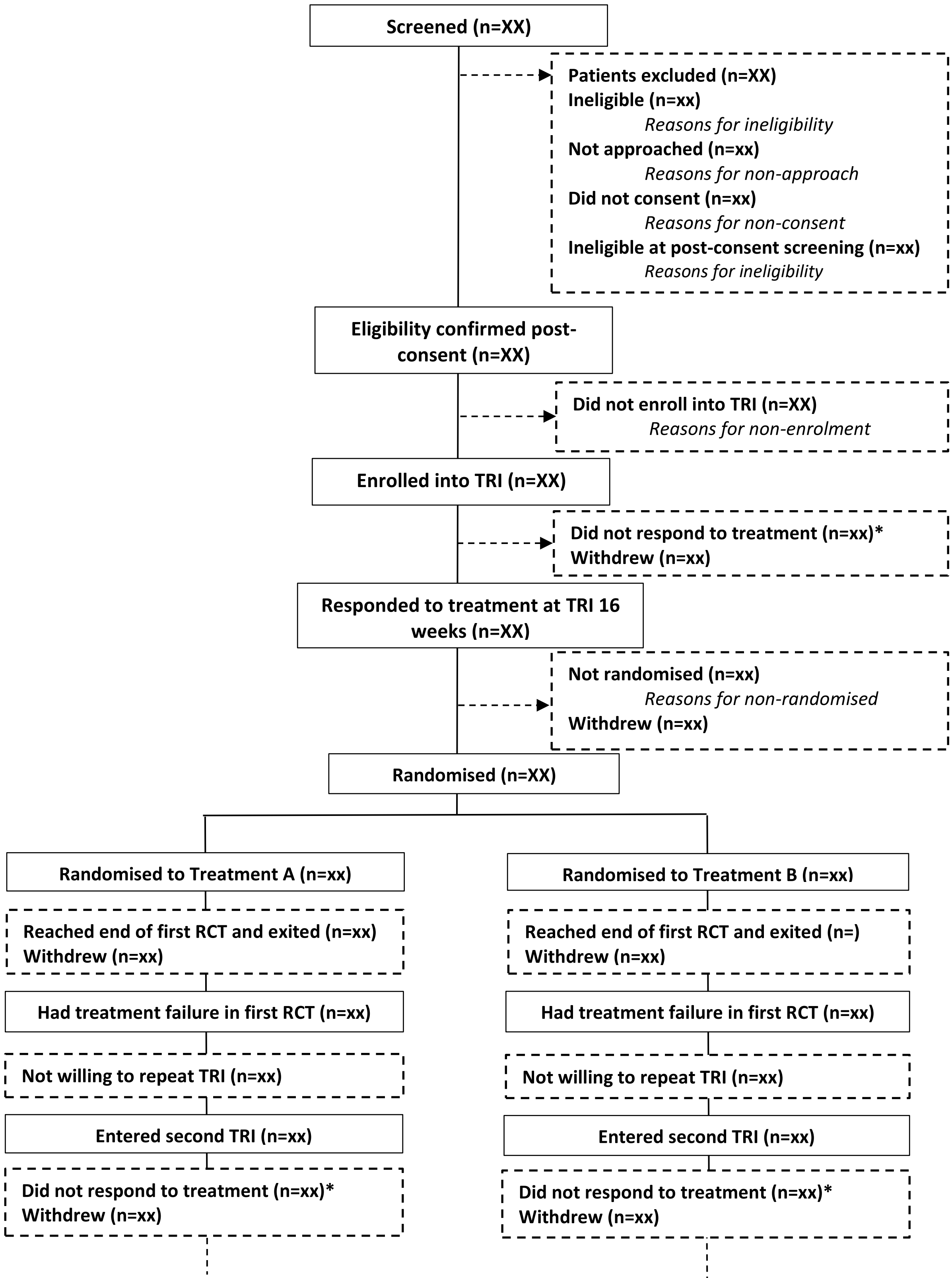

Figure F1 Flow of participants

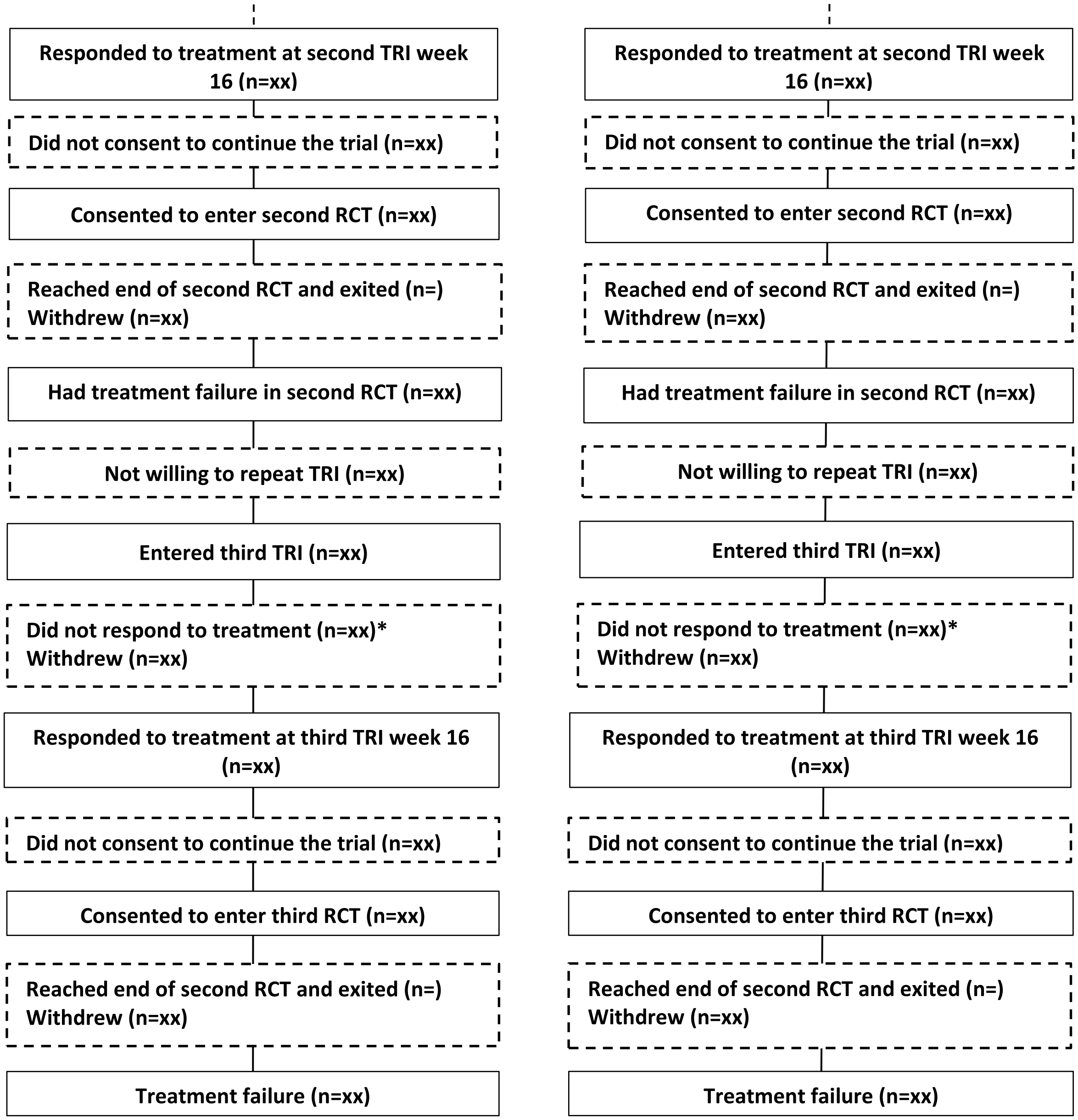

*Responder components will be described in separate table.

Table T1 Primary outcome

Time to treatment failure (days)	Randomised to Adalimumab (n=XX)	Randomised to placebo (n=XX)	Hazard Ratio (95% CI)	p-value
Median (IQR)				

Notes:
Data are only included from RCT period #1

Figure F1 Kaplan-Meier plot for primary outcome

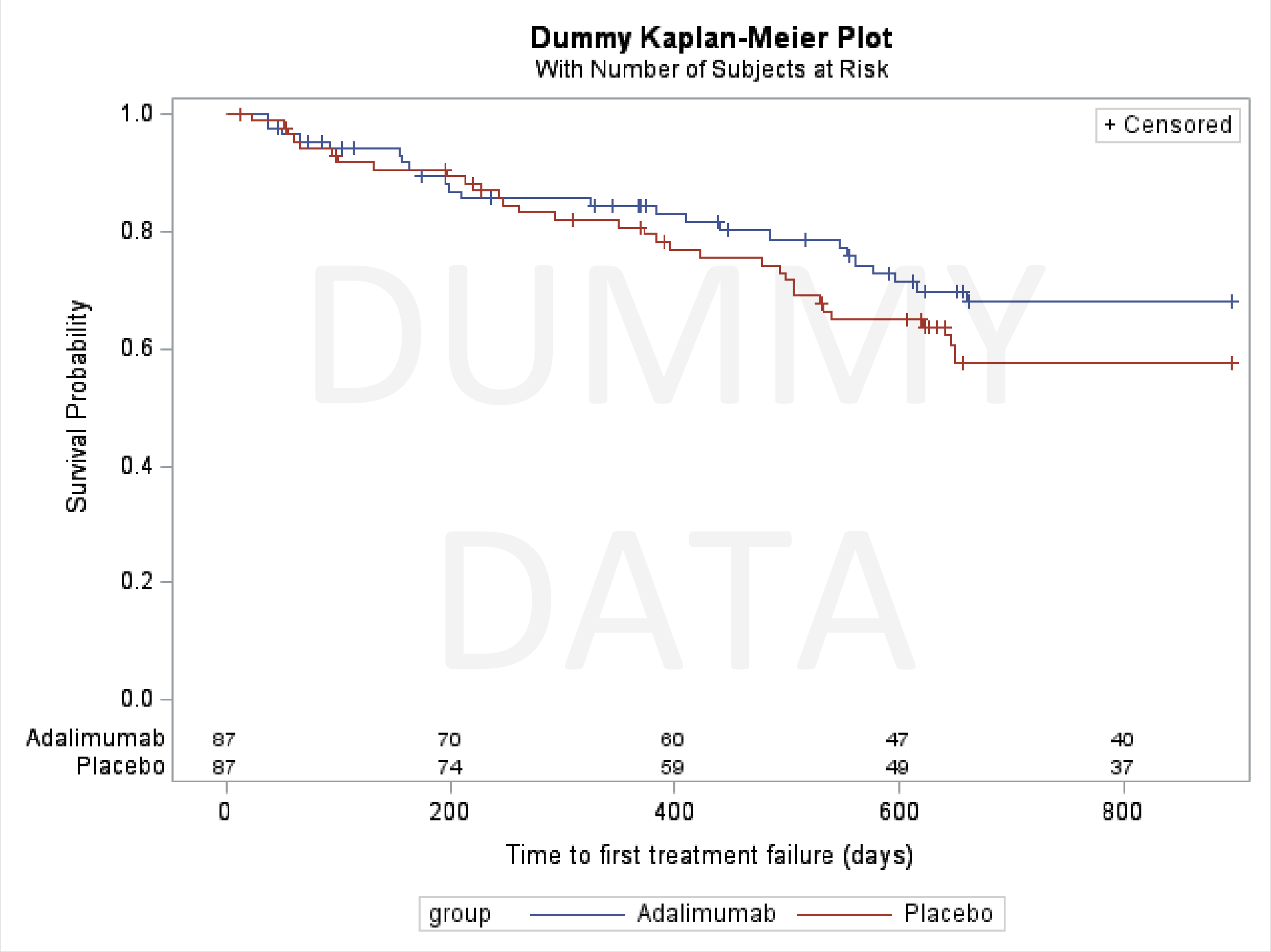

Notes:
Data are only included from RCT period #1

Table T2 Example table for longitudinal outcomes

		**Adalimumab (n=XX)**			
**TRI period #1**	Baseline<br>Week 0<br>Week 16				
		**Randomised to adalimumab (n=XX)**	**Randomised to placebo (n=XX)**	**Effect (95% CI)**	**p-value**
**RCT period #1**	Week 12<br>Week 24<br>Week 36<br>…				
Overall treatment effect estimate					
		**Treatment failure in RCT period #1 (n=XX)**	**Treatment failure in RCT period #1 (n=XX)**		
**TRI period #2**[1]	Week 0<br>Week 16				
		**Switched to placebo (n=XX)**	**Switched to adalimumab (n=XX)**		
**RCT period #2**	Week 12<br>Week 24<br>Week 36<br>…				
		**Treatment failure in RCT period #2**	**Treatment failure in RCT period #2**		
**TRI period #3**[1]	Week 0<br>Week 16				
		**Switched to adalimumab (n=XX)**	**Switched to placebo (n=XX)**		
**RCT period #3**	Week 12<br>Week 24<br>Week 36<br>…				

Notes:
Data are presented as median (IQR), mean (SD) or n (%).
CI=confidence interval
[1] In these periods all participants receive adalimumab.

Table T3 Adverse events and serious adverse events

	**Received adalimumab**				**Received placebo**			
	No. of AEs	No. of SAEs	AEs/100 patient-years	SAEs/100 patient-years	No. of events	No. of SAEs	AEs/100 patient-years	SAEs/100 patient-years
**TRI period #1**	**Received adalimumab (n= XX)**				**Received placebo (n=XX)**			
Any adverse event								
Event 1								
Event 2								
…								
**RCT period #1**	**Received adalimumab (n= XX)**				**Received placebo (n=XX)**			
Any adverse event								
Event 1								
Event 2								
…								
**TRI period #2**[1]	**Received adalimumab in RCT period #1 (n= XX)**				**Received placebo in RCT period #1 (n=XX)**			
Any adverse event								
Event 1								
Event 2								
…								
**RCT period #2**	**Received adalimumab (n= XX)**				**Received placebo (n=XX)**			
Any adverse event								
Event 1								
Event 2								
…								
**TRI period #3**[1]	**Received adalimumab in RCT period #2 (n= XX)**				**Received placebo in RCT period #2 (n=XX)**			
Any adverse event								
Event 1								
Event 2								
…								
**RCT period #3**	**Received adalimumab (n= XX)**				**Received placebo (n=XX)**			
Any adverse event								

Event 1		
Event 2		
…		
**Events across all TRI/RCT periods**	**Received adalimumab (n= XX)**	**Received placebo (n=XX)**
Any adverse event		
Event 1		
Event 2		
…		

Notes:
Numbers of events that were classified as related will be described
[1] In these periods all participants receive Adalimumab. However data are presented by the treatment they were randomised to in the previous RCT period
