# Supplementary material for: Adalimumab versus placebo as add-on to standard therapy for non-infectious uveitis in the United Kingdom: the ASTUTE randomised controlled trial with treatment run-in
Source: eClinicalMedicine. 2026 Oct 8;101:104240. doi: 10.1016/j.eclinm.2026.104240 (PMC13677017; doi:10.1016/j.eclinm.2026.104240)
Supplement: Supplementary file 5 [file mmc3.docx]

# Supplementary file: The ASTUTE study investigators

This is the full list of ASTUTE study investigators (the collaborative group named on the title page as “on behalf of the ASTUTE study investigators”). None of the individuals below is a named author on the title page.

| **First name(s)** | **Surname** | **Affiliation** |
| --- | --- | --- |
| Simon | Epps | Bristol Eye Hospital, University Hospitals Bristol and Weston NHS Foundation Trust |
| Krishna | Naruganahalli | Bristol Eye Hospital, University Hospitals Bristol and Weston NHS Foundation Trust |
| Natasha | Clark | Bristol Eye Hospital, University Hospitals Bristol and Weston NHS Foundation Trust |
| Laura | Bendrey | Bristol Eye Hospital, University Hospitals Bristol and Weston NHS Foundation Trust |
| Helen | Devonport | Bradford Teaching Hospitals NHS Foundation Trust |
| Sridevi | Rajasekaran | Bradford Teaching Hospitals NHS Foundation Trust |
| Hassan | Ahmed | Bradford Teaching Hospitals NHS Foundation Trust |
| Edward | Hughes | Sussex Eye Hospital, University Hospitals Sussex NHS Foundation Trust |
| Gabriela | Ortiz | Sussex Eye Hospital, University Hospitals Sussex NHS Foundation Trust |
| Dimitra | Gkika | Sussex Eye Hospital, University Hospitals Sussex NHS Foundation Trust |
| Erika | Damato | Cambridge Eye Unit, Cambridge University Hospitals NHS Foundation Trust |
| Jonathan | Virgo | Guy’s and St Thomas’ NHS Foundation Trust |
| Tasanee | Braithwaite | Guy’s and St Thomas’ NHS Foundation Trust |
| Paraskevi | Riga | Guy’s and St Thomas’ NHS Foundation Trust |
| Kanchan | Bhan | The Leeds Teaching Hospitals NHS Trust |
| Clare | Howcroft | The Leeds Teaching Hospitals NHS Trust |
| Mike | Stockton | The Leeds Teaching Hospitals NHS Trust |
| Michael | Hisaund | University Hospitals of Leicester NHS Trust |
| Periyasamy | Kumar | University Hospitals of Leicester NHS Trust |
| Lauren | Rybicki | University Hospitals of Leicester NHS Trust |
| Nima | Ghadiri | St Paul’s Eye Unit, NHS University Hospitals of Liverpool Group |
| Goncalo | Almeida | Maidstone and Tunbridge Wells NHS Trust |
| Hanna | Bimah | Maidstone and Tunbridge Wells NHS Trust |
| Idris | Haider | Maidstone and Tunbridge Wells NHS Trust |
| Carlos | Pavesio | Moorfields Eye Hospital NHS Foundation Trust |
| Sumita | Phatak | Moorfields Eye Hospital NHS Foundation Trust |
| Gerry | Clare | Moorfields Eye Hospital NHS Foundation Trust |
| Colin | Jones | Norfolk and Norwich University Hospitals NHS Foundation Trust |
| Chin Pey | Yap | Norfolk and Norwich University Hospitals NHS Foundation Trust |
| Archana | Pradeep | Nottingham University Hospitals NHS Trust |
| Elizabeth | Mathew | Oxford Eye Hospital, Oxford University Hospitals NHS Foundation Trust |
| Theodora | Gkika | Oxford Eye Hospital, Oxford University Hospitals NHS Foundation Trust |
| Shabnam | Raji | Oxford Eye Hospital, Oxford University Hospitals NHS Foundation Trust |
| Janette | Savage | Oxford Eye Hospital, Oxford University Hospitals NHS Foundation Trust |
| Katharine S | Sears | Northern General Eye Centre, Sheffield Teaching Hospitals NHS Foundation Trust |
| Katherine | Wheeler | Northern General Eye Centre, Sheffield Teaching Hospitals NHS Foundation Trust |
| Farid | Afshar | University Hospital Southampton NHS Foundation Trust |
| Catherine | Murphy | University Hospital Southampton NHS Foundation Trust |
| Ahmed | Saad | South Tees Hospitals NHS Foundation Trust |
| Kerry | Hebbron | South Tees Hospitals NHS Foundation Trust |
| Balini | Balasubramaniam | University Hospitals Birmingham NHS Foundation Trust |
| Sawsan | Elsheikh | University Hospitals Birmingham NHS Foundation Trust |
| Helen | Hawkins | University Hospitals Birmingham NHS Foundation Trust |
| Yvonne | Anyango | University Hospitals Birmingham NHS Foundation Trust |
| Richard | Gale | York and Scarborough Teaching Hospitals NHS Foundation Trust |
| Christine | O'Dwyer | York and Scarborough Teaching Hospitals NHS Foundation Trust |
| Sally | Gilroy | York and Scarborough Teaching Hospitals NHS Foundation Trust |
| Elizabeth | McCullagh | University Hospitals Bristol and Weston NHS Foundation Trust |
| Shakeel | Herwitker | Liverpool University Hospitals NHS Foundation Trust |
| Suhail | Kidi | NHS University Hospitals of Liverpool Group |
| Abby | O'Connell | Bristol Trials Centre, Bristol Medical School, University of Bristol |
| Mae | Hazell | Bristol Trials Centre, Bristol Medical School, University of Bristol |
| Sophie | Coke-Norris | Bristol Trials Centre, Bristol Medical School, University of Bristol |
| Laura | Waugh | Bristol Trials Centre, Bristol Medical School, University of Bristol |
| Robin | Wickens | Bristol Trials Centre, Bristol Medical School, University of Bristol |
| Beth | Howden | Bristol Trials Centre, Bristol Medical School, University of Bristol |
| Isabel | Druiff | Bristol Trials Centre, Bristol Medical School, University of Bristol |
| Sian | Wells | Bristol Trials Centre, Bristol Medical School, University of Bristol |
| Kelsey | Harrendence | Bristol Trials Centre, Bristol Medical School, University of Bristol |
| Rachel | Todd | Bristol Trials Centre, Bristol Medical School, University of Bristol |
| David | Hutton | Bristol Trials Centre, Bristol Medical School, University of Bristol |
| Jana | Kirwan | Bristol Trials Centre, Bristol Medical School, University of Bristol |
| Jo | Berkeley | Bristol Trials Centre, Bristol Medical School, University of Bristol |
| Vaishali | Thaker | Bristol Trials Centre, Bristol Medical School, University of Bristol |
| Kate | Attfield | Nuffield Department of Clinical Neurosciences, University of Oxford |
| Subita Balaram | Kuttikkatte | Nuffield Department of Clinical Neurosciences, University of Oxford |
| Karleigh | Kelso | Belfast Ophthalmic Reading Centre, Queen’s University Belfast |
| Clare | Newell | Belfast Ophthalmic Reading Centre, Queen’s University Belfast |
| Barbra | Hamill | Belfast Ophthalmic Reading Centre, Queen’s University Belfast |

Total investigators listed: 69.
